# Dimerization and Transactivation Domains as Candidates for Functional Modulation and Diversity of Sox9

**DOI:** 10.1371/journal.pone.0156199

**Published:** 2016-05-19

**Authors:** Marcos Tadeu Geraldo, Guilherme Targino Valente, Rafael Takahiro Nakajima, Cesar Martins

**Affiliations:** 1 Integrative Genomics Laboratory, Department of Morphology, Institute of Biosciences, Sao Paulo State University–UNESP, Botucatu, SP, 18618–000, Brazil; 2 Systems Biology and Genomics Laboratory, Department of Bioprocess and Biotechnology, Agronomical Science Faculty, Sao Paulo State University–UNESP, Botucatu, SP, 18610–307, Brazil; University of Saarland Medical School, GERMANY

## Abstract

Sox9 plays an important role in a large variety of developmental pathways in vertebrates. It is composed of three domains: high-mobility group box (HMG box), dimerization (DIM) and transactivation (TAD). One of the main processes for regulation and variability of the pathways involving Sox9 is the self-gene expression regulation of Sox9. However, the subsequent roles of the Sox9 domains can also generate regulatory modulations. Studies have shown that TADs can bind to different types of proteins and its function seems to be influenced by DIM. Therefore, we hypothesized that both domains are directly associated and can be responsible for the functional variability of Sox9. We applied a method based on a broad phylogenetic context, using sequences of the HMG box domain, to ensure the homology of all the Sox9 copies used herein. The data obtained included 4,921 sequences relative to 657 metazoan species. Based on coevolutionary and selective pressure analyses of the Sox9 sequences, we observed coevolutions involving DIM and TADs. These data, along with the experimental data from literature, indicate a functional relationship between these domains. Moreover, DIM and TADs may be responsible for the functional plasticity of Sox9 because they are more tolerant for molecular changes (higher Ka/Ks ratio than the HMG box domain). This tolerance could allow a differential regulation of target genes or promote novel targets during transcriptional activation. In conclusion, we suggest that DIM and TADs functional association may regulate differentially the target genes or even promote novel targets during transcription activation mediated by Sox9 paralogs, contributing to the subfunctionalization of Sox9a and Sox9b in teleosts.

## Introduction

The Sox proteins are involved in many developmental processes [1] across different metazoan groups such as insects, nematodes, amphibians, reptiles, birds and mammals [2]. These proteins are members of a non-canonical family of DNA-binding domain known as the high-mobility group box (HMG box), consisting of approximately 80 residues in a twisted L-shape structure that binds to the minor groove of DNA [3]. The HMG proteins were discovered as acid-extractable chromatin components of high electrophoretic mobility [4], and its superfamily has been divided into three unrelated families, known as HMGA, HMGB and HMGN based on the systematic reference to their different DNA-binding domains [5, 6]. Canonical HMGs are architectural proteins and do not contain transactivation domains, in contrast to some transcription factors with non-canonical HMG-motifs that contain transactivation domains [7]. The Sox family proteins contain only one non-canonical HMG box domain with only about 20% sequence identity to the canonical DNA-binding domain of HMGB proteins [8]. The first Sox protein discovered was Sry (Sex determining region Y) [9] and since then around 20 Sox proteins have been identified in mice and humans. In general, the Sox proteins have been grouped based on the sequence and structural similarity of their HMG box domain [10].

Sox9 (Sex Determining Region Y-Box 9), a member of the SoxE subgroup (comprised of Sox8, Sox9 and Sox10), plays an important role in a variety of developmental processes of mesoderm (cartilage [11, 12] and male gonad [13–15]), ectoderm (central nervous system [16, 17], neural crest [18, 19] and retina [20]) and endoderm (pancreas [21, 22], liver [22] and intestine [23]). Therefore, Sox9 is a broad regulator and it has been related to different developmental disorders. For instance, heterozygous Sox9 mutations cause campomelic dysplasia, a syndrome characterized by skeletal malformations and associated, in some cases, with XY sex reversal [13, 24]. In addition, it is related to some acquired diseases, such as fibrosis [25], sclerosis [26], tumorigenesis [27] and cancer [28].

Although Sox9 is encoded by a single-copy gene in most vertebrates, studies have shown the existence of duplicate copies in teleosts [29] (hereafter named as *Sox9a* and *Sox9b*). According to recent data of gene expression in *Danio rerio* (zebrafish) and *Oryzias latipes* (medaka), the two copies seem to have undergone a lineage-specific subfunctionalization process [2, 30].

In addition to the HMG box domain, Sox9 is composed of a self-dimerization domain (DIM) [31, 32], and two transactivation domains (TAD)–K2 and PQS [29, 33, 34]. In humans and mice, there is an additional TAD (known as PQA), which enhances the PQS transactivation activity but it is unable to activate transcription alone [34]. Additionally, since PQA is exclusive of mammals, it has been suggested that this domain is related to the SRY sex-determining mechanism of these organisms [35]. These domains from Sox9 have also been found in Sox8 and Sox10, and the annotations used for them are still nonconsensual in the literature. Therefore, we propose the aforementioned nomenclature compiled from different studies related to the SoxE proteins [31, 36–38].

Studies have evaluated the effects of amino acids substitutions, deletions, frameshifts and truncations in Sox9 activity and have determined the functions of each domain [29, 31–34, 37]. Some questions arose based on these works: (i) Is there a functional and/or structural relationship between the domains?; (ii) Which domains can be responsible for the functional diversity observed in Sox9? Regarding the second question, we hypothesized that DIM and TADs are directly associated and responsible for the functional variability observed in Sox9. The premise for this hypothesis is that these domains may interfere and/or mediate the interactions with a distinct number of proteins. We believe that this binding plasticity could be a good regulatory strategy for transcriptional activation and repression.

A detailed analyses of coevolutions of Sox9 residues (including Sox9a and Sox9b) along with selective pressure calculations (Ka/Ks ratio) suggested that DIM and TADs are functionally or structurally associated and can be candidates to modulate the variability of Sox9 function.

## Results

### Homology relationships of *Sox9*

The phylogenetic reconstruction of the HMG box sequences showed the SoxE subgroup with three well-supported branches comprising Sox8, Sox9 and Sox10 (S1 Fig).

**Fig 1:**
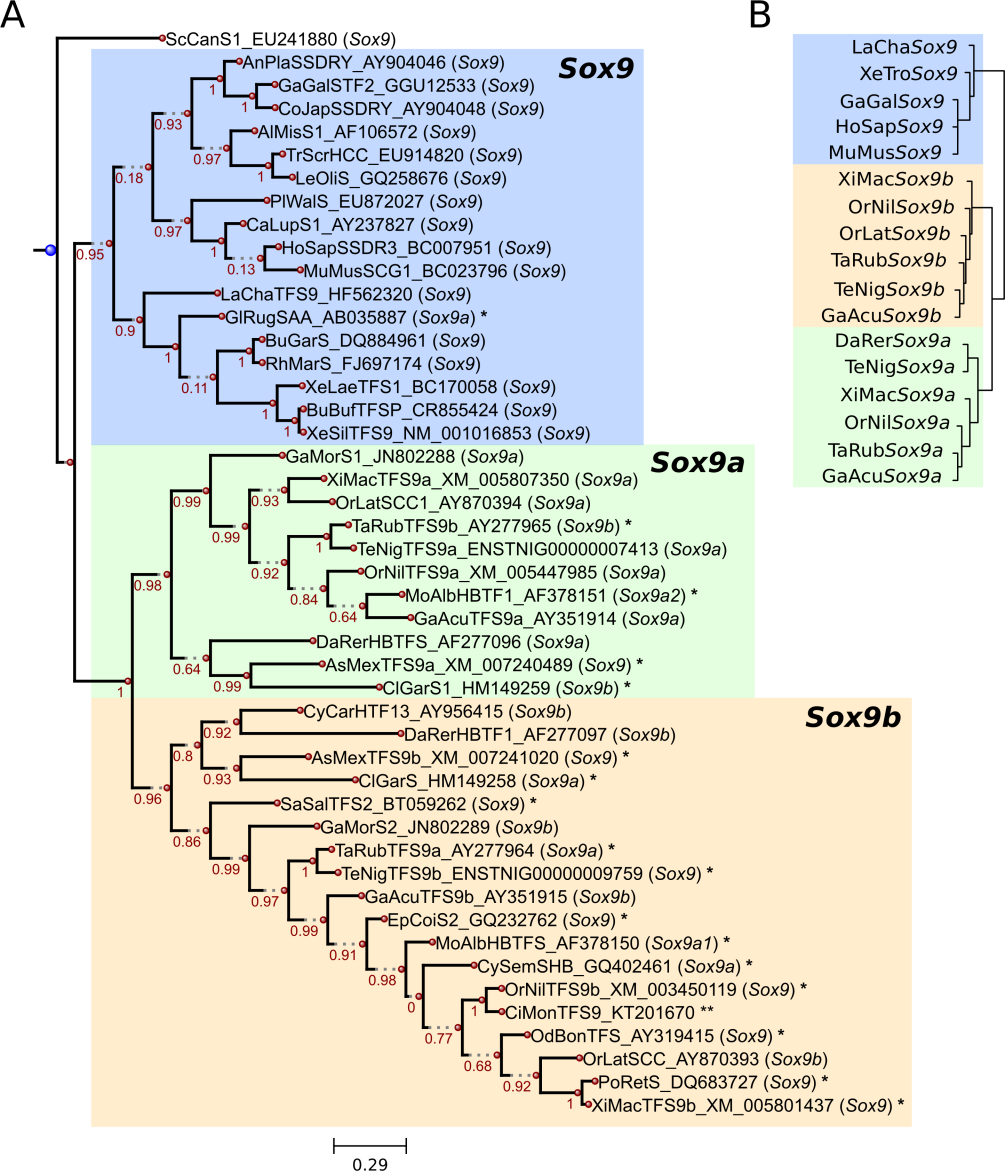
Evolutionary relationships among the *Sox9* vertebrate sequences. (A) Phylogeny of the Sox9 multiple sequence alignment using the maximum likelihood method from the PhyML program. The aLRT (SH-like) values of branch support are shown. The corresponding clades for *Sox9* (blue), *Sox9a* (green) and *Sox9b* (orange) are indicated. The database entries (accession numbers from GenBank or Ensembl) are shown with their corresponding annotations in the database in parenthesis. The single asterisks (*) indicates the divergences between our results and the gene annotation in the corresponding database entry, whereas the double asterisks (**) indicates the sequence obtained from the genome walking technique. The scale bar below the phylogenetic tree indicates the average number of nucleotides substitutions per site. (B) Dendogram of the synteny analysis, based on the closest genetic markers: the clades corresponding to *Sox9* (blue), *Sox9a* (green) and *Sox9b* (orange) were obtained from the hierarchical manhattan clustering method, implemented in the R software.

The sequences from the *Sox9* branch were selected for a more detailed evolutionary analysis. An exclusive *Sox9* phylogeny was generated and clearly showed three main clades, corresponding to *Sox9*, *Sox9a* and *Sox9b*, and only the teleost species exhibited the duplicate copies (Fig 1A).

Some inconsistencies were perceived comparing the gene annotations from the searched databases with our results. For instance, *Sox9a* and *Sox9b* were reversed annotated in *Takifugu rubripes* (fugu) and *Clarias gariepinus* (african sharptooth catfish); moreover, these genes were assigned as *Sox9a2* and *Sox9a1*, respectively, in *Monopterus albus* (rice field eel). Other inconsistencies were observed and are shown in Fig 1A. The retrieved sequence from *Cichla monoculus* (peacock bass) was ortholog to *Sox9b*. The degenerate primers used may have annealed only to *Sox9b*, precluding the detection of *Sox9a*.

To overcome some undesired incongruences from phylogenetic analysis, possibly related to the high evolutionary rate of substitutions in sequences, an approach based on the combination of phylogenetic results with genomic array of genes can be helpful to corroborate evolutionary relationships. We already used this approach in a study of the *Foxl2* gene [39]. Therefore, an additional support for the *Sox9* evolutionary relationships was determined using the synteny information of specific genetic markers combined with a hierarchical clustering (Fig 1B) (S1 Table). We observed *Sstr2* as the common genetic marker of the *Sox9* single and duplicate copies in all the vertebrate groups analyzed, and *Rasd1* and *Adap1* as the specific markers of *Sox9a* and *Sox9b* in almost all the teleost groups analyzed. The specific markers of *Sox9a* were primarily *Cops3* and *Pemt* but other makers were also observed, such as *Lmf1*, *Vstm4b*, *Lrrc18b* and *Mapk8b*. The main markers of *Sox9b* were *Abca3*, *Tmc6a*, *Notum1a*, *Myadml2*, *Mafg*, *Pcyt2* and *Cant1a*. An exception was zebrafish *Sox9b*, in which none of those markers were found.

### Intra-molecular coevolution within and among the Sox9 domains

**Fig 2:**
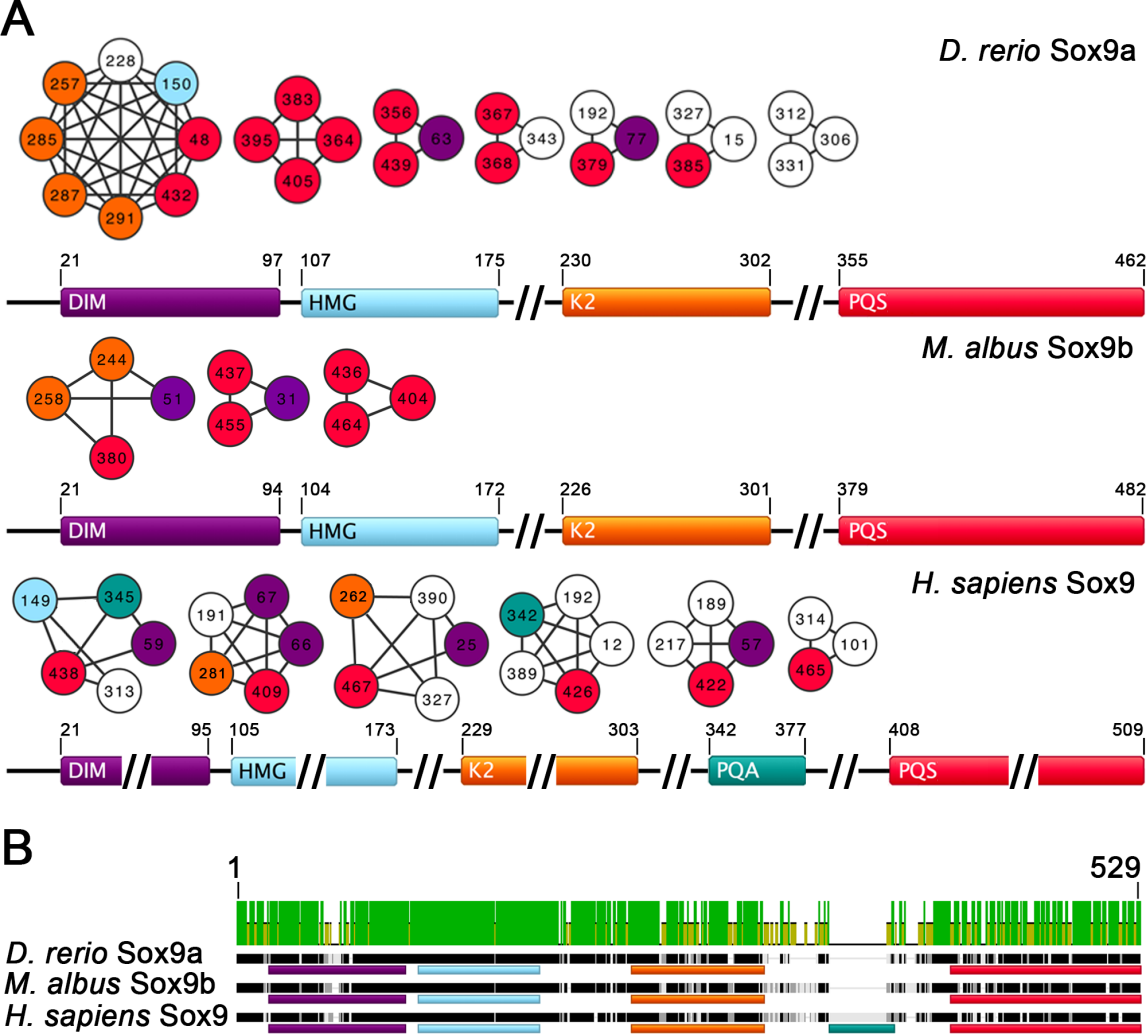
Network of coevolutions in the Sox9 proteins. (A) The coevolutions are shown using *D*. *rerio* Sox9a, *M*. *albus* Sox9b and *H*. *sapiens* Sox9 as the reference sequences. The graph nodes indicate the amino acid with its corresponding position number in the reference sequence. The edges (lines) connect the pair of coevolving amino acids inferred from the CAPS program. Each amino acid is colored based on its localization in the corresponding Sox9 domain: DIM (purple), HMG box (light blue), K2 (orange), PQA (dark cyan) and PQS (red). The range (begin-end) of each domain is also depicted in the scheme. The sign for truncated regions (//) is used for fitting the figure dimensions. (B) The Sox9 multiple protein sequence alignment is indicated. The color of each domain follows the description aforementioned. Upper green bars evidence high conserved sites.

The inference of intra-molecular coevolutions indicated possible relations among the DIM, K2 and PQS domains in all the Sox9 homologs analyzed (Fig 2). Sox9a showed a large number of intra and inter-domain coevolutions, especially in K2 and PQS, with a large group of mutual correlations. Although less representative, Sox9b showed a similar profile. Even though Sox9 showed a divergent profile, it also evidenced relations among DIM, K2 and PQS but with a lower degree compared to Sox9a. Besides, Sox9 showed a higher number of coevolving residues that are located outside the annotated domains. Finally, the highly conserved HMG box domain exhibited the lowest number of coevolving amino acids.

The results of Ka/Ks calculation showed that all domains are under purifying selection; K2 had the highest Ka/Ks ratio followed by PQS and DIM, whereas HMG box had the lowest value (S2 Table).

## Discussion

### Paralogy of *Sox9a* and *Sox9b*: phylogenetic and syntenic approaches

The divergence in gene annotation and the lack of protein tertiary structure of Sox9 difficult the determination of correlations between evolutionary events and their functional implications. Nakamura et al. [30] already settled, based on genomic synteny analyses, the nomenclature of *Sox9* paralogs as *Sox9a* and *Sox9b*. However, these analyses were restricted to a small number of sequences and no phylogeny was inferred. In contrast, we gathered a representative data set of *Sox9* sequences, based on a search over different vertebrate genomes. The imbalance in the number of sequences for *Sox9a* and *Sox9b* was due to the data availability at GenBank because only one of the paralog forms was found in the database for some species. Despite of this imbalance, it is evident, by the current data from the searched genomes, that the duplicate copies of Sox9 (referred to as Sox9a and Sox9b) are unique to the teleost lineage. Therefore, they were probably originated from the specific whole genome duplication occurred in this group [40, 41], in agreement with the suggestion of previous studies [2, 30, 42]. Moreover, we used the synteny information from *Sox9* to generate a dendogram using a hierarchical-clustering algorithm, and the results also supported the paralogy of *Sox9a* and *Sox9b*, reinforcing the conclusions aforementioned.

### DIM and TADs as important regions for functional plasticity

Intra-coevolutionary inferences represent an important approach for understanding the evolution and function of proteins because structural and functional relationships or physical interactions between amino acids can be the main causes of their coevolution [43]. Mutations at functionally related sites can change the selective constraints, so coevolution inference is an important complement for molecular selection analysis [43]. Furthermore, it has been observed that physically distant coevolving sites can be essential to maintain the structural and functional stability of the protein [44].

Our coevolution results show possible relationships among all the Sox9 domains from the single and duplicate copies but especially among DIM, K2 and PQS. Primarily, we observed a large group of coevolutions in Sox9a. Studies with single and frameshift mutations/deletions in TADs showed that, for maximum transactivation, K2 and PQS are critical for the proper transcriptional activation/repression of target genes [29, 34]. Therefore, this result is in agreement with our coevolution data that showed pairs of coevolving residues between K2 and PQS. Furthermore, we showed that K2 and PQS are under purifying selection and have the most relaxed selective pressure (primarily K2), reflecting a higher fixation of mutations. In general, most of the protein-protein interactions involved to orchestrate the assembly of the transcriptional machinery are mediated by TAD [45]; therefore, our results of coevolution and selective pressure suggest that this domain can be a good candidate for transcriptional regulation.

The transactivation process, however, depends on the oligomeric state of Sox9. It has been observed that Sox9 can bind to response elements of target genes as a homodimer or monomer, associating with different protein partners to activate or repress the transcriptional machinery [31, 32, 46, 47]. It has been observed that mutations within DIM abrogated the dimerization and ceased or reduced the transactivation activity played by Sox9 for target chondrogenic and sex-determining genes, whereas other functional features of the protein remained unaltered [32]. The most dramatic effect was observed in a 66–75 deletion within DIM, affecting the promoter activation of Amh, a sex-determing gene that only requires a Sox9 monomer for its activation, indicating that DIM is not limited to the dimerization function. We observed in the human Sox9 the residues 66 and 67 from DIM coevolving with the residues 281 and 409 from K2 and PQS, respectively. Altogether, these experimental data and our coevolutionary results indicate that DIM, apart from its dimerization function, may influence directly the transactivation activity.

Sock et al. [32] considered unlikely the direct interference between DIM and TAD (primarily PQS and PQA) because they are physically distant, considering the protein sequence. However, as we mentioned earlier, distant sites in a protein can be functionally or structurally associated; therefore, the transcriptional activation/repression of Sox9 target genes could be strictly related to the TAD affinity, influenced by DIM, to its binding partners. We suppose that these domains are physically close in the tertiary structure, or have some structural feature that a change in one domain may affect the other. This scenario agrees with our coevolutionary and selective pressure analyses, corroborating the relationship between DIM and TAD as a good strategy for the transcriptional regulation of pathways involving Sox9.

The HMG box domain, in contrast, had the lowest rate of mutational fixations and the lowest number of coevolving residues. The high conservation of the HMG box in the Sox9 sequences may be a result of its binding pattern [48], generating a significant constraint in the protein conformation. An exception was observed in Sox9a with a large group of amino acids from K2 and PQS coevolving with one amino acid from the HMG box. However, no experimental analysis indicates a direct functional or structural involvement between HMG box and TADs. Indeed, mutational analyses within the HMG box have shown that the transactivation activity is only abrogated as a consequence of a reduced affinity or inability of Sox9 to bind DNA [34, 49, 50].

Finally, *Sox9a* and *Sox9b*, after the duplication event in teleosts, seem to have undergone a lineage-specific subfunctionalization, indicated by the similar tissue expression patterns in zebrafish *Sox9a* and medaka *Sox9b* [2, 30]. Although it requires additional data from other teleost species, we suggest that the functional association between DIM and TADs differentially regulate the target genes or even promote novel targets during the transcriptional activation mediated by the Sox9 paralogs. Therefore, this association makes DIM and TADs more flexible to promote a fine gene expression regulation along the subfunctionalization process.

## Conclusion

This study correlates evolutionary analyses with functional implications for the Sox9 proteins. First, we reinforce that the event of genome duplication specific of teleosts is the evolutionary process that triggered the evolution of *Sox9a* and *Sox9b*. We also suggest that DIM and TADs are candidates for functional modulation and variability of the Sox9 single and duplicate copies (Sox9a and Sox9b) in vertebrates, allowing the use of well-coordinated regulation strategies that can operate within and among these domains. Furthermore, our study correlates evolutionary analyses with functional implications of Sox9 and highlights that DIM and TAD can be additional players involved in the regulation and diversification of the Sox9 activity. Finally, this strategy could be also used by other transcription factors, such as SoxE proteins.

## Materials and Methods

### Sequence acquisition and alignment procedures

#### HMG box domain sequences

To obtain a significant number of sequences and ensure the use of Sox9 homologs, a retrieval methodology based on the HMG box domain was used. This approach ensures a more accurate identification of homologous sequences to the gene of interest and facilitates the choice of outgroup.

Protein sequences of the HMG box family were collected from a large number of metazoans in the Pfam database [51] (accession: PF00505), totaling 4,921 sequences relative to 657 species (S3 Table). Each sequence, identified by an UniProt (http://www.uniprot.org) accession number, was converted to GenBank accession numbers utilizing the gbreader software (Razente HL, Braz ASK, Scott LPB (unpublished)).

To avoid redundancy and ensure the use of representative data, a clustering methodology was performed using the CD-HIT software [52]. The clustering cut-off applied was 99% identity to the HMG box domain (high cut-off because the HMG box domain is highly conserved in metazoan); afterwards, only one representative protein from each cluster was selected for the phylogenetic analysis. This previous procedure resulted in a data set of 779 sequences that were aligned based on a Hidden Markov Model (HMM) profile of the HMG box domain (final alignment length = 69 sites), using the HMMER v.3 software [53].

#### Sox9 sequences

In addition to the sequences obtained from the UniProt database, Sox9 sequences were obtained based on TBLASTN searches over the nucleotide collection from GenBank (http://www.ncbi.nlm.nih.gov), and the vertebrate genomes from the Ensembl database (http://www.ensembl.org/) [54].

Additionally, an experimental procedure was employed to retrieve the *Sox9* sequence from the neotropical cichlid peacock bass *Cichla monoculus* collected from Balbina hydroelectric station lake, Presidente Figueiredo-AM, Brazil (3°09’57”S, 59°54’44”W), according to the Brazilian laws for environmental protection (wild collection permit, ICMBio 22984–1 e 32556–2). Voucher specimens (10816-female; 10850-male) were included in the fish collection of INPA-National Institute for Amazonian Researchs, Manaus/AM. The experimental procedure on the teleost was conducted according to the international guidelines of Sao Paulo State University and approved by the Institutional Animal Care and Use Committee (IACUC) (Protocol no. 34/08—CEEA/IBB/UNESP), and the tissue samples are available at Integrative Genomics Laboratory of Sao Paulo State University under the registration numbers 10816 and 10850. The animals were euthanized through immersion in a water bath with benzocaine 250 mg/liter during 10 minutes. The genomic DNA was extracted from muscle and liver tissues using the phenol-chloroform method [55]. *Sox9* was amplified by PCR (polymerase chain reaction) using degenerate primers (F—5’ CARGTNYTNAARGGNTAYGA 3’ and R—5’ CCANARYTTNCCNARNGTYTT 3’) constructed with the Primer3Plus software [56]. For the construction of these primers, Sox9 protein sequences from different vertebrate species were retrieved based on a BLASTP search at the Expasy Proteomic Server (http://ca.expasy.org/), using the Sox9a sequence from *Oreochromis aureus* (EU373500) as query. These sequences were aligned using ClustalW [57], and the primers were constructed for the most conserved regions. The amplicons were 195 base pair (bp), and the full gene sequence (3,327 bp) was obtained by the genome walking technique, using the Genome Walker™ kit (Clontech) according to the manufacturer's protocol. All amplicons were cloned in p-GEM-T plasmid vectors (Promega), and the corresponding clones were sequenced with an ABI Prism 3100 DNA sequencer (Perkin-Elmer), using ABI Prism Big Dye Terminator Cycle Sequencing Ready Reaction kits (Perkin-Elmer). Finally, a prediction of introns, exons and amino acid sequences were performed using the online program Softberry FGENESH (http://www.softberry.com/).

#### Phylogenetic analyses: HMG box, SoxE and Sox9

Three phylogenetic analyses were conducted separately: (i) HMG box, (ii) SoxE and (iii) *Sox9*. Based on the phylogeny of the HMG box domain, the corresponding branch for the subgroup E was determined (S1 Fig). Within this subgroup, the clustered amino acid sequences for *Sox8*, *Sox9* and *Sox10* were retrieved and aligned using the Mafft algorithm [58]. A maximum likelihood phylogeny was generated to allow the precise identification of the Sox9 homologs and guide the appropriate choice of outgroup. Afterwards, the Sox9 sequences were aligned based on their codons using the Mafft algorithm allocated on the GUIDANCE web server [59]. Finally, the reference data set for the *Sox9* phylogeny was composed of 47 nucleotide sequences, and the alignment length included 2,049 sites (S1 Text). The phylogenetic analysis was performed using only complete and putative *Sox9* sequences.

The choice of the best-fit model of evolution was performed with ProtTest3 [60] and Jmodeltest [61] for the amino acid and nucleotide sequences, respectively.

The phylogenetic reconstruction of *Sox9* was determined by the maximum likelihood method, implemented in the Phyml v3.0.1 [62] software, using the aLRT (SH-like) reliability test [63] (other parameter details, see S4 Table). The visualization and the final tree edition were performed using FigTree v1.3.1 (http://tree.bio.ed.ac.uk/software/figtree/) and ETE2 [64].

### Sox9 synteny analysis

To provide an additional support to the homology of the *Sox9* sequences and understand the composition/organization of the genes in the proximity of *Sox9*, the syntenic regions were analyzed in species which genomic information is available at the Genomicus browser [65]. A matrix was built based on the presence and absence of each genetic marker considered in the proximity of the *Sox9* single and duplicate copies. Latter, a hierarchical clustering was performed based on the this matrix information (S1 Table), using the manhattan method available in the R program [66].

### Intra-coevolution inference and Ka/Ks ratio calculation

The inference of intra-molecular coevolutions was done using the CAPS software [67]. CAPS measures the correlated evolutionary variation of amino acids to identify the coevolving site pairs. The performance and sensitivity of the method has been examined in different proteins [43] and, according to the authors, more accurate results can be achieved with long (≥ 20 sequences) and populated sequence alignments. The major advantage of CAPS over other methods of coevolutionary inference is the separation of phylogenetic linkage from structural and functional coevolution [43].

Each branch corresponding to *Sox9*, *Sox9a* and *Sox9b* was extracted and submitted, along with its aligned protein sequences, to the CAPS analysis. The complete alignment and subalignments, combined with the removal of specific phylogenetic clades, were generated following the CAPS automated protocol to exclude phylogenetic coevolutions [43]. Finally, a total of 1,000 alignment simulations were generated to remove the stochastic effects of the coevolutions inferred for the *Sox9* single and duplicate copies.

The Ka/Ks ratio calculations were conducted using the sequences of human Sox9 [GenBank:BC007951], zebrafish Sox9a [GenBank:NM_131643] and rice field eel Sox9b [GenBank:AF378150]. The calculations were based on the pairwise alignment (performed with Muscle [68]) of the HMG box, DIM, K2 and PQS domains, using the command line KaKs-Calculator v1.2 [69] with model selection parameter (S2 Table).

## Supporting Information

S1 Fig HMG box and SoxE phylogeny. Maximum-likelihood phylogenetic tree of the HMG box domain (A) and SoxE subgroup (B).
(PNG)

S1 Table Sox9 genetic markers. The important markers of *Sox9*, *Sox9a* and *Sox9b* are highlighted in blue, green and orange, respectively. The presence (1) or absence (0) of a specific marker is depicted.
(XLS)

S2 Table Ka/Ks calculation. Values of Ka/Ks for the Sox9 domains, including the p-value and model of evolution.
(PDF)

S3 Table Sequence retrieval. Amino acid sequences obtained from Uniprot database based on the HMG box domain.
(XLS)

S4 Table Maximum-likelihood reconstruction tree parameters. Data type, substitution model, tree searching operations, starting tree method and branch support are indicated.
(PDF)

S1 Text Sox9 multiple codon sequence alignment. Codon based alignment of all sequences used for *Sox9* phylogenetic inference.
(PDF)
