# Supplementary figures and images for: Dimerization and Transactivation Domains as Candidates for Functional Modulation and Diversity of Sox9
Source: PLoS One. 2016 May 19;11(5):e0156199. doi: 10.1371/journal.pone.0156199 (PMC4873142; doi:10.1371/journal.pone.0156199)

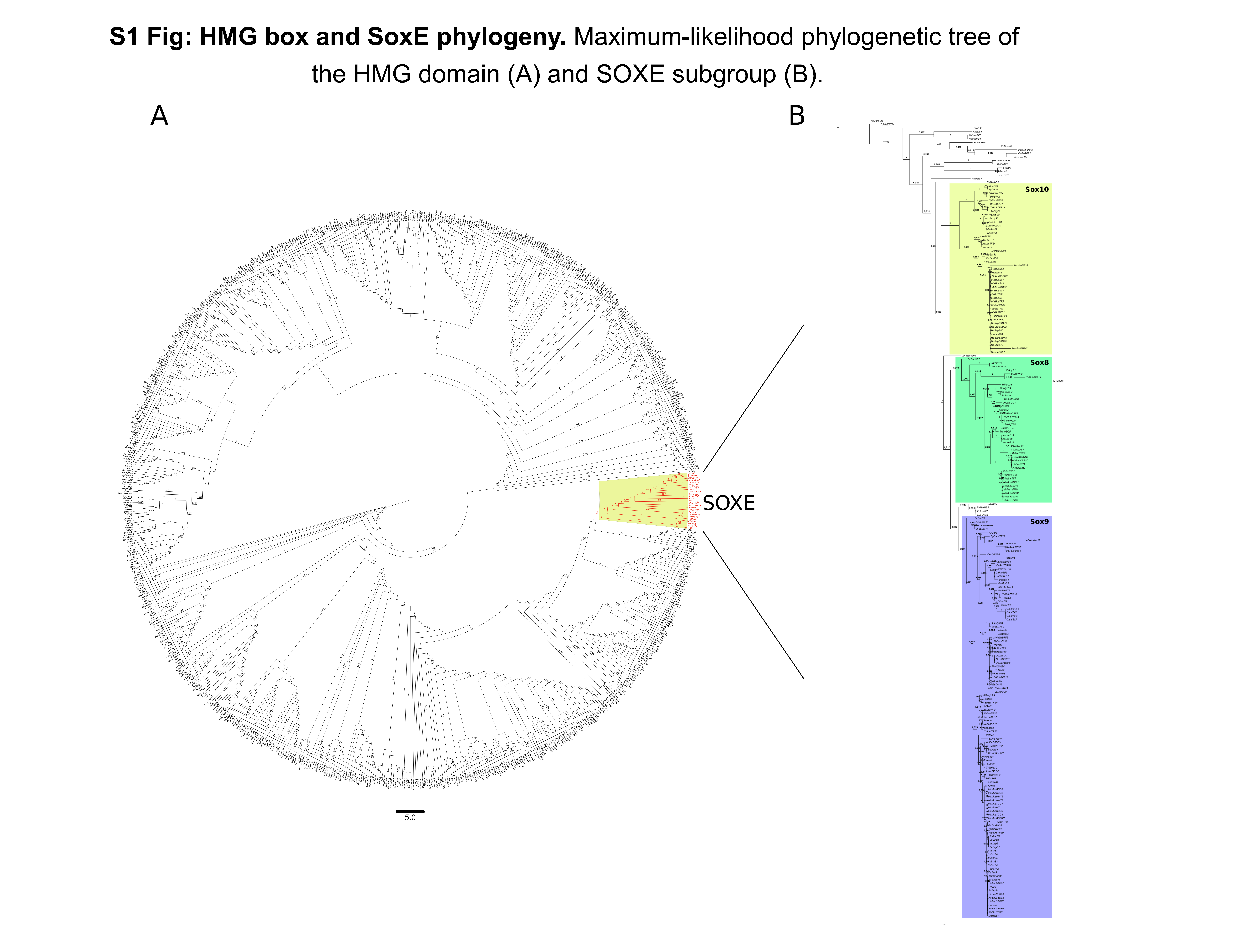

Supplement: S1 Fig — Maximum-likelihood phylogenetic tree of the HMG box domain (A) and SoxE subgroup (B). (PNG) [file pone.0156199.s001.png]
