# Supplementary material for: Dimerization and Transactivation Domains as Candidates for Functional Modulation and Diversity of Sox9
Source: PLoS One. 2016 May 19;11(5):e0156199. doi: 10.1371/journal.pone.0156199 (PMC4873142; doi:10.1371/journal.pone.0156199)
Supplement: S2 Table — Values of Ka/Ks for the Sox9 domains, including the p-value and model of evolution. (PDF) [file pone.0156199.s003.pdf]

S2 Table. Ka/Ks calculation. Values of Ka/Ks for the Sox9 domains, including the p-value and model of evolution.

Tests	Sp1	Sp2	Ka/Ks	P-Value(Fisher)	Result	Model
			**Sox9 and Sox9a**			
DIM	Danio rerio	Homo sapiens	0.0113724	**0**	**purifying selection**	F81
HMG	Danio rerio	Homo sapiens	0.00180382	**0**	**purifying selection**	F81
K2	Danio rerio	Homo sapiens	0.0433189	**1.41E-35**	**purifying selection**	HKY
PQS	Danio rerio	Homo sapiens	0.0213095	**0**	**purifying selection**	TN
			**Sox9 and Sox9b**			
DIM	Homo sapiens	Monopterus albus	0.016088	**0**	**purifying selection**	F81
HMG	Homo sapiens	Monopterus albus	0.00197404	**0**	**purifying selection**	F81
K2	Homo sapiens	Monopterus albus	0.0687748	**0**	**purifying selection**	TVM
PQS	Homo sapiens	Monopterus albus	0.0161906	**0**	**purifying selection**	TN
			**Sox9a and Sox9b**			
DIM	Danio rerio	Monopterus albus	0.011934	**6.48E-40**	**purifying selection**	F81
HMG	Danio rerio	Monopterus albus	0.001	**6.13E-27**	**purifying selection**	HKY
K2	Danio rerio	Monopterus albus	0.0467966	**0**	**purifying selection**	TVM
PQS	Danio rerio	Monopterus albus	0.0162497	**0**	**purifying selection**	TIM
