# Supplementary material for: Dimerization and Transactivation Domains as Candidates for Functional Modulation and Diversity of Sox9
Source: PLoS One. 2016 May 19;11(5):e0156199. doi: 10.1371/journal.pone.0156199 (PMC4873142; doi:10.1371/journal.pone.0156199)
Supplement: S4 Table — Data type, substitution model, tree searching operations, starting tree method and branch support are indicated. (PDF) [file pone.0156199.s005.pdf]

S4 Table. Maximum-likelihood reconstruction tree parameters. Data type, substitution model, tree searching operations, starting tree method and branch support are indicated.

Data	Substitution model	Tree searching operations	Starting tree	Branch support
HMG	Dayhoff	Best of NNI	Neighbor-joining	aLRT (SH-like)
SOXE	LG	Best of NNI and SPR	Neighbor-joining	aLRT (SH-like)
SOX9	HKY+G	Best of NNI and SPR	Neighbor-joining	aLRT (SH-like)

NNI: nearest neighbor interchange, SPR: subtree pruning and regrafting, aLRT: approximate likelihood ratio test
