# Supplementary material for: Dimerization and Transactivation Domains as Candidates for Functional Modulation and Diversity of Sox9
Source: PLoS One. 2016 May 19;11(5):e0156199. doi: 10.1371/journal.pone.0156199 (PMC4873142; doi:10.1371/journal.pone.0156199)
Supplement: S1 Text — Codon based alignment of all sequences used for Sox9 phylogenetic inference. (PDF) [file pone.0156199.s006.pdf]

S1 Text. Sox9 multiple codon sequence alignment. Codon based alignment of all Sox9 sequences used for the phylogenetic inference.

```
            1
HoSapSSDR3  ATGAATCTCCTGGACCCCTTCATGAAGATGACCGACGAGCAGGAGAAG---GGCCTGTCCGGCGCCCCCAGCCCCACC
MuMusSCG1   ATGAATCTCCTGGACCCCTTCATGAAGATGACCGACGAGCAGGAGAAG---GGCCTGTCTGGCGCCCCCAGCCCCACC
CaLupS1     ATGAATCTCCTGGACCCCTTCATGAAGATGACCGACGAGCAGGAGAAG---GGCCTGTCCGGCGCCCCCAGCCCCACC
GaGalSTF2   ATGAATCTCCTAGACCCCTTTATGAAAATGACAGAAGAACAGGATAAA---GGCCTCTCCGGCGCCCCCAGCCCCACC
CoJapSSDRY  ATGAATCTCCTAGACCCCTTCATGAAAATGACGGAAGAACAGGACAAA---TGCATCTCCGACGCCCCCAGCCCCACC
AlMisS1     ATGAATCTCCTAGACCCCTTCATGAAAATGACAGAAGAGCAGGAGAAA---TGCATCTCTGGCGCCCCCAGCCCCACT
TrScrHCC    ATGAATCTCCTTGACCCCTTCATGAAAATGACAGAAGAGCAGGACAAA---TGCATCTCTGGCGCCCCTAGCCCCACC
LeOliS      ATGAATCTCCTTGACCCCTTCATGAAAATGACAGAAGAGCAGGACAAA---TGCATCTCTGGCGCCCCTAGCCCCACC
AnPlaSSDRY  ATGAATCTCCTAGACCCCTTCATGAAAATGACAGAAGAACAGGACAAA---TGTATCTCCGACGCCCCCAGCCCCACC
LaChaTFS9   ATGAATCTACTCGATCCCTACATGAAAATGACAGAAGAGCAGGAGAAG---TGTATTTCTGATGCTCCCAGCCCCACT
XeLaeTFS1   ATGAATCTCTTGGATCCCTTCATGAAGATGACAGAAGAGCAAGATAAG---TGCATGTCCGGGGCTCCCAGCCCAACA
BuBufTFSP   ATGAATCTCTTGGATCCCTTCATGAAGATGACAGAAGAGCAAGATAAG---TGCATGTCCGGGGCCCCCAGCCCAACA
XeSilTFS9   ATGAATCTCTTGGATCCCTTCATGAAGATGACAGAAGAGCAAGATAAG---TGCATGTCCGGGGCCCCCAGCCCAACA
BuGarS      ATGAATCTCTTGGATCCCTTCATGAAGATGACAGAAGAGCAAGACAAG---TGCCTGTCTGGGGCCCCAAGCCCTTCC
RhMarS      ATGAATCTCTTGGATCCCTTCATGAAGATGACAGAAGAGCAAGACAAG---TGCCTGTCTGGGGCCCCTAGCCCTTCC
GlRugSAA    ATGAATCTATTGGATCCTTTCTTAAAGATGACAGAAGAGCAAGAGAAG---TGCCTATCTGGGGCACCCAGCCCTAGC
PlWalS      ATGAATCTCCTAGACCCCTTCATGAAAATGACCGAGGAGCAGGAGAAG---TGCCTGTCTGGAGCCCCCAGCCCCTCC
ScCanS1     ATGAATCTCCTCAACCCCTTCGTCAAAATGACAGAGGGACACGAGAAG---ACGCTCTCCGACGTGCCCAGCCCGAGC
DaRerHBTFS  ATGAATCTCCTCGACCCCTACCTGAAGATGACGGATGAGCAAGAGAAG---TGTCTGTCTGATGCACCCAGTCCGAGC
MoAlbHBTF1  ATGAATCTCCTCGACCCTTACCTGATGATGACGGAGGAACAAGACAAG---TGTCTCTCTGATGCCCCGAGCCCGAGC
OrNilTFS9a  ATGAATCTCCTCGACCCCTACCTGAAGATGACGGAGGAACAAGACAAG---TGTCTCTCTGACGCCCCGAGCCCGAGC
GaAcuTFS9a  ATGAATCTCCTCGACCCTTACCTGAAGATGACGGAGGAACAAGACAAG---TGTCTCTCCGACGCCCCGAGCCCGAGC
XiMacTFS9a  ATGAATCTCCTCGATCCCTACCTGAAGATGACGGAGGAACAAGACAAG---TGTCTCTCTGACGCCCCGAGCCCGAGC
TaRubTFS9b  ATGAATCTCCTCGATCCTTACCTGAAGATGACGGAGGAACAAGATAAG---GGTCTCTCTGACGCCCCGAGCCCGAGC
TeNigTFS9a  ATGAATCTCCTCGATCCTTATCTGAAGATGACGGAGGAACAAGATAAG---GGTATGTCTGACGCCCCGAGTCCGAGC
OrLatSCC1   ATGAATCTCCTCGACCCTTACCTGAAGATGACGGAGGAACAAGACAAA---TGTCTCTCTGACGCCCCGAGCCCGAGC
AsMexTFS9a  ATGAATCTCCTGGACCCCTTCCTGAAGATGACGGACGAGCAAGACAAA---GGTCTGTCCGCCGCGCCGAGCCCCAGC
GaMorS1     ATGAATCTCCTCGACCCTTTCCTGAAGATGACGGAGGAACAAGAAAAG---TGTCTTTCGGACGTCCCGAGTCCGAGC
MoAlbHBTFS  ATGAATCTCCTCGACCCTTACCTGAAGATGACAGAAGAACAGGAGAAG---TGTCACTCTGACGCTCCCAGTCCCAGT
OdBonTFS    ATGAATCTCCTCGATCCTTACCTGAAGATGACAGAAGAACAGGAGAAG---TGTCACTCCGACGCTCCCAGCCCCAGC
PoRetS      ATGAATCTCCTCGACCCTTACCTGAAGATGACCGAAGAACAGGAGAAA---TGTCATTCAGACGCTCCGAGCCCCAGC
XiMacTFS9b  ATGAATCTCCTCGACCCTTACCTGAAGATGACAGAAGAACAGGAGAAA---TGTCATTCAGACGCTCCGAGCCCCAGC
EpCoiS2     ATGAATCTACTCGACCCTTACCTGAAGATGACAGAAGAACAGGAGAAG---TGTCACTCTGACGCTCCGAGTCCCAGC
TaRubTFS9a  ATGAATCTCCTCGACCCCTACCTGAAGATGACAGAAGAACAGGAGAAG---TGTCACTCCGACGCTCCCAGCCCCAGC
TeNigTFS9b  ATGAATCTCCTCGACCCATACTTGAAAATGACAGAAGAACAGGAGAAG---TGTCACTCCGACGCTCCCAGCCCCAGC
OrNilTFS9b  ATGAATCTCCTCGACCCTTACCTGAAGATGACAGAAGAACAGGAGAAG---TGTCACTCTGACGCTCCCAGCCCAAGC
CiMonTFS9   ATGAATCTCCTCGATCCTTACCTGAAGATGACAGAAGAACAGGAGAAG---TGTCACTCTGACGCTCCCAGCCCAAGT
CySemSHB    ATGAATCTCCTCGACCCGTACCTGAAGATGACAGATGAACAGGAGAAG---TGTCACTCTGACGCTCCGAGCCCCTGC
OrLatSCC    ATGAATCTCCTCGATCCATACCTGAAGATGACAGAAGAACAGGAGAAG---TGTCACTCCGACGCTCCCAGTCCCAGC
GaAcuTFS9b  ATGAATCTCCTCGACCCTTACCTGAAGATGACAGAAGAACAGGAGAAG---TGTCACTCTGACGCTCCCAGTCCCAGC
SaSalTFS2   ATGAATCTACTCGACCCCTTCCTGAAGATGACAGACGAACAGGAGAAG---TGTTTCTCTGACGCTCCAAGCCCCAGC
GaMorS2     ATGAATCTACTCGACCCCTACCTTAAAATGACAGAAGAACAGGAGAAGTGTTGTCACTCTGACGTGCCAAGCCCCAGC
AsMexTFS9b  ATGAATCTCCTCGACCCCTACGTGAAGATGACCGACGACCGGGACAAG---TGTCTCCGCGACGCCCCGAGCCCCGGT
ClGarS      ATGAATCTCCTCGAGCCTTACGTGAAGATGACCGACGAGCAGGACAAA---TGTCTCTCCGACGCGCCGAGTCCGAGC
CyCarHTF13  ATGAATCTCTTCGACTCCTACCTGAAAATGAGCGACGAGCAGGACAAG---GGTCTCTCCGACGCGCCCAGCCCGAGC
ClGarS1     ATGAATCTCCTCGACCCCTTCCTGAAGATGACGGACGAGCAAGACAAA---AGTCTGTCGGACGCCCCGAGTCCGAGC
DaRerHBTF1  ATGAATCTCCTCCAGCGCGGGCTGAAGATG---------------------AGTGTGTCCGGAGCTCCGAGCCCGAGT

           79
HoSapSSDR3  ATGTCC---GAGGACTCC---GCGGGCTCGCCC---TGCCCGTCG---GGCTCCGGC------------TCGGACACC
MuMusSCG1   ATGTCG---GAGGACTCG---GCTGGTTCGCCC---TGTCCCTCG---GGCTCCGGC------------TCGGACACG
CaLupS1     ATGTCC---GAGGACTCG---GCGGGCTCGCCC---TGCCCTTCG---GGCTCCGGC------------TCGGACACC
GaGalSTF2   ATGTCG---GATGACTCC---GCGGGGTCCCCC---TGCCCCTCC---GGATCCGGC------------TCGGACACG
CoJapSSDRY  ATGTCG---GATGACTCC---GCCGGGTCTCCT---TGTCCCTCC---GGATCCGGC------------TCGGACACG
AlMisS1     ATGTCG---GATGACTCT---GCAGGCTCTCCC---TGCCCCTCT---GGCTCCGGA------------TCGGACACG
TrScrHCC    ATGTCG---GACGACTCC---GCGGGGTCCCCC---TGCCCTTCT---GGTTCCGGA------------TCGGACACG
LeOliS      ATGTCG---GACGACTCC---GCGGGGTCCCCC---TGCCCTTCT---GGTTCCGGC------------TCGGACACT
AnPlaSSDRY  ATGTCG---GATGACTCC---GCCGGGTCCCCC---TGCCCCTCT---GGATCCGGC------------TCGGACACG
LaChaTFS9   ATGTCG---GAGGATTCT---GCGGGGTCCCCC---TGTCACTCG---GGCTCAGGA------------TCAGACACT
XeLaeTFS1   ATGTCC---GACGACTCG---GCAGGTTCCCCA---TGTCCCTCT---GGCTCCGGC------------TCCGACACG
BuBufTFSP   ATGTCC---GAGGACTCG---GCTGGCTCCCCA---TGCCCCTCC---GGCTCTGGC------------TCCGACACG
XeSilTFS9   ATGTCC---GAGGACTCG---GCTGGCTCCCCA---TGCCCCTCC---GGCTCTGGC------------TCCGACACG
BuGarS      ATGTCT---GAGGACTCT---GCTGGCTCCCCT---TGCCCCTCT---GGCTCAGGC------------TCAGATACT
RhMarS      ATGTCT---GAGGACTCT---GCTGGCTCCCCT---TGCCCCTCT---GGCTCAGGC------------TCAGATACT
GlRugSAA    ATGTCT---GAGGACTCA---GCTGGCTCCCCC---TGCCCCTCT---GGTTCTGGA------------TCAGACACA
```

```
PlWalS       ATGTCC---GAGGACTCG---GTGGGCTCGCCC---TGCCCCTCG---GGCTCCAGC------------TCGGACGCC
ScCanS1      ATGTCCGAGGAGGAATCT---TCCGATTCCCCC---TGCGGTTCC---GGGGCTGGC------------TCCGACGCC
DaRerHBTFS   ATGTCC---GAGGACTCC---GCGGGCTCGCCC---TGCCCGTCC---GCCTCGGGC------------TCAGACACT
MoAlbHBTF1   ATATCC---GAAGACTCC---GCGGGCTCCCCT---TGCCCATCT---GGGTCGGGC------------TCCGACACG
OrNilTFS9a   ATGTCT---GAGGACTCT---GCGGGCTCCCCG---TGCCCGTCC---GGCTCGGGC------------TCCGACACC
GaAcuTFS9a   ATGTCC---GAGGACTCC---GCGGGATCTCCG---TGCCCGTCC---GGCTCGGGC------------TCCGACACA
XiMacTFS9a   ATGTCC---GAGGACTCG---GCGGGTTCCCCG---TGCCCGTCC---GGCTCGGGG------------TCCGACGCC
TaRubTFS9b   ATGTCT---GATGACTCC---GCGGGCTCTCCG---TGCCCGTCC---GGTTCGGGG------------TCAGACACG
TeNigTFS9a   ATGTCC---GAGGACTCC---CCGGGCTCTCCG---TGTACGTCT---GGCTCAGGG------------TCAGACACA
OrLatSCC1    ATGTCC---GAGGACTCC---GCGGGATCCCCGGCCAGCCCGTCC---GGGTCGGGC------------TCCGACACC
AsMexTFS9a   TTGTCC---GAGGACTCC---GCGGGCTCCCCG---TGCCCCTCC---GCCTCCGGA------------TCCGACCCG
GaMorS1      ATGTCA---GAGGATTCT---ACGGGCTCGCCG---TGCCCCTCC---AGCTCGAGC------------TCCGACACC
MoAlbHBTFS   ATGTCT---GAGGACTCC---GCAGGCTCGCCG---TGCCCGTCC---GGGTCCGGT------------TCGGATACC
OdBonTFS     ATGTCC---GAAGACTCC---GCGGGCTCGCCG---TGCCCGTCC---GGCTCCGGT------------TCGGACACT
PoRetS       ATGTCT---GAAGACTCA---GCTGGCTCGCCG---TGCCCGTCC---GGCTCCGGT------------TCGGACACC
XiMacTFS9b   ATGTCT---GAAGACTCA---GCGGGCTCGCCG---TGCCCGTCC---GGCTCCGGT------------TCGGACACC
EpCoiS2      ATGTCT---GAGGACTCC---GCAGGCTCGCCG---TGCCCGTCC---GGGTCCGGT------------TCGGACACT
TaRubTFS9a   ATGTCC---GAGGACTCC---GCGGGCTCTCCG---TGCCCGTCG---GGCTCCGGC------------TCGGACACT
TeNigTFS9b   ATGTCC---GAGGACTCC---GCGGGCTCTCCG---TGCCCGTCC---GGCTCCGGC------------TCGGACACT
OrNilTFS9b   ATGTCT---GAGGACTCC---GCGGGCTCGCCT---TGTCCGTCC---GGGTCGGGT------------TCGGACACT
CiMonTFS9    ATGTCT---GAGGACTCC---GCGGGCTCGCCT---TGTCCGTCC---GGCTCCGGT------------TCGGACACT
CySemSHB     ATGTCT---GACGACTCT---GCCGGCTCGCCC---TGTCCATCA---GGCTCCGGT------------TCGGACACC
OrLatSCC     ATGTCT---GAGGACTCC---GCAGGTTCGCCT---TGTCCGTCC---GGCTCCGGT------------TCGGACACC
GaAcuTFS9b   ATGTCC---GAGGACTCC---GCAGGCTCACCG---TGC---TCC---GTGTCCGGT------------TCGGACACT
SaSalTFS2    ATGTCT---GACGATTCG---GTCGGCTCGCCG---TGCCCGTCC---GGCTCCGGT------------TCCGACACC
GaMorS2      ATGTCTGAAGAAGACTCC---GTTGGCTCTCCT---TGCCCGTCC---GGCTCTGGG------------TCAGACACC
AsMexTFS9b   ATGGATTCCGAGGACTCCGGCGCCGCCTCCCCG---TGCCCGTCC---GGTTCGGGC------------TCGGACACG
ClGarS       ATGTCG---GAAGACTCG---GCAGCCTCCCCG---TGTCCGTCT---GGTTCGGGC------------TCCGACACC
CyCarHTF13   ATGTCC---GAGGATTCC---GCCGGGTCTCCG---TGTCCGTCC---GGATCGGGC------------TCGGACACC
ClGarS1      ATGTCC---GAGGATTCC---GCGGGGTCTCCG---TGTCCCTCC---GCCTCCGGTTCTGGCTCGTGCTCGGACGTG
DaRerHBTF1   CTTTCC---GAGGACTCC---GCCGGCTCTCCG---TGCGCCTCCGCCGGCTCGGGA------------TCGGACAGC

           157
HoSapSSDR3   GAGAAC---------ACGCGG---CCCCAGGAGAACACGTTCCCCAAGGGCGAG---CCC---------GATCTGAAG
MuMusSCG1    GAGAAC---------ACCCGG---CCCCAGGAGAACACCTTCCCCAAGGGCGAG---CCG---------GATCTGAAG
CaLupS1      GAGAAC---------ACGCGG---CCCCAGGAGAACACGTTCCCCAAGGGCGAG---CCG---------GACCTGAAG
GaGalSTF2    GAGAAC---------ACCCGTCCTCCTCAAGAGAACACCTTCCCCAAAGGCGAC---CCG---------GACCTGAAG
CoJapSSDRY   GAGAAC---------ACCCGA---CCTCAAGAAAACACCTTCCCCAAAGGCGAC---CCG---------GACCTGAAG
AlMisS1      GAGAAC---------ACCCGG---CCCCAAGAAAACACCTTCCCCAAGGGGGAC---CCG---------GACCTCAAG
TrScrHCC     GAGAAC---------ACCAGA---CCGCAAGAAAACACTTTCCCCAAGGGCGAT---CCG---------GATCTGAAG
LeOliS       GAGAAC---------ACCAGA---CCGCAAGAAAACACTTTCCCCAAGGGCGAT---CCG---------GATCTGAAG
AnPlaSSDRY   GAGAAC---------ACCAGA---CCCCAAGAAAACACCTTCCCCAAGGGCGAC---CCG---------GACCTGAAG
LaChaTFS9    GAGAAC---------ACCAGA---CCTCAGGAGAACACCTACTCCAATGGGGAC---TTG---------GAGATGAAG
XeLaeTFS1    GAGAAC---------ACCAGA---CCCCAAGAAAACACTTTCCCCAAAGGGGAC---CAG---------GAGCTGAAG
BuBufTFSP    GAGAAC---------ACCAGA---CCCCAAGAGAACACTTTCCCCAAAGGGGAC---CCG---------GAGCTGAAG
XeSilTFS9    GAGAAC---------ACCAGA---CCCCAAGAGAACACTTTCCCCAAAGGGGAC---CCG---------GAGCTGAAG
BuGarS       GAGAAC---------ACCAGA---CCTCAAGAGAACACTTTCCCCAAAGGGGAT---CCT---------GACATGAAG
RhMarS       GAGAAC---------ACCAGA---CCTCAAGAGAACACTTTCCCCAAAGGGGAT---CCT---------GACATGAAG
GlRugSAA     GAGAAC---------ACCAGA---CCCCAAGAGAACACCTTCACCAAGGGGGAC---CAG---------GACCTGAAG
PlWalS       GAGAAC---------ACCCGG---CCCATGGAGAACGGCTTCCCGAAGGGCGAGCTGCAG---------GAGCTGAAG
ScCanS1      GAGAAC---------ACGAAA---CCCGGCGAGAACGGC------CAGGGCGAC---CCGTGCGCCCAGAGCCTGGAT
DaRerHBTFS   GAGAAC---------ACGCGC---CCGGCGGAGAACAGCCTCCTGGCTGCAGACGGGACG---CTCGGAGACTTCAAG
MoAlbHBTF1   GAGAAC---------ACCCGG---CCGTCCGAGAACGGGCTGCTCAGAACGGACGGAACC---CTAAGCGACTTCAAG
OrNilTFS9a   GAGAAC---------ACCCGG---CCGTCGGAGAACGGGCTGCTCAGAGCGGACGGATCT---CTGGGCGACTTCAAA
GaAcuTFS9a   GAGAAC---------ACCCGG---CCATCTGAGAACGGGCTACTCGGTCTAGAT------------GGAGAGTTTAAG
XiMacTFS9a   GAGAAC---------ACGCGG---CCGTCGGAGAACGCGCTGCGGCGCGCGGACGCGGCGCTGCTGGGCGACTTCAAG
TaRubTFS9b   GAGAAC---------ACCCGG---CCGTCGGAGAACGGGCTGATGCGGGCGGAC------------GGAGACCTAAAG
TeNigTFS9a   GAGAAC---------ACCCGG---CCGTCAGAGAACGGGCTGCTGCGGGCGGAC------------GGAGACCTAAAG
OrLatSCC1    GAGAAC---------ACCCGG---CCGCGGGAAAACGGCCTGATGCGCGCGGACGGAGCT---CTGAGCGACTTCAAG
AsMexTFS9a   GACCGC---------GCCGCC---GCCGAGAGCCGC---------------------------CTGCACGACTTCAAG
GaMorS1      GAGAAC---------ACGAGA---CCCTCGGAGAACGGCTTCCTGCGCGCGGACGGGGTCACCATCGGCGACTTCAAG
MoAlbHBTFS   GAGAAC---------ACCCGA---CCGTCAGACAACCACCTCCTCAGGGGTCCGGAC---------------TACAAG
OdBonTFS     GAAAAT---------ACCCGA---CCGTCTGACAACCACCTCCTTGGAGGTCCAGAC---------------TACAAG
PoRetS       GAGAAC---------ACCCGG---CCGTCCGACAACCACCTCCTCAGGGGTCCGGAC---------------TACAAG
XiMacTFS9b   GAGAAC---------ACCCGG---CCGTCCGACAACCACCTCCTCAGGGGACCGGAC---------------TACAAG
EpCoiS2      GAGAAC---------ACCCGG---CCGTCCGACAACCACCTTCTCCTAGGTCAAGAC---------------TACAAG
TaRubTFS9a   GAGAAC---------ACCAGA---CCGTCCGACAACCACCTCCTTCTGGGTCCAGAC---------------TACAAG
TeNigTFS9b   GAGAAC---------ACCAGG---CCGTCCGACAACCACCTCCTTCTGGGTCCAGAC---------------TACAAG
```

```
OrNilTFS9b  GAAAAC---------ACTCGT---CCGTCCGACAACCACCTCCTCATGGGTCAAGAC---------------TATAAG
CiMonTFS9   GAAAAC---------ACTCGT---CCGTCCGACAACCACCTCCTCATGGGTCCAGAC---------------TATAAG
CySemSHB    GAGAAC---------ACCCGT---CCCACCGACAACCACCTCCTCGGGGGTCCTGAC---------------TACAAG
OrLatSCC    GAAAAC---------ACCCGG---CCGTCCGACAACCATCTCATCAGGGGGCCGGAC---------------TACAAG
GaAcuTFS9b  GAGAAC---------ACCCGG---CCGTCCGACAACCACCTCCTCCTGGGTGCAGAC---------------TACAAG
SaSalTFS2   GAGAAT---------ACCAGG---CCGTCCGATAATCATCTCTTACTGGGTCCAGATGGCGTGCTCGGCGAATTCAAG
GaMorS2     GAGAAC---------CACCGG---CCGTCCGACAACCACCTCCTCTTAGGACAGGACCATCCTATGGGTGAATACAAG
AsMexTFS9b  GAAAAC---------ACGCGG---CCAACCGACATCCAC------------GACGAT---------------TTTAAG
ClGarS      GAGAAC---------ACGCGGCCGCCGTCCGAGAATCGG---------------------------------TTCAAG
CyCarHTF13  GAGAAC---------ACCCGC---CCGGAGGAGCAC---------------------------CTGGGAGAGTTTAAG
ClGarS1     GAGACCGGCGGTGGCGTGCGC---GCGCGCGACGTCGGC---------------------------GGCGAGCTGAAA
DaRerHBTF1  GAGACC------------------CCCCGCGCGGAGCCGCCGCTG---------------------------------

          235
HoSapSSDR3  AAG---------------GAGAGCGAGGAGGAC---AAGTTCCCCGTGTGCATCCGCGAGGCGGTCAGCCAGGTGCTC
MuMusSCG1   AAG---------------GAGAGCGAGGAAGAT---AAGTTCCCCGTGTGCATCCGCGAGGCGGTCAGCCAGGTGCTG
CaLupS1     AAG---------------GAGAGCGAGGAGGAC---AAGTTCCCCGTGTGCATCCGCGAGGCCGTCAGCCAGGTGCTC
GaGalSTF2   AAG---------------GAAAGCGACGAGGAC---AAATTCCCCGTGTGCATCCGCGAGGCCGTGAGCCAGGTGCTC
CoJapSSDRY  AAG---------------GAGAACGACGAGGAC---AAATTCCCCGTGTGCATCCGCGAAGCTGTGAGCCAGGTGCTC
AlMisS1     AAA---------------GACAGCGACGAGGAC---AAGTTCCCCGTTTGCATCCGGGAGGCTGTCAGCCAGGTCCTC
TrScrHCC    AAA---------------GAGAGCGACGAGGAC---AAATTCCCGGTGTGCATCCGAGAGGCGGTCAGCCAAGTCCTG
LeOliS      AAA---------------GAGAGCGACGAGGAC---AAATTCCCGGTGTGCATCCGAGAGGCGGTCAGCCAAGTCCTG
AnPlaSSDRY  AAG---------------GAGAGCGACGAGGAC---AAGTTCCCGGTGTGCATCCGAGAGGCGGTGAGCCAAGTGCTC
LaChaTFS9   AAA---------------GAGACAGACGAAGAC---AAATTCCCAGTCTGCATCCGCGAAGCTGTCAGCCAGGTCCTG
XeLaeTFS1   AAG---------------GAGACGGAGGATGAG---AAGTTCCCCGTGTGCATCAGAGAAGCGGTCAGCCAGGTGTTG
BuBufTFSP   AAG---------------GAGACAGAGGACGAA---AAGTTTCCTGTGTGCATCAGAGAGGCGGTCAGCCAGGTGCTG
XeSilTFS9   AAG---------------GAGACAGAGGACGAA---AAGTTTCCTGTGTGCATCAGAGAGGCGGTCAGCCAGGTGCTG
BuGarS      AAG---------------GAAACAGAGGATGAA---AAGTTCCCTGTCTGCATCAGAGAGGCGGTCAGCCAGGTGCTG
RhMarS      AAG---------------GAAACAGAGGATGAA---AAGTTCCCTGTCTGCATCAGAGAGGCGGTCAGCCAGGTGCTG
GlRugSAA    AAG---------------GAGACAGAGGATGAG---AAGTTCCCTGTCTGCATCAGAGAGGCGGTCAGCCAGGTGCTG
PlWalS      AAG---------------GAGAACGAGGAAGAC---AAGTTCCCCGTCTGCATCCGCGAGGCGGTCAGCCAGGTGCTG
ScCanS1     TCC---------------AAAAAGGACGACGAC---AAGTTCCCGGCGTGTATCCGCGAGGCGGTGACCCAGGTGCTG
DaRerHBTFS  AAG---------------GACGAA---GAGGAC---AAGTTCCCGGTGTGCATCCGAGAGGCGGTGTCCCAGGTGCTG
MoAlbHBTF1  AAG---------------GACGAG---GACGAT---AAGTTTCCCGCATGTATCCGCGAAGCTGTGTCCCAGGTGCTC
OrNilTFS9a  AAG---------------GACGAG---GAAGAC---AAGTTTCCCGCTTGCATCCGCGAAGCCGTGTCCCAGGTGCTC
GaAcuTFS9a  AAG---------------GATGAG---GACGAT---AAATTTCCCGCTTGCATCCGCGAGGCAGTGTCCCAGGTGCTC
XiMacTFS9a  AAG---------------GACGAG---GACGAT---AAGTTCCCCGCGTGCATCCGCGAGGCCGTGTCCCAGGTGCTG
TaRubTFS9b  AAG---------------GATGAG---GAAGAC---AAGTTTCCAGCGTGCATTCGCGATGCCGTGTCCCAGGTCCTC
TeNigTFS9a  AAA---------------GACGAG---GAGGAC---AAGTTCCCAGCGTGCATCCGCGACGCCGTGTCCCAGGTCCTA
OrLatSCC1   AAG---------------GACGAA---GACGAC---AAGTTTCCCGCGTGCATCCGGGAGGCGGTGTCTCAGGTGCTG
AsMexTFS9a  AAG---------------GACGAGGTGGACGAC---AAGTTCCCCGTGTGCATCCGCGAGGCCGTCACGCAGGTGCTC
GaMorS1     AAG---------------GACGAG---GAGGAT---AAGTTTCCCGTGTGCATCCGCGAGGCCGTGTCCCAGGTGCTC
MoAlbHBTFS  AAG---------------GAGGGCGAAGAAGAA---AAGTTCCCCGTGTGTATCAGAGATGCAGTGTCCCAGGTATTG
OdBonTFS    AAG---------------GAGGGCGAAGAAGAA---AAGTTCCCCGTGTGTATCAGGGATGCGGTGTCCCAGGTGTTA
PoRetS      AAG---------------GAGGGCGAGGAAGAG---AAGTTCCCGGTGTGCATCAGAGACGCGGTGTCCCAGGTGCTG
XiMacTFS9b  AAG---------------GAGGGCGAGGAAGAG---AAGTTCCCGGTGTGCATAAGAGATGCGGTGTCCCAGGTGCTG
EpCoiS2     AAG---------------GAGGGCGAAGAAGAG---AAGTTCCCCGTGTGTATCAGAGATGCCGTGTCCCAGGTGTTG
TaRubTFS9a  AAG---------------GAGAACGAAGAAGAG---AAGTTCCCCGTGTGCATCAGGGATGCGGTGTCCCAGGTGCTG
TeNigTFS9b  AAG---------------GAAAACGAAGAAGAG---AAGTTCCCCGTGTGTATCAGAGATGCGGTGTCCCAAGTGCTG
OrNilTFS9b  AAG---------------GAAACCGAAGAGGAA---AAGTTTCCCGTGTGTATCAGAGACGCGGTGTCTCAGGTGTTG
CiMonTFS9   AAG---------------GAAAGCGAAGAGGAG---AAGTTCCCCGTGTGTATCAGAGATGCGGTGTCTCAGGTGTTG
CySemSHB    AAA---------------GAGGGCGAAGAGGAG---AAGTTTCCTGTCTGCATCAGAGATGCAGTGTCCCAGGTGCTG
OrLatSCC    AAA---------------GAGGGTGAAGAAGAG---AGGTTCCCCGTGTGCATTAGAGACGCGGTGTCCCAAGTGCTC
GaAcuTFS9b  AAA---------------GAGGGCGAAGAAGAA---AAGTTCCCCGTGTGCATCAGAGATGCGGTGTCCCAGGTGTTG
SaSalTFS2   AAG------------------GCTGACCAAGAC---AAGTTCCCAGTATGTATCAGAGATGCGGTGTCTCAGGTGCTG
GaMorS2     AAG---------------GAGGCGGAGGAAGAG---AAGTTCCCCGTGTGCATTAGAGATGCCGTGTCCCAGGTGCTG
AsMexTFS9b  AAG---------------GAC---GATGAGGAA---AAGTTCCCCGTCTGCATCCGGGACGCAGTGTCGCAGGTGCTG
ClGarS      AAA---------------GACGAAGAGGATGAAAACAAGTTCCCCGTCTGCATCCGCGACGCCGTGTCACAGGTGCTC
CyCarHTF13  AAG---------------GACGAGGAC---------AAGTTCCCCGTGTGTATCAGGGACGCGGTGTCTCAGGTGCTG
ClGarS1     AAGTTGGACGGGGAGGACGAGCACGAGAACAACGAGAAGTTCCCGCAGTGCATCCGCGAGGCTGTGTCGCAGGTGCTC
DaRerHBTF1  ------------------CACCGGGACGAGCAGGAGAAGTTCCCGGTGTGTATCCGGGACGCGGTGTCGCAGGTGCTG

          313
HoSapSSDR3  AAAGGCTACGACTGGACGCTGGTGCCCATGCCGGTGCGCGTCAACGGCTCCAGCAAGAACAAGCCGCACGTCAAGCGG
MuMusSCG1   AAGGGCTACGACTGGACGCTGGTGCCCATGCCCGTGCGCGTCAACGGCTCCAGCAAGAACAAGCCACACGTCAAGCGA
CaLupS1     AAGGGCTACGACTGGACGCTGGTGCCCATGCCCGTGCGCGTCAACGGCTCGAGCAAGAACAAGCCGCACGTCAAGCGG
GaGalSTF2   AAGGGCTACGACTGGACCCTGGTGCCCATGCCCGTGCGGGTGAACGGATCCAGCAAGAACAAACCCCACGTGAAGCGC
CoJapSSDRY  AAGGGCTACGATTGGACCCTGGTGCCCATGCCCGTGCGGGTGAATGGATCCAGCAAGAACAAACCCCACGTGAAGCGT
```

```
AlMisS1      AAGGGCTACGACTGGACCCTGGTCCCCATGCCGGTCCGGGTGAATGGATCCAGTAAGAACAAGCCGCACGTGAAGAGA
TrScrHCC     AAGGGTTACGACTGGACCCTGGTCCCCATGCCGGTCCGTGTGAACGGATCCAGCAAAAACAAGCCCCACGTGAAGAGA
LeOliS       AAGGGTTACGACTGGACCCTGGTCCCCATGCCGGTCCGTGTGAACGGATCCAGCAAAAACAAGCCCCACGTGAAGAGA
AnPlaSSDRY   AAGGGCTACGACTGGACCCTGGTGCCCATGCCGGTCCGGGTGAACGGATCCAGCAAAAACAAGCCCCACGTGAAGAGA
LaChaTFS9    AAGGGATATGACTGGACCCTGGTGCCTATGCCAGTCAGGGTGAATGGATCCAGCAAAAATAAGCCTCATGTCAAGAGA
XeLaeTFS1    AAGGGATATGATTGGACCCTGGTACCGATGCCAGTCAGAGTTAATGGATCCAGCAAGAACAAGCCCCATGTCAAGAGA
BuBufTFSP    AAGGGATATGACTGGACCCTGGTACCGATGCCAGTCAGAGTTAATGGATCCAGCAAGAGCAAGCCTCATGTCAAGAGA
XeSilTFS9    AAGGGATATGACTGGACCCTGGTACCGATGCCAGTCAGAGTTAATGGATCCAGCAAGAGCAAGCCTCATGTCAAGAGA
BuGarS       AAGGGATATGACTGGACCCTGGTGCCCATGCCAGTCAGGGTGAATGGATCCAGCAAGAACAAACCCCACGTCAAGAGG
RhMarS       AAGGGATATGACTGGACCCTGGTGCCCATGCCAGTCAGGGTGAATGGATCCAGCAAGAACAAACCCCATGTCAAGAGG
GlRugSAA     AAGGGATATGACTGGACCCTGGTGCCCATGCCAGTCAGGGTCAATGGATCCAGCAAGAACAAACCGCATGTCAAGAGG
PlWalS       AAGGGCTACGACTGGACCCTGGTGCCCATGCCCGTGCGGGTGAACGGCTCCAGCAAGAACAAGCCGCACGTCAAGCGG
ScCanS1      AAAGGATACGACTGGACCCTGGTGCCCATGCCGGTCCGCGTCAATGGCTCGTCCAAGAACAAACCTCACGTCAAAAGA
DaRerHBTFS   AAGGGTTACGACTGGACGCTGGTGCCCATGCCGGTGAGGGTGAACGGGTCCAGCAAAAACAAGCCGCACGTCAAGAGA
MoAlbHBTF1   AAGGGCTACGACTGGACCCTCGTGCCTATGCCGGTGCGCGTTAACGGATCTACTAAGAACAAGCCTCACGTGAAGAGA
OrNilTFS9a   AAGGGTTACGACTGGACCCTCGTGCCCATGCCGGTGCGCGTTAACGGATCTACCAAGAATAAGCCCCACGTTAAGAGA
GaAcuTFS9a   AAGGGCTACGACTGGACTCTTGTGCCTATGCCAGTGCGCGTTAACGGGTCCTCTAAGAACAAGCCTCACGTGAAAAGA
XiMacTFS9a   AAGGGCTACGACTGGACCCTCGTGCCGATGCCCGTGCGCGTGAACGGATCTACCAAGAACAAGCCGCACGTGAAGAGG
TaRubTFS9b   AAAGGCTACGACTGGACCCTCGTCCCTATGCCAGTGCGCGTAAATGGATCTTCCAAAAACAAACCTCACGTCAAGAGA
TeNigTFS9a   AAAGGCTACGACTGGACCCTCGTCCCCATGCCAGTGCGCGTAAATGGATCTTCCAAAAACAAACCTCACGTCAAAAGA
OrLatSCC1    AAGGGCTACGACTGGACGCTCGTGCCGATGCCGGTGCGCGTAAACGGATCTACAAAGAACAAGCCGCACGTGAAGAGA
AsMexTFS9a   AAGGGCTACGACTGGACGCTCGTGCCCATGCCGGTGCGCGTGAACGGCTCGAGCAAGAACAAGCCGCATGTCAAGAGG
GaMorS1      AAGGGCTACGACTGGACCCTGGTGCCCATGCCCGTGCGCGTAAACGGATCTAGTAAGAACAAGCCGCACGTGAAGAGA
MoAlbHBTFS   AAAGGCTACGACTGGACGTTGGTGCCCATGCCGGTGCGCGTCAACGGCTCAACTAAAAACAAACCTCATGTCAAAAGA
OdBonTFS     AAGGGTTACGACTGGACTCTTGTGCCCATGCCGGTGCGCGTCAACGGCTCAAGCAAAAGCAAACCTCACGTCAAAAGA
PoRetS       AAGGGCTATGACTGGACGCTCGTACCCATGCCAGTGCGCGTCAACGGCTCCAGCAAGAGCAAGCCGCACGTTAAGAGA
XiMacTFS9b   AAGGGCTATGACTGGACTCTAGTACCCATGCCGGTGCGCGTCAACGGCTCCAGCAAGAGCAAACCACACGTTAAGAGA
EpCoiS2      AAGGGCTACGACTGGACGCTGGTGCCCATGCCGGTGCGCGTTAACGGCTCCAGTAAAAGCAAACCTCACGTCAAAAGA
TaRubTFS9a   AAGGGCTACGACTGGACGCTGGTGCCCATGCCCGTGCGCGTCAACGGCTCCAACAAAAATAAACCTCACGTCAAGAGA
TeNigTFS9b   AAGGGTTACGACTGGACGCTGGTGCCGATGCCGGTGCGCGTCAACGGCTCCAACAAAAATAAACCTCACGTGAAAAGA
OrNilTFS9b   AAGGGTTACGACTGGACGCTCGTGCCTATGCCGGTGCGCGTCAACGGCTCGAGTAAAAGCAAACCCCACGTCAAAAGA
CiMonTFS9    AAGGGTTACGACTGGACGCTCGTCCCTATGCCGGTGCGTGTCAACGGCTCGAGTAAAAGTAAACCCCACGTCAAAAGA
CySemSHB     AAGGGATACGACTGGACTCTGGTGCCGATGCCAGTGCGCGTCAACGGCTCGAGTAAAAACAAACCTCATGTGAAAAGA
OrLatSCC     AAGGGCTACGACTGGACTCTGGTGCCGATGCCAGTACGCGTCAACGGCTCGAGTAAAAGCAAACCTCACGTCAAAAGG
GaAcuTFS9b   AAGGGCTACGACTGGACTCTGGTGCCCATGCCGGTGCGCGTCAACGGCTCGAGTAAAAGCAAACCTCACGTCAAGAGA
SaSalTFS2    AAGGGTTACGACTGGACCTTGGTGCCCATGCCCGTCCGAGTGAACGGCTCCAGCAAAAACAAGCCCCATGTCAAGAGA
GaMorS2      AAGGGCTACGACTGGACCCTGGTGCCCATGCCGGTGCGCGTAAACGGTTCCAGTAAAAATAAACCCCACGTGAAGAGA
AsMexTFS9b   AAGGGCTACGACTGGACGCTCGTGCCCATGCCGGTGCGCGTTAACGGCTCGAGCAAGAACAAGCCGCACGTGAAGAGA
ClGarS       AAAGGCTACGACTGGACGCTCGTGCCCATGCCGGTGCGCGTGAACGGCTCGGGGAAGAGCAAGCCGCACGTGAAGAGG
CyCarHTF13   AAGGGCTACGACTGGACCCTGGTGCCCATGCCTGTGCGAGTGAACGGCGCGCACAAGAGCAAGCCGCACGTCAAGAGA
ClGarS1      AAGGGCTACGACTGGACGCTCGTGCCTGTGCCGGTGCGCGTGCACGGCGCCAGCAAAAACAAGCCGCACGTGAAGCGG
DaRerHBTF1   AAGGGCTACGACTGGTCTCTGGTGCCCATGCCGGTGCGGGTGAGCGGCTCCGGGAAGAGCAAACCGCACGTCAAGAGA

          391
HoSapSSDR3   CCCATGAACGCCTTCATGGTGTGGGCGCAGGCGGCGCGCAGGAAGCTCGCGGACCAGTACCCGCACTTGCACAACGCC
MuMusSCG1    CCCATGAACGCCTTCATGGTGTGGGCGCAGGCTGCGCGCAGGAAGCTGGCAGACCAGTACCCGCATCTGCACAACGCG
CaLupS1      CCCATGAACGCCTTCATGGTGTGGGCGCAGGCGGCGCGCAGGAAGCTCGCCGACCAGTACCCGCACCTGCACAACGCC
GaGalSTF2    CCCATGAACGCCTTCATGGTGTGGGCCCAGGCGGCTCGAAGGAAGCTGGCTGACCAGTACCCGCATCTGCACAACGCC
CoJapSSDRY   CCCATGAACGCCTTTATGGTGTGGGCCCAGGCGGCTCGAAGGAAACTGGCTGACCAATACCCGCATCTGCACAACGCC
AlMisS1      CCCATGAATGCCTTCATGGTGTGGGCTCAGGCGGCCCGGAGAAAGCTGGCTGACCAGTACCCCCATCTGCACAACGCC
TrScrHCC     CCCATGAACGCCTTCATGGTGTGGGCACAGGCGGCCCGGAGGAAGCTGGCGGACCAGTATCCGCATCTGCACAACGCA
LeOliS       CCCATGAACGCCTTCATGGTGTGGGCACAGGCGGCCCGGAGGAAGCTGGCGGACCAGTATCCGCATCTGCACAACGCA
AnPlaSSDRY   CCCATGAACGCCTTCATGGTGTGGGCCCAGGCGGCCCGGAGGAAGCTGGCTGACCAGTACCCGCATCTGCACAACGCG
LaChaTFS9    CCAATGAATGCCTTTATGGTCTGGGCTCAGGCTGCAAGAAGGAAACTGGCTGATCAGTACCCTCATCTCCATAATGCA
XeLaeTFS1    CCAATGAACGCCTTCATGGTCTGGGCACAGGCTGCAAGGAGGAAGCTGGCTGATCAGTACCCCCATCTGCACAATGCA
BuBufTFSP    CCCATGAACGCCTTCATGGTGTGGGCACAGGCTGCAAGGAGGAAGCTGGCCGACCAATACCCCCATCTGCACAATGCA
XeSilTFS9    CCCATGAACGCCTTCATGGTGTGGGCACAGGCTGCAAGGAGGAAGCTGGCCGACCAATACCCCCATCTGCACAATGCA
BuGarS       CCAATGAATGCCTTCATGGTGTGGGCACAGGCTGCCAGGAGGAAGCTTGCGGATCAGTACCCACATCTTCACAATGCA
RhMarS       CCAATGAATGCCTTCATGGTGTGGGCACAGGCTGCCAGGAGGAAGCTTGCGGATCAGTACCCACATCTTCACAATGCA
GlRugSAA     CCCATGAATGCCTTCATGGTGTGGGCACAGGCTGCCCGGAGGAAGCTGGCAGACCAGTATCCCCATCTGCACAATGCG
PlWalS       CCCATGAACGCCTTCATGGTGTGGGCGCAGGCGGCGCGCAGGAAGCTGGCCGACCAGTACCCGCACCTGCACAACGCC
ScCanS1      CCCATGAACGCCTTCATGGTGTGGGCGCAAGCGGCACGGAGGAAGCTGGCCGACCAGTACCCCCATCTGCATAACGCC
DaRerHBTFS   CCGATGAACGCGTTTATGGTGTGGGCGCAGGCGGCGCGCAGGAAACTGGCCGACCAGTACCCGCACCTCCACAACGCG
MoAlbHBTF1   CCCATGAATGCCTTCATGGTGTGGGCTCAGGCTGCTCGGAGGAAACTGGCAGATCAGTACCCACACCTGCATAACGCG
OrNilTFS9a   CCAATGAACGCGTTCATGGTGTGGGCTCAGGCTGCTCGGAGGAAGCTGGCGGATCAGTACCCGCACCTTCACAACGCG
GaAcuTFS9a   CCGATGAATGCTTTCATGGTGTGGGCTCAGGCTGCGCGCAGGAAGCTGGCTGATCAGTATCCTCACCTGCACAACGCA
XiMacTFS9a   CCGATGAACGCCTTCATGGTGTGGGCGCAGGCGGCGCGGAGGAAGCTGGCGGACCAGTACCCGCACCTCCACAACGCG
TaRubTFS9b   CCGATGAACGCTTTCATGGTGTGGGCCCAGGCTGCACGGAGGAAGCTGGCGGATCAATACCCCCACTTGCATAACGCA
```

```
TeNigTFS9a  CCGATGAACGCGTTCATGGTGTGGGCCCAGGCCGCCCGGAGGAAGCTGGCGGATCAGTACCCACACTTGCATAACGCG
OrLatSCC1   CCAATGAACGCCTTCATGGTGTGGGCGCAGGCGGCGCGCAGGAAGCTCGCGGATCAGTACCCCCACCTGCACAACGCG
AsMexTFS9a  CCCATGAACGCGTTCATGGTGTGGGCGCAGGCCGCGCGCAGGAAACTGGCCGACCAGTACCCCCATCTCCACAACGCC
GaMorS1     CCAATGAACGCGTTCATGGTGTGGGCGCAGGCAGCGCGCAGGAAGCTCGCGGACCAGTACCCACATCTCCACAACGCG
MoAlbHBTFS  CCCATGAACGCCTTCATGGTGTGGGCTCAGGCTGCACGGAGGAAGCTGGCTGATCAATACCCACATCTGCACAACGCG
OdBonTFS    CCCATGAACGCATTTATGGTGTGGGCTCAAGCAGCACGAAGGAAACTGGCCGATCAATACCCGCATCTGCACAACGCA
PoRetS      CCCATGAACGCCTTCATGGTTTGGGCTCAGGCGGCGCGGAGGAAGCTGGCCGATCAGTATCCGCACTTGCACAACGCA
XiMacTFS9b  CCCATGAACGCCTTCATGGTTTGGGCTCAGGCGGCGCGGAGGAAGCTGGCCGATCAGTATCCGCATTTGCACAACGCA
EpCoiS2     CCCATGAACGCATTCATGGTGTGGGCTCAAGCTGCACGACGGAAGCTGGCCGATCAATACCCGCATCTGCACAACGCG
TaRubTFS9a  CCCATGAACGCGTTCATGGTGTGGGCCCAGGCTGCACGGAGGAAGCTGGCCGATCAGTACCCGCATCTGCACAACGCG
TeNigTFS9b  CCCATGAACGCGTTTATGGTGTGGGCCCAGGCTGCGCGGAGGAAGCTGGCGGACCAGTACCCGCATCTGCACAACGCG
OrNilTFS9b  CCCATGAACGCGTTCATGGTGTGGGCTCAAGCTGCACGGAGAAAGCTGGCCGATCAATACCCACACCTGCACAACGCA
CiMonTFS9   CCCATGAACGCATTCATGGTGTGGGCTCAAGCTGCACGGAGAAAGCTGGCCGATCAATATCCACATCTGCACAACGCA
CySemSHB    CCCATGAACGCCTTCATGGTTTGGGCACAAGCCGCACGCAGGAAACTGGCCGATCAATACCCGCATCTGCACAACGCA
OrLatSCC    CCCATGAATGCATTCATGGTCTGGGCTCAGGCAGCACGGAGGAAACTGGCCGACCAATACCCGCATTTGCACAACGCA
GaAcuTFS9b  CCCATGAATGCGTTCATGGTCTGGGCTCAAGCCGCAAGGAGGAAGCTGGCCGATCAATACCCGCATTTGCACAATGCC
SaSalTFS2   CCCATGAACGCTTTCATGGTGTGGGCTCAAGCCGCCCGGAGGAAACTGGCGGACCAGTACCCACATCTCCACAATGCA
GaMorS2     CCGATGAACGCCTTCATGGTGTGGGCTCAGGCTGCACGGAGGAAGCTGGCGGACCAGTATCCACATCTCCACAACGCC
AsMexTFS9b  CCGATGAACGCGTTTATGGTGTGGGCGCAGGCGGCGCGGAGGAAGCTTGCGGACCAGTACCCGCATCTGCACAACGCG
ClGarS      CCAATGAACGCGTTTATGGTGTGGGCGCAGGCGGCGCGCAGGAAACTGGCCGATCAATACCCTCACCTTCATAACGCC
CyCarHTF13  CCCATGAACGCGTTTATGGTTTGGGCTCAAGCGGCGCGCAGGAAACTGGCGGACCAGTATCCACACCTGCACAACGCC
ClGarS1     CCAATGAACGCGTTCATGGTGTGGGCCCAGGCCGCGCGCCGCAAGCTCGCAGACCAGTACCCTCACCTGCACAACGCC
DaRerHBTF1  CCCATGAACGCGTTTATGGTTTGGGCTCAAGCCGCGCGGAGGAAACTGGCGGACCAGTACCCGCACCTGCACAACGCG

          469
HoSapSSDR3  GAGCTCAGCAAGACGCTGGGCAAGCTCTGGAGACTTCTGAACGAGAGCGAGAAGCGGCCCTTCGTGGAGGAGGCGGAG
MuMusSCG1   GAGCTCAGCAAGACTCTGGGCAAGCTCTGGAGGCTGCTGAACGAGAGCGAGAAGAGACCCTTCGTGGAGGAGGCGGAG
CaLupS1     GAGCTCAGCAAGACGCTGGGCAAGCTCTGGAGGCTGCTGAACGAGAGCGAGAAGCGGCCCTTCGTGGAGGAGGCGGAG
GaGalSTF2   GAGCTCAGCAAGACGCTGGGCAAGCTGTGGAGGCTGCTGAATGAGAGCGAGAAGCGTCCCTTCGTGGAGGAGGCCGAG
CoJapSSDRY  GAGCTCAGCAAAACGCTGGGCAAGCTGTGGAGGCTGTTGAATGAGAGCGAGAAGCGTCCCTTCGTGGAAGAGGCTGAG
AlMisS1     GAGCTGAGCAAAACCCTGGGCAAGCTCTGGAGGCTGCTGAACGAAAGCGAGAAGCGCCCATTCGTGGAGGAAGCCGAG
TrScrHCC    GAGCTCAGCAAAACCCTGGGCAAACTCTGGAGGTTGCTGAATGAGAGCGAGAAGCGCCCTTTCGTGGAGGAGGCCGAG
LeOliS      GAGCTCAGCAAAACGCTGGGCAAACTCTGGAGGTTGCTGAATGAAAGCGAGAAGCGCCCTTTCGTGGAGGAGGCCGAG
AnPlaSSDRY  GAGCTCAGCAAGACCCTGGGCAAGCTCTGGAGGCTGCTGAATGAGAGCGAGAAGCGTCCCTTCGTGGAGGAGGCCGAG
LaChaTFS9   GAACTCAGCAAAACTTTGGGAAAACTTTGGAGGTTGCTCAATGAGAGCGAAAAGCGCCCGTTTGTAGAAGAGGCTGAG
XeLaeTFS1   GAACTCAGCAAGACGCTGGGAAAGTTATGGAGACTTCTGAATGAGGGTGAGAAACGCCCTTTCGTGGAGGAAGCAGAG
BuBufTFSP   GAACTCAGCAAGACTCTGGGCAAGTTATGGAGACTTCTGAATGAGGGCGAGAAACGCCCTTTCGTGGAGGAAGCAGAG
XeSilTFS9   GAACTCAGCAAGACTCTGGGCAAGTTATGGAGACTTCTGAATGAGGGCGAGAAACGCCCTTTCGTGGAGGAAGCAGAG
BuGarS      GAACTCAGCAAGACCCTGGGCAAGCTCTGGAGGCTGCTGAATGAGAGCGAGAAGCGTCCTTTTGTTGAAGAAGCTGAG
RhMarS      GAACTCAGCAAGACCCTGGGCAAGCTCTGGAGGCTGCTGAATGAGAGCGAGAAGCGTCCTTTTGTAGAAGAAGCTGAG
GlRugSAA    GAGCTCAGCAAGACCCTGGGCAAGCTGTGGAGGCTGCTGAATGAAACCGAGAAGCGCCCTTTTGTGGAGGAAGCGGAG
PlWalS      GAGCTCAGCAAGACACTGGGCAAGCTCTGGAGGCTGCTGAATGAGGGCGAGAAGCGCCCCTTTGTGGAGGAAGCAGAG
ScCanS1     GAGCTAAGCAAAACCCTGGGCAAACTCTGGAGACTGCTGAACGAGGGCGAAAAGCGCCCTTTCGTGGAGGAAACCGAG
DaRerHBTFS  GAGCTCAGCAAAACTCTGGGAAAACTTTGGAGATTACTGAACGAGGTGGAAAAGCGTCCCTTCGTGGAGGAGGCAGAG
MoAlbHBTF1  GAGCTCAGCAAAACTCTGGGGAAACTGTGGAGGCTTCTCAATGAAGGCGAGAAACGGCCGTTTGTGGAAGAAGCTGAG
OrNilTFS9a  GAGCTCAGCAAGACACTCGGAAAACTCTGGAGACTTCTCAATGAAGGAGAGAAGCGGCCGTTCGTGGAGGAGGCTGAG
GaAcuTFS9a  GAGCTCAGCAAAACTCTGGGGAAACTCTGGAGACTTCTGAATGAAGGGGAGAAGCGGCCGTTTGTGGAGGAAGCCGAA
XiMacTFS9a  GAGCTGAGCAAGACGCTGGGCAAGCTGTGGAGACTTCTCAACGAAGGCGAGAAGCGGCCGTTTGTGGAAGAGGCCGAG
TaRubTFS9b  GAGCTCAGCAAGACACTGGGGAAACTCTGGAGACTTTTGAATGAAGTCGAGAAGCGGCCGTTTGTGGAAGAGGCTGAG
TeNigTFS9a  GAGCTCAGCAAGACGCTCGGGAAACTCTGGAGACTGTTAAATGAAGTCGAGAAGCGGCCGTTTGTGGAAGAGGCAGAG
OrLatSCC1   GAGCTCAGCAAAACTCTGGGGAAGCTGTGGAGACTCCTCAATGAGGGGGAGAAGCGGCCGTTCGTGGAGGAGGCGGAG
AsMexTFS9a  GAGCTCAGCAAAACCCTGGGCAAGCTGTGGAGGCTTCTGAACGATGCAGAGAAGCGTCCCTTTGTGGAGGAGGCGGAA
GaMorS1     GAGCTCAGCAAGACCCTCGGGAAACTGTGGAGACTCCTCAACGAAGGCGAGAAAAGGCCGTTTGTGGAGGAGGCTGAG
MoAlbHBTFS  GAACTCAGCAAAACACTGGGCAAACTTTGGAGATTGCTCAATGAGGTAGAAAAGCGTCCGTTTGTGGAAGAAGCAGAG
OdBonTFS    GAGCTCAGCAAAACCTTGGGGAAACTTTGGAGATTGCTCAATGAGGTAGAGAAGCGCCCGTTTGTGGAGGAAGCTGAG
PoRetS      GAACTCAGCAAAACCCTGGGAAAACTTTGGAGGCTTCTCAACGAGGTGGAGAAGCGCCCGTTTGTGGAGGAAGCTGAA
XiMacTFS9b  GAACTCAGCAAAACCCTGGGGAAACTTTGGAGGCTTCTCAATGAGGTGGAGAAGCGCCCGTTTGTGGAGGAAGCTGAA
EpCoiS2     GAACTCAGCAAAACCCTGGGCAAACTCTGGAGATTGCTCAACGAAGTAGAGAAGCGCCCATTCGTGGAAGAAGCTGAG
TaRubTFS9a  GAATTGAGCAAAACTCTGGGCAAACTCTGGAGATTACTGAATGAGGTGGAGAAGCGGCCGTTCGTGGAGGAGGCAGAG
TeNigTFS9b  GAACTGAGCAAAACCCTGGGCAAACTCTGGAGATTACTCAATGAGGTGGAGAAGCGGCCGTTCGTGGAGGAGGCAGAG
OrNilTFS9b  GAACTCAGCAAAACCCTGGGCAAACTTTGGAGATTGCTCAATGAGGTAGAGAAGCGCCCATTTGTGGAGGAGGCTGAG
CiMonTFS9   GAACTCAGCAAAACCCTTGGCAAACTCTGGAGATTGCTCAATGAGGTAGAGAAGCGCCCGTTTGTGGAAGAGGCTGAG
CySemSHB    GAGCTCAGCAAAACTCTGGGCAAACTTTGGAGATTGCTCAACGAGGTTGAGAAGCGTCCGTTTGTAGAGGAGGCAGAG
OrLatSCC    GAGCTCAGCAAAACTCTTGGAAAACTTTGGAGGCTCCTTAATGAGGTGGAGAAGCGGCCGTTTGTGGAGGAGGCTGAG
GaAcuTFS9b  GAACTCAGCAAAACCCTGGGAAAACTTTGGAGATTGCTCAACGAAGCAGAGAAGCGCCCGTTTGTGGAAGAAGCTGAG
SaSalTFS2   GAACTCAGCAAAACCCTCGGGAAACTCTGGAGATTACTCAACGAAGGCGAGAAGCGTCCGTTCGTAGAGGAGGCTGAA
GaMorS2     GAACTCAGCAAGACTCTTGGCAAACTTTGGAGACTTTTAAACGAAGGGGAGAAGCGCCCGTTTGTGGAAGAAGCCGAG
AsMexTFS9b  GAGCTCAGCAAGACCCTGGGAAAACTGTGGAGGCTGCTGAACGAAGGTGAGAAGCGCCCGTTCGTGGAGGAGGCCGAG
```

```
ClGarS       GAACTCAGCAAAACCCGGGGCAAACTCTGGCGGCTGCTGAATGAAGGGGAGAAGCGGCCATTTGTCGAAGAGGCTGAG
CyCarHTF13   GAGCTCAGCAAGACCCTCGGAAAACTCTGGAGGTTACTGAACGAGGGCGAGAAGCGTCCGTTCGTGGAGGAGGCCGAG
ClGarS1      GAGCTGAGCAAAACCCTGGGCAAGTTATGGAGATTGCTGAACGATACAGAGAAGCGTCCATTCGTAGAAGAGGCTGAG
DaRerHBTF1   GAGCTCAGCAAAACACTCGGCAAACTCTGGAGACTGCTGAACGAGGGCGAGAAGCGTCCGTTTGTGGAGGAGGCGGAG

          547
HoSapSSDR3   CGGCTGCGCGTGCAGCACAAGAAGGACCACCCGGATTACAAGTACCAGCCGCGGCGGAGGAAGTCGGTGAAGAACGGG
MuMusSCG1    CGGCTGCGCGTGCAGCACAAGAAAGACCACCCCGATTACAAGTACCAGCCCCGGCGGAGGAAGTCGGTGAAGAACGGA
CaLupS1      CGGCTGCGCGTGCAGCACAAGAAAGACCACCCGGATTACAAGTACCAGCCGCGGCGGAGGAAGTCGGTGAAGAACGGC
GaGalSTF2    CGGCTGCGGGTGCAGCACAAGAAGGACCACCCCGACTACAAGTACCAACCACGCAGGAGGAAGACGGTGAAGAACGGG
CoJapSSDRY   CGGCTGCGGGTGCAGCACAAGAAGGACCACCCTGACTACAAGTACCAACCACGCAGGAGGAAGTCAGTGAAGAACGGG
AlMisS1      CGGCTGCGGGTGCAGCACAAGAAGGACCACCCTGACTACAAGTACCAGCCCCGGAGGAGAAAGTCGGTGAAGAACGGG
TrScrHCC     AGGCTGCGGGTGCAGCACAAGAAGGACCATCCCGACTACAAGTACCAGCCCCGGAGGAGAAAGTCAGTGAAGAATGGG
LeOliS       AGGCTGCGGGTCCAGCACAAGAAGGACCATCCCGACTACAAGTACCAGCCCCGGAGGAGAAAGTCAGTGAAGAACGGG
AnPlaSSDRY   CGGCTGCGGGTGCAGCACAAGAAGGACCACCCCGACTACAAGTACCAGCCACGGCGGAGGAAGTCGGTGAAGAACGGG
LaChaTFS9    CGACTGAGGGTCCAGCACAAAAAAGACCACCCCGATTACAAGTACCAGCCGCGGAGGAGAAAGTCGGTCAAGAACGGA
XeLaeTFS1    AGGCTGAGGGTCCAACACAAGAAGGATCATCCCGACTACAAGTATCAGCCACGGCGCAGAAAGTCCGTTAAGAATGGG
BuBufTFSP    AGGCTGCGAATCCAACATAAGAAGGATCATCCAGACTACAAGTACCAGCCACGGCGCAGAAAGTCCGTAAAGAATGGG
XeSilTFS9    AGGCTGCGAATCCAACATAAGAAGGATCATCCAGACTACAAGTACCAGCCACGGCGCAGAAAGTCCGTAAAGAATGGG
BuGarS       AGACTGAGAATCCAGCACAAGAAGGACCATCCGGACTACAAGTACCAGCCACGACGAAGAAAGTCGGTCAAGAACGGT
RhMarS       AGGCTGAGAATCCAGCACAAGAAGGACCATCCGGACTACAAGTACCAGCCACGCCGAAGAAAGTCAGTCAAGAACGGT
GlRugSAA     CGTCTGAGAATCCAGCACAAGAAGGATCACCCTGACTACAAGTACCAGCCCCGCCGTAGAAAGTCTGTGAAGAACGGG
PlWalS       AGACTGCGTGTCCAGCACAAGAAGGACCACCCGGACTACAAGTACCAGCCCCGCAGGCGGAAGTCTGTGAAGAACGGC
ScCanS1      CGCCTGAGAGTGCAACACAAGGAGGACCACCCCGATTACAAATACCAGCCCAGGCGGAGAAAGTCGGTGAAGAACGGG
DaRerHBTFS   CGCCTTCGGGTGCAGCACAAGAAAGATCACCCCGACTATAAGTACCAGCCCCGGCGGAGGAAGTCGGTGAAGAACGGC
MoAlbHBTF1   CGGCTCCGGGTGCAGCACAAGAAGGATCATCCGGACTACAAATATCAGCCCCGGCGGAGGAAGTCGGTGAAGAACGGT
OrNilTFS9a   CGTCTCCGGGTGCAGCACAAGAAGGATCACCCGGACTACAAATACCAGCCCCGAAGGAGGAAGTCTGTAAAGAACGGG
GaAcuTFS9a   CGGCTCCGGGTGCAGCACAAGAAGGACCACCCCGACTACAAATACCAGCCCCGGCGGAGGAAGTCCGTCAAGAACGGC
XiMacTFS9a   AGGCTCCGGGTGCAGCACAAGAAGGACCACCCGGACTACAAGTACCAGCCCCGGCGACGGAAGTCGGTGAAGAACGGC
TaRubTFS9b   CGGCTTCGGGTGCAGCACAAGAAGGATCACCCGGACTACAAGTACCAGCCGAGGCGGAGGAAGTCTGTGAAGAACGGC
TeNigTFS9a   CGGCTCCGGGTCCAACACAAGAAGGATCACCCGGACTACAAGTACCAGCCGCGGCGAAGGAAGTCTGTGAAGAACGGC
OrLatSCC1    CGGCTCCGCGTGCAGCATAAGAAGGACCACCCGGACTACAAGTACCAGCCGCGACGGCGGAAGTCCGTGAAGAGCGGC
AsMexTFS9a   CGACTGCGAGTGCAGCACAAGAAGGATCACCCAGACTACAAGTATCAGCCACGGAGGAGGAAGTCGGTGAAGAACGGC
GaMorS1      CGACTCCGCGTGCAGCATAAGAAGGACCACCCCGACTACAAGTACCAGCCCCGGCGGAGGAAGTCCGTGAAGAACGGC
MoAlbHBTFS   CGTTTGAGAGTGCAGCATAAGAAGGATCACCCCGACTACAAATATCAGCCAAGGCGGAGAAAATCTGTCAAGAACGGG
OdBonTFS     CGACTCAGAGTGCAACATAAGAAGGATCACCCCGACTACAAATATCAGCCAAGGCGGAGAAAATCGGTCAAGAATGGT
PoRetS       CGCCTGAGAGTGCAGCACAAGAAGGATCACCCCGACTATAAATATCAACCGAGGCGGAGGAAATCTGTTAAGAATGGG
XiMacTFS9b   CGCCTGAGAGTGCAGCACAAGAAGGATCACCCCGACTATAAATATCAACCGAGGCGGAGGAAATCTGTTAAGAATGGG
EpCoiS2      CGTCTGAGAGTGCAGCACAAGAAAGACCACCCCGACTACAAATATCAGCCACGGCGGAGAAAATCTGTCAAGAACGGG
TaRubTFS9a   CGTCTGAGGGTGCAGCACAAGAAAGACCACCCCGACTACAAGTACCAGCCGAGGCGGAGAAAATCTGTTAAAAACGGC
TeNigTFS9b   CGTTTGAGGGTGCAGCACAAGAAAGACCACCCTGACTACAAGTACCAACCGAGGCGAAGGAAATCCGTCAAAAACGGG
OrNilTFS9b   CGTTTGCGAGTGCAACATAAGAAGGATCACCCCGATTACAAATATCAGCCAAGGCGGAGAAAGTCTGTCAAGAACGGG
CiMonTFS9    CGTTTGAGAGTGCAACATAAGAAGGATCACCCCGATTACAAATATCAGCCAAGGCGGAGAAAATCTGTCAAGAACGGG
CySemSHB     CGCCTGAGGGTGCAGCACAAAAAAGATCACCCAGATTACAAATATCAGCCAAGGCGGAGAAAATCTGTCAAGAACGGA
OrLatSCC     CGACTGAGGGTCCAGCACAAGAAGGATCATCCCGACTATAAATACCAGCCCAGGCGGAGAAAATCTGTCAAGAACGGT
GaAcuTFS9b   CGTTTGAGAGTCCAGCACAAGAAGGACCACCCCGACTACAAATATCAGCCCAGGCGGAGAAAGTCCGTGAAAAACGGG
SaSalTFS2    CGCTTGAGGGTGCAACACAAGAAAGATCACCCCGACTACAAGTACCAGCCCAGAAGGAGAAAATCCGTGAAGAACGGG
GaMorS2      CGCTTAAGGGTTCAACACAAAAAGGACCACCCGGACTACAAATACCAACCGAGGCGGAGGAAATCTGTCAAAAACGGT
AsMexTFS9b   CGACTGAGGGTTCAGCACAAGAAGGACCATCCCGACTACAAGTACCAGCCGAGGAGGAGGAAGTCGGTGAAGAACGGC
ClGarS       AGATTGCGAGTACAGCATAAGAAAGATCATCCGGACTACAAATATCAGCCACGCCGGCGGAAGAGTGTAAAGGGCGGC
CyCarHTF13   CGTCTGAGGGTGCAGCACAAGAAAGACCACCCCGACTACAAGTACCGGCCCAGACGGAGAAAATCAGTGAAGAACGGC
ClGarS1      CGCCTACGCGTGCAACACAAGAAAGATCATCCCGACTACAAGTACCAGCCACGGCGAAGGAAATCAGCCAAGAACGGT
DaRerHBTF1   CGGCTGCGGGTCCAGCATAAGAAAGATCATCCAGACTACAAATACCAGCCCAGACGGAGGAAATCAGTGAAGAGCGGC

          625
HoSapSSDR3   CAGGCGGAGGCAGAGGAGGCCACGGAGCAGACGCACATCTCCCCCAACGCCATCTTCAAGGCGCTGCAG---GCCGAC
MuMusSCG1    CAAGCGGAGGCCGAAGAGGCCACGGAACAGACTCACATCTCTCCTAATGCTATCTTCAAGGCGCTGCAA---GCCGAC
CaLupS1      CAGGCGGAGGCCGAGGAGGCCACCGAACAGACGCACATTTCCCCCAACGCCATCTTCAAGCGCCTGCAG---GCCGAC
GaGalSTF2    CAGTCGGAGCAGGAGGAGGGCTCCGAGCAGACCCACATCTCCCCCAACGCCATCTTCAAGGCGCTGCAG---GCGGAC
CoJapSSDRY   CAGTCGGAGCAGGAGGAAGGCTCCGAGCAGACCCACATCTCCCCCAATGCCATCTTCAAGGCGCTGCAG---GCGGAC
AlMisS1      CAGTCGGAGCAGGAGGAGGGCTCGGAGCAAACCCACATCTCCCCCAATGCCATCTTCAAGGCCCTGCAG---GCGGAC
TrScrHCC     CAGTCGGAGCAGGAGGAGGGCTCCGAGCAAACACACATCTCCCCCAATGCCATTTTCAAGGCCCTGCAG---GCGGAT
LeOliS       CAGGCGGAGCAGGAGGAGGGCTCCGAGCAAACACACATCTCCCCCAATGCCATTTTCAAGGCCCTGCAG---GCGGAT
AnPlaSSDRY   CAGTCGGAGCAGGAGGAAGGCTCCGAGCAGACCCACATCTCCCCCAACGCCATCTTCAAGGCGCTGCAG---GCGGAT
LaChaTFS9    CAGAACGAGCAAGAGGAGGGCACCGAGCAGACCCACATCTCCCCCACTGCTATCTTCAAGGCCCTACAG---GCTGAC
XeLaeTFS1    CAGACAGAACAAGAGGATGGTGCTGAGCAAACCCACATCTCCCCTAATGCAATTTTCAAGGCCCTACAG---GCTGAC
BuBufTFSP    CAGTCAGAACAAGAGGACGGCGCTGAGCAAACCCACATCTCCCCTAATGCCATTTTTAAGGCCCTGCAG---GCTGAT
XeSilTFS9    CAGTCAGAACAAGAGGACGGCGCTGAGCAAACCCACATCTCCCCTAATGCCATTTTTAAGGCCCTGCAG---GCTGAT
```

```
BuGarS       CAGGCTGAGCAAGAGGACGGTTCCGAGCAGACCCACATCTCACCCAATGCCATCTTCAAGGCTCTGCAG---GCTGAC
RhMarS       CAGGCTGAGCAAGAGGACGGTTCCGAGCAGACCCATATCTCGCCCAATGCCATCTTCAAGGCTCTGCAG---GCTGAC
GlRugSAA     CAGTCTGAGCAAGAGGACGGCTCCGACCAGACCCACATCTCTCCCAACGCCATCTTCAAGGCTCTGCAG---GCCGAC
PlWalS       CAGGCGGAGACGGAGGAGGGCTCCGAGCAAACGCACATCTCTCCCAACGCCATCTTCAAGGCGCTGCAG---GCCGAC
ScCanS1      CAGGGGGAGAGCGAGGAGGGAGCCGAGCAGACGCACATCACCCCCAACGCCCTCTTCAAAGCCCTGCAG---GCGGAT
DaRerHBTFS   CAGAGCGAATCTGAAGACGGCAGCGAACAGACCCACATCTCGCCCAACGCCATCTTCAAAGCGCTCCAGCAGGCGGAC
MoAlbHBTF1   CAGAGCGAGCCTGAGGACGGCAGCGAGCAGACGCAC---------AATGCCATCTTCAAAGCGCTCCAACAGGCGGAC
OrNilTFS9a   CAAAGCGAGGGCGAGGACGGGAGCGAGCAGACGCACATTTCCCCAAACGCCATCTTCAAAGCTCTCCAGCAGGCGGAC
GaAcuTFS9a   CAGAGCGAGTCGGAGGACGGCAGTGAGCAGACGCACATTTCCCCCAATGCCATCTTCAAAGCTCTCCAGCAGGCGGAC
XiMacTFS9a   CAGAGCGAGGCGGAGGACGGCGGCGAGCAGACGCACATCTCCACCAACGCCATTTTCAAAGCCCTCCAGCAGGCTGAC
TaRubTFS9b   CAGAACGAGTCGGACGACGGCAGCGAGCAGACGCACATTTCCCCCAACGCCATTTTCAAAGCTCTCCAACAGGCGGAC
TeNigTFS9a   CAGAACGAGTCGGACGACGGCGGCGAGCAGACGCACATTTCCCCCAACGCAATTTTCAAAGCTCTCCAGCAGGCGGAC
OrLatSCC1    GGGAGCGAGGCGGAGGACGGCGGGGAG------CACATCTCCACCAACGCCATCTTCAAGGCTCTGCAGCAGGCGGAC
AsMexTFS9a   TCTGGCGAGGGGGAGGACGGCTCGGACCATGCTCACGTGTCGCCTAACGCCATCTTCAAAGCCCTCCAGCAGGCCGAC
GaMorS1      CAGGGAGAGTCAGAGGACGGCGGGGAGCAGACGCACATCACGCCCAACGCCATATTCAAGGCGCTGCAGCAGGCGGAC
MoAlbHBTFS   CAAAACGAGCCGGAGGACAGC---GAGCAAACTCACATCTCTCCAAATGCGATCTTCAAGGCGCTACAGCAGGCCGAC
OdBonTFS     CAAAGCGAGCCCGAGGACAAC---GAGCAAACTCACATCTCTCCAAATGCGATCTTCAAAGCACTTCAGCAGGCCGAC
PoRetS       CAAAATGAATCCGAGGATGGC---GAACAGACGCACATCTCTCCGAACGCAATTTTCAAGGCGCTGCAGCAAGCCGAC
XiMacTFS9b   CAAAATGACTCCGAGGATGGC---GAACAGACGCACATCTCTCCGAACGCAATTTTCAAGGCGCTGCAGCAAGCCGAC
EpCoiS2      CAAAACGACCCCGAGGACGGC---GAGCAAACCCACATCTCTCCAAATGCCATATTCAAGGCGCTGCAGCAGGCCGAT
TaRubTFS9a   CAAAACGACCCCGAGGACGGA---GAGCAAACCCACATCTCACCCAATGCCATCTTCAAGGCGCTGCAGCAGGCCGAC
TeNigTFS9b   CAAAACGACTCCGAGGACGGA---GAGCAAACCCACATCTCACCCAATGCCATTTTCAAGGCGCTGCAGCAGGCCGAC
OrNilTFS9b   CAAAGCGAACCGGAGGACAAC---GACCAAACTCATATCTCTCCAAATGCCATTTTCAAGGCGCTGCAGCAGGCCGAC
CiMonTFS9    CAAAGCGAAGCGGAGGACAGC---GAACAAACTCACATCTCTCCAAATGCCATTTTCAAGGCACTGCAGCAGGCCGAC
CySemSHB     CAAAATGAAGCAGACGACGGC---GAGCAGACGCACATTTCTCCGAATGCGATCTTCAAGGCCCTGCAGCAGGCCGAT
OrLatSCC     CAAAGCGAAGCGGAGGACAGC---GAACCGACTCACATCTCTCCAAATGCGATCTTCAAAGCGCTGCAGCAGGCCGAC
GaAcuTFS9b   CAAAACGACCCCGAGGACGGC---GAGCAAACCCACATCTCTCCAAATGCGATCTTCAAGGCTCTGCAGCAGGTCGAT
SaSalTFS2    CAGAGCGAGCCAGAGGACGGC---GAGCAAACCCACATCTCTTCCGGTGACATCTTTAAAGCTCTCCAGCAAGCCGAC
GaMorS2      CAGAGCGAGTCCGAAGACGGC---GAGCAAACACACATATCTCCAAATGCCATCTTCAAGGCGCTGCAGCAAGCCGAC
AsMexTFS9b   CAGTCGGAGTCGGAGGACGGC---GAGCAGACCCACATCTCGCCCAACGCCATCTTCAAAGCCCTGCAGCAAGCCGAC
ClGarS       CAGGGTGAAACCGAGGATACC---GAGCACACCCACATTTCCCCCAATGCCATCTTTAAAGCCCTACAGGGAGCCGAC
CyCarHTF13   CAGAGCGAGAGCGAGGACGGC---GAGCAGACCCACATCTCACCCAACGCCATCTTCAAAGCCCTGCAGCAGGCCGAC
ClGarS1      CAGGGCGAGGGGGAAGAGGGTCCCGAGCACACGCACGTCTCCACCAACGCCATCTTCAAAGCACTGCAACAAGCCGAT
DaRerHBTF1   TCAGCAGAGTCTGAAGATGGA---GAGCAGACGCAGATCTCCACCAATGCGCTGTTCAGAGCCCTGCAGCGGGCAGAA

          703
HoSapSSDR3   TCGCCACACTCCTCCTCCGGCATGAGCGAGGTGCACTCCCCCGGCGAGCACTCG---GGGCAATCCCAGGGCCCACCG
MuMusSCG1    TCCCCACATTCCTCCTCCGGCATGAGTGAGGTGCACTCCCCGGGCGAGCACTCT---GGGCAATCTCAGGGTCCGCCG
CaLupS1      TCGCCGCACTCCTCCTCCGGCATGAACGAGGTGCACTCCCCCGGCAAGCACTCG---GGGCAATCCCAGGGCCCGCCG
GaGalSTF2    TCCCCGCAGTCATCCTCCAGCATCAGCGAGGTGCACTCCCCCGGGGAGCACTCA---GGGCAGTCGCAGGGCCCCCCC
CoJapSSDRY   TCCCCACAGTCATCCTCCAGCATCAGCGAGGTGCATTCCCCAGGGGAGCACTCA---GGGCAGTCACAGGGCCCTCCT
AlMisS1      TCCCCGCAGTCCTCTTCCAGCATGAGCGAGGTGCACTCCCCCGGGGAGCACTCT---GGGCAGTCCCAAGGCCCCCCG
TrScrHCC     TCTCCTCAGTCTTCCTCCAGCATGAGCGAGGTTCACTCTCCCGGGGAGCACTCT---GGGCAGTCCCAAGGGCCCCCG
LeOliS       TCTCCTCAGTCTTCCTCCAGCATGAGTGAGGTTCACTCTCCAGGAGAGCATTCA---GGGCAGTCCCAGGGGCCCCCG
AnPlaSSDRY   TCCCCGCAGTCGTCCTCCAGCATCAGCGAGGTGCACTCCCCCGGGGAGCACTCG---GGGCAATCGCAGGGCCCCCCC
LaChaTFS9    TCGCCCCACTCTGCTTCAAGTATGAGCGAGGTCCACTCTCCTGGGGAACACTCA---GGCCAGTCCCAAGGCCCTCCA
XeLaeTFS1    TCCCCGCATTCCTCTTCCAGCATGAGCGAAGTCCACTCTCCTGGAGAACATTCA---GGTCAATCCCAAGGCCCACCA
BuBufTFSP    TCCCCACATTCTGCCTCCAGCATGAGCGAAGTCCACTCTCCTGGAGAACATTCA---GGTCAGTCCCAAGGCCCACCA
XeSilTFS9    TCCCCACATTCTGCCTCCAGCATGAGCGAAGTCCACTCTCCTGGAGAACATTCA---GGTCAGTCCCAAGGCCCACCA
BuGarS       TCCCCACATTCCGCCTCCAGCATGAGCGAGGTGCACTCCCCAGGGGAGCACTCA---GGTCAGTCACAGGGGCCACCA
RhMarS       TCCCCACATTCTGCCTCCAGCATGAGCGAGGTGCACTCCCCCGGGGAGCACTCA---GGTCAGTCACAGGGGCCACCA
GlRugSAA     TCGCCGCACTCTGCTTCCAGCATGAGCGAAGTGCACTCTCCAGGAGAACACTCA---GGGCAGTCGCAAGGTCCACCA
PlWalS       TCACCGCACTCATCATCCGGCATGAGCGAGGTGCACTCGCCCGGAGAGCACTCT---GGCCAGTCCCAGGGTCCACCC
ScCanS1      TCCCCGCACTCCGCCTCCAGCATGAGCGACGTGCATTCCCCAGGGGAACATTCA---GGACAATCTCAAGGACCACCA
DaRerHBTFS   TCGCCC------GCGTCCAGCATGGGAGAAGTGCACTCGCCCAGCGAACACTCA---GGCCAGTCCCAGGGGCCGCCC
MoAlbHBTF1   TCTCCG------GCCTCCAGCATGGGAGAGGTGCATTCTCCGAGTGAGCACTCA---GGC---TCCCAGGGGCCCCCT
OrNilTFS9a   TCCCCT------GCATCCAGCATGGGAGAGGTGCATTCTCCTGGAGAGCACTCA---GGC---TCCCAGGGTCCCCCT
GaAcuTFS9a   TCCCCA------GCCTCCAGCATGGGAGAGGTGCACTCACCGGGTGAGCACTCA---GGC---TCCCAGGGCCCCCCT
XiMacTFS9a   TCCCCC------GCCTCCAGCATGGGAGAGGTGCACTCCCCCGCCGAGCACTCA---GGC---TCACAGGGTCCCCCC
TaRubTFS9b   TCCCCA------GCCTCCAGCATGGGAGAGGTACACTCTCCAGGTGAGCACTCA---GGC---TCTCAGGGCCCTCCT
TeNigTFS9a   TCCCCG------GCCTCCAGCATGGGAGAGGTGCACTCTCCAGGTGAGCACTCA---GGC---TCTCAGGGTCCTCCC
OrLatSCC1    TCCCCC------GCCTCCAGCATGGGGGAGGTGCACTCCCCCGCGGAGCACTCA---GGC---TCACAGGCACCCCCA
AsMexTFS9a   TCCCCG------GCGTCCAGCATGGGAGAGGTGCACTCGCCCAGCGAACAAGGA---GGCCAGTCCCAAGGTCCCCCG
GaMorS1      TCCCCG------GCCTCCAGCATGGGGGACGTGCACTCACCTGGCGAGCACTCA---GGC---TCCCAGGGGCCCCCG
MoAlbHBTFS   TCTCCG------GCGTCCAGTATGGGCGAGGTGCACTCTCCAGGCGACCATTCA---GGTCAGTCCCAGGGTCCGCCA
OdBonTFS     TCTCCA------GCGTCCAGTATGGGCGAGGTGCACTCTCCTGGAGAACATTCA---GGTCAGTCACAGGGCCCACCA
PoRetS       TCTCCT------GCGTCCAGCATGGGCGAGGTGCACTCACCAGGAGAACATTCG---GGTCAGTCACAGGGACCACCA
XiMacTFS9b   TCTCCT------GCGTCCAGCATGGGCGAGGTGCACTCTCCGGGAGAACATTCG---GGTCAGTCACAGGGACCACCA
```

```
EpCoiS2      TCTCCT------GCGTCCAGTATGGGCGAGGTGCATTCTCCAGGAGAGCACTCA---GGTCAGTCCCAGGGCCCACCG
TaRubTFS9a   TCCCCA------GCCTCGAGTTTGGGCGAAGTTCATTCTCCAGGCGATCACTCA---GGTCAGTCCCAGGGCCCGCCG
TeNigTFS9b   TCCCCA------GCCTCGAGTTTGGGCGAGGTGCACTCTCCAGGCGAGCACTCA---GGTCAGTCCCAGGGCCCACCG
OrNilTFS9b   TCTCCG------GCTTCAAGTATGGGCGAGGTGCACTCTCCGGGAGAGCATTCA---GGTCAGTCACAGGGTCCACCA
CiMonTFS9    TCTCCG------GCTTCAAGCATGGGCGAGGTGCATTCTCCAGGAGAGCACTCA---GGTCAGTCACAGGGTCCACCG
CySemSHB     TCCCCT------GCGTCCAGTATGGGCGAGGTGCACTCTCCA---GAACATTCA---GGTCAATCCCAAGGTCCACCC
OrLatSCC     TCTCCT------GCGTCCAGCATGGGCGAGGCGCACTCTCCAGGAGAACATTCA---GGTCAGTCACAGGGGCCACCA
GaAcuTFS9b   TCTCCA------GCTTCAAGTTCGGGCGAGGTGCATTCTCCAGGAGAGCATTCA---GGTCCGTCCCAGGGCCCTCCA
SaSalTFS2    TCACCC------GCGTCCAGCATGGGCGAAGTGCATTCTCCCGGTGAACATTCAGCAGGCCAGTCCCAGGGCCCACCT
GaMorS2      TCCCCA------ACCTCTAGCATGGGGGAGGTGCACTCCCCCGGCGAGCACTCA---GGTCAGTCCCAAGGCCCACCG
AsMexTFS9b   TCGCCC------GCCTCCAGCATGGGCGAAGTGCACTCCCCCTCAGACCACTCA---GGTCAATCCCAAGGTCCTCCA
ClGarS       TCTCCC------GCTTCCAGCATGGGTGAGGTACACTCTCCCGGAGACCACTCA---GGTCAGTCCCAGGGCCCTCCA
CyCarHTF13   TCCCCC------GCGTCCAGCATGGGCGAAGTGCACTCTCCGGGAGACCACTCA---GGTCAGTCCCAGGGTCCCCCG
ClGarS1      TCTCCA------GCATCCAGCATGGGAGAGGTGCACTCCCCCAGCGAGCAGTCA---GGCCAGTCCCAGGGTCCCCCG
DaRerHBTF1   ACACCC------GACTCCAGCACCGGCGAGCTGCACTCTCCTGGAGAACACTCC---GGTCAGTCTCAGGGCCCCCCC

          781
HoSapSSDR3   ACCCCACCCACCACCCCCAAAACCGACGTG---CAGCCGGGCAAGGCTGAC---CTGAAGCGAGAG------GGG---
MuMusSCG1    ACCCCACCCACCACTCCCAAAACCGACGTG---CAAGCTGGCAAAGTTGAT---CTGAAGCGAGAG------GGG---
CaLupS1      ACGCCCCCCACCACCCCGAAAACCGACGTG---CAGCCGGGCAAGGCTGAC---CTGAAGCGCGAG------GGC---
GaGalSTF2    ACGCCCCCCACCACCCCCAAAACGGACGCTCAGCAGCCGGGCAAGCAGGAC---CTGAAGCGCGAG------GGC---
CoJapSSDRY   ACACCCCCCACCACTCCCAAGACGGACGCTCAGCAGCCGGGCAAGCAGGAC---CTGAAGCGCGAG------GGC---
AlMisS1      ACACCACCCACGACCCCCAAAACAGACGTC---CAGCCTGGCAAGCAAGAC---CTGAAGCGAGAA------GGA---
TrScrHCC     ACCCCTCCTACTACACCCAAAACAGATGTC---CAGCCTGGGAAGCAAGAC---CTGAAGCGAGAA------GGG---
LeOliS       ACTCCTCCTACTACACCAAAAACAGATGTC---CAGCCTGGGAAGCAAGAC---CTGAAGCAAGAA------GGG---
AnPlaSSDRY   ACGCCCCCCACCACCCCCAAGACGGACGCG---CAGCCGGGCAAGCAGGAC---CTGAAGCGCGAG------GGC---
LaChaTFS9    ACTCCCCCTACTACTCCCAAAACAGATGTC---CAGCCAGGAAAACCAGAT---CTGAAACGAGAG------GTA---
XeLaeTFS1    ACTCCTCCAACTACTCCCAAGACAGATATC---CAGCCTGGAAAGCCAGAC---CTAAAGAGGGAG------GGC---
BuBufTFSP    ACTCCTCCAACTACCCCCAAGACAGACGTC---CAGCCTGGAAAGCCAGAC---CTGAAGAGGGAG------GGC---
XeSilTFS9    ACTCCTCCAACTACCCCCAAGACAGACGTC---CAGCCTGGAAAGCCAGAC---CTGAAGAGGGAG------GGC---
BuGarS       ACTCCACCAACCACCCCTAAAACAGATGTG---CAGCCCGGCAAGCCAGAC---CTGAAGCGCGAA------GGA---
RhMarS       ACTCCACCGACCACCCCTAAAACAGATGTC---CAGCCCGGCAAGCCAGAC---TTGAAGCGTGAA------GGA---
GlRugSAA     ACTCCGCCGACCACCCCTAAAACAGATGTG---CAGCCCGGCAAGCCAGAT---CTGAAACGCGAA------GGT---
PlWalS       ACACCTCCAACTACCCCAAAAACCGACGTT---CAGCCAGGGAAACCTGAC---CTGAAGCGGGAA------GGA---
ScCanS1      ACGCCCCCCACCACTCCCAAAACAGAGGTT---CAGCCTGTTAAACCAGAT---CTGAAACGTGAA------GGT---
DaRerHBTFS   ACTCCTCCCACCACCCCGAAAACTGACACACAGCCA---GGCAAAGCGGAT---CTGAAACGAGAGGCC---------
MoAlbHBTF1   ACCCCTCCCACCACGCCAAAGACTGATGTCACCTCA---GGCAAGATGGAC---CTAAAGCGTGAAGGGGGGCTC---
OrNilTFS9a   ACCCCTCCCACTACCCCAAAGACTGATGTCAGCTCA---GGCAAGGTCGAC---CTAAAACGTGAAGTAGGCCTC---
GaAcuTFS9a   ACTCCTCCCACCACCCCAAAGACTGATGTCAGCTCG---GGCAAGATGGAC---CTAAAGCGAGAGGGTGGCATC---
XiMacTFS9a   ACCCCTCCCACCACCCCTAAGACCGACGTTGGCTCA---GGTAAGACGGAC---CTGAAGCGCGAGGCGGGGCTC---
TaRubTFS9b   ACCCCTCCCACTACCCCAAAGACTGATGTCACCTCA---GTCAAGATGGAT---CTAAAGCGTGAAGGCGGCCTC---
TeNigTFS9a   ACCCCTCCCAGTACCCCAAAGACTGATGTCACCTCA---GTCAAGATAGAT---CTCAAACGTGAAGGCAGCCTT---
OrLatSCC1    ACCCCTCCCACCACCCCAAAGACGGACTGC---TCA---GCAAAGATGGAC---CTAAAGCGTGAGGGGGGGGCTG---
AsMexTFS9a   ACTCCCCCCACCACCCCCAAAATCGACACACAGCCA---GGCAAAGTGGAC---CTTAAGCGCGAGGGC---------
GaMorS1      ACTCCTCCCACCACCCCCAAAACAGACGTCATCTCA---GGCAAGATGGAC---CTGAAGCGCGAGGCGGGCGTG---
MoAlbHBTFS   ACACCCCCAACCACCCCCAAAACAGACCTCTCCTCC---AGCAAAGCTGAT---CTGAAGCGTGAG------GGA---
OdBonTFS     ACACCCCCAACAACCCCCAAGACAGACGTCCCCACC---AGTAAAGCTGAC---CTAAAGCGTGAG------GGA---
PoRetS       ACACCCCCGACAACCCCAAAGACAGATCTTCCCTCC---AGTAAAGCTGAC---CTGAAGCGTGAG------GGC---
XiMacTFS9b   ACACCTCCGACAACCCCAAAGACAGATCTTCCCTCC---AGCAAAGCTGAC---CTGAAGCGTGAG------GGC---
EpCoiS2      ACCACCCCCACCACCCCCAAGACAGACCTCCCCTCC---AGCAAAGCTGAC---CTGAAGCGTGAG------GGG---
TaRubTFS9a   ACACCCCCGACCACTCCCAAGACGGACTTGGTCTCC---AGCAAAGCTGAT---CTGAAACGTGAG------GGG---
TeNigTFS9b   ACACCCCCGACCACTCCCAAGACAGACCTGGTCTCT---GGCAAGGCTGAT---CTGAAACGAGAG------GGC---
OrNilTFS9b   ACACCCCCAACCACCCCAAAGACAGACCTTCCCTCC---AGCAAAGCTGAT---CTGAAACGTGAG------GGG---
CiMonTFS9    ACACCCCCAACCACCCCGAAGACAGACCTTCCCTCC---AGCAAAACTGAC---CTGAAGCGTGAG------GGG---
CySemSHB     ACACCCCCGACCACCCCTAAAACAGACATGCCCTCC---AATAAAGCCGAA---CTGAAGCGTGAA------GGG---
OrLatSCC     ACACCCCCAACAACCCCCAAGACAGATCTTCCTTCC---AGCAAAGCTGAC---CTGAAGCGCGAG------GGC---
GaAcuTFS9b   ACACCCCCAACCACCCCCAAGACAGACCTGCCCTCC---AGCAAGGCTGAC---CTGAAGCGAGAG------GGT---
SaSalTFS2    ACACCACCGACCACCCCCAAAACAGACCTGGCTGCG---GGCAAGGCCGAC---CTGAAGCGTGAG------GGC---
GaMorS2      ACGCCCCCCACCACCCCCAAGACCGACCTGCCCTCAGGGGGCAAGGGGGAGATCCTGAAGCGCGAGGCTTCGGGC---
AsMexTFS9b   ACGCCACCGACAACCCCTAAAACAGACCTTCCCGGC---AGCAAACCAGAC---CTAAAGCGCGAG------GGA---
ClGarS       ACTCCCCCCACAACTCCCAAAACAGATGTTCCAAGT---GTGAAACCGGAC---CTGAAGCGAGAG------GGC---
CyCarHTF13   ACTCCTCCCACGACCCCCAAAACGGACCTGCCGTCC---AGCAAAGCGGAT---CTGAAGCGCGAG------GGC---
ClGarS1      ACTCCACCCACCACCCCCAAAATTGATGCTCAGGCA---GGCAAAAGTGAG---CCAAAGCGTGAG------GGA---
DaRerHBTF1   ACACCACCCACCACCCCTAAAACAGATCTGCCGGTGTGCAGTAAAGCGGAT---CTGAAGCGGGAGCGGGAGCGGGAC

          859
HoSapSSDR3   ------CGCCCCTTGCCAGAGGGG---------------------------GGCAGACAGCCCCCT---ATCGACTTC
MuMusSCG1    ------CGCCCTCTGGCAGAGGGG---------------------------GGCAGACAGCCCCCC---ATCGACTTC
```

```
CaLupS1      ------CGCCCCCTGCCCGAGGGG---------------------------GGCCGACAGCCCCCC---ATCGACTTC
GaGalSTF2    ---------CCTTTGGCGGAAGGC---------------------------GGCCGCCAACCTCCCCACATCGATTTC
CoJapSSDRY   ------CGCCCTTTGGCAGAAGGT---------------------------GGCCGCCAACCTCCCCACATTGATTTC
AlMisS1      ------CGCCCCCTGCAAGAGGGA---------------------------GGGCGGCAGCCGCCCCACATTGACTTT
TrScrHCC     ------CGCCCTTTGCAGGAAGGA---------------------------GGAAGACAGCCGCCCCACATTGACTTC
LeOliS       ------CGCCCTTTGCAGGAAGGG---------------------------GGAAGACAACCGCCACACATTGACTTC
AnPlaSSDRY   ------CGCCCCTTGCAAGAAGGC---------------------------GGCCGGCAGCCGCCCCACATCGACTTC
LaChaTFS9    ------CGCCCCCTACAGGAAGGT---------------------------GGAAGGCAG---CCACACATTGACTTT
XeLaeTFS1    ------AGGCCACTGCAAGAGAAC---------------------------GGTAGGCAGCCACCTCACATTGATTTC
BuBufTFSP    ------AGGCCACTGCAGGAGAGC---------------------------GGTAGGCAGCCACCTCACATCGATTTC
XeSilTFS9    ------AGGCCACTGCAGGAGAGC---------------------------GGTAGGCAGCCACCTCACATCGATTTC
BuGarS       ------CGCACCCTGCAAGAGAGC---------------------------GGCCGCCAGCCCCCTCATATTGATTTC
RhMarS       ------CGCACCCTGCAAGAAAGC---------------------------GGCCGCCAGCCCCCTCACATTGATTTC
GlRugSAA     ------CGCCCCTTGCAGGAGAGC---------------------------GGCCGCCAGCCACCCCACATCGATTTC
PlWalS       ------CGCCCTCTCCAGGAGGAA---------------------------GGGAGACAGCCTCCCCACATTGACTTC
ScCanS1      ------CGCCCCCTGCAGGAAGGC---------------------------GGGAGACAG---CCTCACATTGATTTC
DaRerHBTFS   ------CGTCCTCTTCAGGAAAAC------ACC------------------GGACGTCCG---CTCAGCATCAACTTC
MoAlbHBTF1   ------CGCTCTCTGCCCGATGGG------TCTGGT---------------GGGCGCCAG---CTCGACATCGACTTC
OrNilTFS9a   ------CGCTCCCTGCCTGATGGT------CCCGGT---------------GGGCGACAG---CTCAACATCGACTTC
GaAcuTFS9a   ------CGCTCCCTGTCCGACGGC------ACCGGC---------------GGCCGCCAG---CTCAACATCGACTTC
XiMacTFS9a   ------CGCGCTCTGCCCGACGGC------AATGTT---------------GGGCGCCAG---CTCAACATCGACTTC
TaRubTFS9b   ------CGCTCTCTGAACGATGGC------CCCGGA---------------GGACGTCAG---CTCAACATCGACTTC
TeNigTFS9a   ------CGCACTCTGACCGATGGC------CCCGGC---------------GGACGTCAG---CTCAACATCGACTTC
OrLatSCC1    ------CGCCCACTGCCGGACGGC------GCCCCC---------------GGACGCCAG---CTCAACATCGACTTC
AsMexTFS9a   ------CGGCCGCTCCAGGAAGGCGTCAGCAGTGGC---------------GGACGGCAG---CTCAACATCGACTTC
GaMorS1      ------CGCCCCGCTCAGGACGGCCCCTCCCCCTCCGCCGCCGCCGCCGTGGGCCGCCAG---CTCAACATCGACTTC
MoAlbHBTFS   ------CGCCCCATGCAGGAGGGCACC------------------------AGTCGCCAG---CTTAACATTGACTTC
OdBonTFS     ------CGGCCCATGCAGGAGGGCACC------------------------AGCCGCCAG---CTCAACATTGACTTT
PoRetS       ------CGCCCCATTCAAGAGGGCAGC------------------------AGTCGGCAG---CTCAACATAGACTTT
XiMacTFS9b   ------CGCCCCATTCAAGAGGGCAGC------------------------AGTCGGCAG---CTCAACATCGACTTT
EpCoiS2      ------CGCCCCATGCAGGAGGGCACC------------------------AGTCGCCAG---CTCAACATCGACTTT
TaRubTFS9a   ------CGCCCCATGCAGGAGGGCACC------------------------AGCCGCCAG---CTCAACATCGACTTC
TeNigTFS9b   ------CGGCCCACGCAGGAGGGCACA------------------------AGCCGTCAG---CTCAACATCGACTTC
OrNilTFS9b   ------CGGCCCATGCAGGAGGGCACT------------------------AGCCGCCAG---CTCAACATTGATTTT
CiMonTFS9    ------CGGCCCATGCAGGAGGGCACT------------------------AGTCGCCAG---CTCAACATTGATTTT
CySemSHB     ------CGACCCATGCAGGAGGGCGCC------------------------AGTCGTCAG---CTCAACATCGACTTT
OrLatSCC     ------CGCCCAGTGCAAGAGGGCACC------------------------AGCCGCCAG---CTCAACATAGACTTT
GaAcuTFS9b   ------CGCCCCATGCAGGAGGGCACC------------------------AGCCGCCAG---CTCAACATCGACTTT
SaSalTFS2    ------CGCCCCCTGCAAGAGGGCACG------------------------GGCCGCCAG---CTCAACATCGACTTC
GaMorS2      ------CGTCCCGCGCCGGAGGGC------------------------------CGCCAG---CTCAACATCGACTTT
AsMexTFS9b   ------CGCCCCCTGCAGGATGGTGCC------------------------GGTCGCCAG---CTCAACATCGACTTC
ClGarS       ------CGTCCACTTCCCGAGGGCACA------------------------GGCCGCCAA---CTTAACATCGACTTC
CyCarHTF13   ------CGTCCGCTGCAGGAGGGC---------------------------------------------ATTGACTTC
ClGarS1      ------CGCCCATTGCAGGATGGCACCGCAGTTACCGTTGGCTCTGGCGGGCAGCGTCAG---CTTAACATCGACTTT
DaRerHBTF1   CGAGAGCGCCCCCTGCAGGACGGC---------------------------------------------ATTGACTTC

           937
HoSapSSDR3   CGCGACGTGGACATCGGCGAGCTGAGCAGCGACGTCATCTCCAACATCGAGACCTTCGATGTCAACGAGTTTGACCAG
MuMusSCG1    CGCGACGTGGACATCGGTGAACTGAGCAGCGACGTCATCTCCAACATTGAGACCTTCGACGTCAATGAGTTTGACCAA
CaLupS1      CGCGACGTGGACATCGGGGAGCTGAGCAGCGACGTCATCTCCAACATAGAGACCTTCGACGTCAACGAATTCGACCAG
GaGalSTF2    CGAGACGTGGACATCGGCGAGCTCAGCAGCGACGTCATCTCCAACATCGAAACCTTCGACGTCAACGAGTTCGACCAA
CoJapSSDRY   CGAGACGTGGACATCGGTGAACTCAGCAGCGACGTCATCTCCAACATCGAAACCTTTGACGTCAACGAGTTTGACCAA
AlMisS1      CGAGACGTGGACATCGGGGAGCTCAGCAGCGATGTCATCTCCAACATCGAGACCTTCGACGTCAACGAGTTCGACCAG
TrScrHCC     CGGGATGTGGACATTGGGGAACTCAGCAGCGACGTCATCTCCAACATAGAGACCTTTGATGTCAATGAGTTCGACCAG
LeOliS       CGGGATGTGGACATTGGGGAACTCAGCAGCGACGTCATCTCCAACATAGAGACCTTTGACGTAAATGAGTTTGACCAG
AnPlaSSDRY   CGAGACGTCGACATCGGCGAGCTCAGCAGCGACGTCATCTCCAACATCGAAACCTTCGACGTCAACGAATTCGACCAG
LaChaTFS9    AGAGATGTGGACATAGGAGAGCTAAGCAGTGATGTCATCTCCAACATAGAAGCCTTCGATGTCAATGAGTTTGACCAG
XeLaeTFS1    CGAGATGTGGACATTGGTGAGCTGAGCAGTGAGGTCATCTCCACCATTGAAACCTTTGATGTCAATGAATTTGACCAA
BuBufTFSP    CGAGATGTAGATATTGGTGAACTGAGCAGTGAGGTCATCTCTACCATCGAAACCTTTGATGTCAATGAATTTGACCAA
XeSilTFS9    CGAGATGTAGATATTGGTGAACTGAGCAGTGAGGTCATCTCTACCATCGAAACCTTTGATGTCAATGAATTTGACCAA
BuGarS       CGGGACGTGGACATTGGAGAGCTGAGCAGCGAGGTGATCTCCAATATTGAGGCCTTTGATGTCAATGAATTCGACCAG
RhMarS       CGGGACGTGGACATTGGAGAGCTGAGCAGCGAGGTGATCTCCAACATTGAGGCCTTTGATGTCAATGAATTCGACCAG
GlRugSAA     CGCGACGTGGACATCGGAGAGCTCAGCAGCGAGGTCATCTCCAACATCGAGACCTTCGACGTCAACGAGTTCGACCAG
PlWalS       GGGGATGTGGACATCTCGGTGCTGAGCAGTGACGTCATCTCTAACTTTACTCCCATCGACATCCAGGAGTTTGATCAA
ScCanS1      AGCAATGTGGACATTGGGGAACTCAGCAGCGAAGTCATGTCGAACATGGAAACCTTCGACGTCAACGAGTTTGACCAG
DaRerHBTFS   CAGGACGTGGATATCGGCGAGCTGAGCAGCGATGTT---------ATCGAAACATTCGACGTCAATGAGTTTGACCAA
MoAlbHBTF1   CATGATGTGGACATTGGTGAGTTCAGCAGCGATGTCATCTCCCACATTGAAACCTTTGATGTCAATGAGTTCGACCAG
OrNilTFS9a   CGCGACGTGGACATCGGCGAGCTGAGCAGTGATGTCATCTCCCATATTGAGACCTTCGATGTCAACGAGTTCGACCAG
```

```
GaAcuTFS9a  CGCGACGTGGACATCGGCGAGCTGAGCAGCGACGTCATCTCCCACATCGAGACCTTCGACGTCAACGAGTTCGACCAG
XiMacTFS9a  CGGGACGTGGACATCGGCGAGCTGAGCAGCGACGTCATCTCCCACATCGAGACCTTTGATGTCAACGAGTTCGACCAG
TaRubTFS9b  CGTGACGTGGATATTGGCGAGCTGAGCAGTGACGTCATCTCCCACATCGAGACCTTCGATGTCAACGAGTTTGACCAG
TeNigTFS9a  CGTGACGTGGATATTGGCGAGCTTAGCAGCGACGTCATCTCCCACATCGAGACCTTTGATGTCAATGAGTTTGACCAG
OrLatSCC1   CGTGACGTGGACATCGGCGAGCTGAGCAGTGACGTCATCTCCCACATTGAGACGTTCGACGTCAACGAGTTCGACCAG
AsMexTFS9a  CGTGATGTGGACATCGGCGAGCTGAGCAGTGACGTCATCTCACACATGGAGAGCTTCGACGTGGCTGAGTTCGACCAG
GaMorS1     CGCGACGTGGACATCGGCGAGCTGAGCAGCGACGTCATCTCCCACATCGAGACCTTCGACGTCAACGAGTTCGACCAG
MoAlbHBTFS  GGAGCTGTGGATATTGGTGAGCTGAGCAGCGATGTCATCTCCAACATGGGGAGCTTTGATGTGGATGAGTTTGATCAG
OdBonTFS    GGAGCTGTGGACATCGGCGAGCTGAGCAGCGAAGTCATCTCCAACATGGGAAGCTTCGATGTTGATGAGTTTGATCAG
PoRetS      GGAGCTGTGGACATTGGTGAGCTTAGCAGCGACGTCATCTCCAACATCGGCAGCTTTGATGTTGATGAGTTCGACCAG
XiMacTFS9b  GGAGCTGTGGACATTGGTGAGCTTAGCAGCGACGTCATCTCCAACATCGGGAGCTTTGATGTCGATGAGTTTGACCAG
EpCoiS2     GGAGCTGTGGACATTAATGAGCTGAGCAGCGAGGTCATCTCCAACATCGGGAGCTTTGATGTCGACGAGTTCGATCAG
TaRubTFS9a  GGAGCCGTGGACATTGGCGAGCTGAGCAGCGAGGTCATTTCCAACATGGGCAGCTTCGACGTGGACGAGTTCGACCAG
TeNigTFS9b  GGCGCCGTGGACATCGGCGAGCTGAGCAGCGAGGTCATTTCCAACATGGGGAGCTTCGACGTGGACGAGTTCGACCAG
OrNilTFS9b  GGAGCTGTGGACATTGGTGAGCTGAGCACTGATGTCATCTCCAATATAGGAAGCTTCGATGTTGATGAGTTTGATCAG
CiMonTFS9   GGAGCTGTGGACATTGGTGAGCTGAGCAGTGATGTTATCTCCAACATGGGAAGCTTCGATGTTCACGAGTTTGATCAG
CySemSHB    GGAGCGGTGGACATCGGCGAGTTGAGCAGCGACGTCATCTCCAACATGGGGAGCTTTGACGTTGATGAGTTTGATCAG
OrLatSCC    GGCACTGTGGACATCGGTGAGCTGAGCAGCGACGTCATCTCCAACATCGGCAGCTTCGACGTCGATGAGTTCGATCAG
GaAcuTFS9b  GGAGCTGTGGACATCGGCGAGCTGAGCAGCGAGGTCATCTCCAACATGGGCAGCTTCGACGTCGATGAGTTCGATCAG
SaSalTFS2   CGGGACGTGGATATCGGCGAGCTGAGCAGCGACGTCATCTCCAACATCGAGGCCTTCGATGTCAATGAGTTCGACCAG
GaMorS2     GGCGCGGTGGACATCGGCGAGCTGAGCAGCGACGTCATCATCAACATGGGCAGCTTCGACGTGGACGAGTTCGACCAG
AsMexTFS9b  AGGGACGTGGACATTGGCGAGCTGAGCAGCGACGTCATCTCCAACATCGAGACCATCGACGTCAACGAGTTCGACCAG
ClGarS      CGTGATGTGGACATTGGTGAGCTGAGCAGTGACGTCATCTCCAACATGGAGGCATTTGATGTGAATGAGTTTGACCAG
CyCarHTF13  GGCGCCGTAGACATCGGCGAGCTGAGCAGCGACGTCATCTCCAACATGGAGCCATTCGACGTCAACGAGTTCGACCAG
ClGarS1     CGCGATGTGGACCTGGGGGAACTGAGCAGTGACGTCATCTCGCACATGGAGAGCTTTGATGTGGCCGAGTTTGACCAG
DaRerHBTF1  GGTGCGGTGGACATCGGTGAGCTGAGCAGTGATGTCATCTCCAACATAGAGGCCTTCGATGTTAATGAGTTCGACCAG

        1015
HoSapSSDR3  TACCTGCCGCCCAACGGCCACCCGGGGGTGCCGGCCACG---------CACGGC---------CAGGTCACCTACACG
MuMusSCG1   TACTTGCCACCCAACGGCCACCCAGGGGTTCCGGCCACC---------CACGGC---------CAGGTCACCTACACT
CaLupS1     TACCTGCCGCCCAACGGGCACCCCGGGGTGCCGGCCACG---------CACGGC---------CAGGTCACCTACACG
GaGalSTF2   TACCTGCCCCCCAACGGCCACCCGGGGGTCCCGGCCACC---------CACGGCCAG------GTCACCACCTACAGC
CoJapSSDRY  TACCTGCCCCCCAACGGCCACCCGGGGGTCCCGGCCACC---------CACGGCCAA------GTCACCACCTACAGT
AlMisS1     TACCTCCCACCGAATGGGCACCCTGGAGTCCCAGCCACT---------CACGGGCAGCCTGGCCAGGTGACCTACACC
TrScrHCC    TACCTCCCACCCAACGGTCACCCTGGAGTCCCGGCCACT---------CATGGGCAACCCGGTCAAGTCACCTATAGT
LeOliS      TACCTCCCACCCAACGGTCACCCGGGAGTCCCGGCCACC---------CACGGCCAACCCGGTCAAGTCACCTACAGC
AnPlaSSDRY  TACCTCCCCCCCAACGGCCACCCGGGGGTCCCGGCCACC---------CACGGCCAACCCGGCCAGGTCACCTACACC
LaChaTFS9   TATCTGCCACCCAATGGCCACCCAGGTGTCCCTGTCACC---------CATGGCCAGAGTGGCCAGGTTACCTACACT
XeLaeTFS1   TACCTGCCGCCCAATGGCCACCCAGGGGTTGGCTCCACT------------------------CAGGCCTCGTACACA
BuBufTFSP   TACCTGCCACCCAATGGCCACCCAGGTGTTGGCTCCACA------------------------CAGGCCCCATACACA
XeSilTFS9   TACCTGCCACCCAATGGCCACCCAGGTGTTGGCTCCACA------------------------CAGGCCCCATACACA
BuGarS      TACCTGCCACCCAACGGCCACCCGGGAGTCGCCTCCACA------------------------CAGGTGACCTACACC
RhMarS      TACCTGCCACCCAACGGCCACCCGGGAGTCGCCTCCACA------------------------CAGGTGACCTACACC
GlRugSAA    TACCTGCCGCCCAACGGCCACCCCGGAGTGGCCTCCACG------------------------CAGGTGACCTACACG
PlWalS      TACCTTCCGCCCAACAGCCACCCTGGTGTGCCCACAACC---------CATGGGCAGGCTGGACCAGGTACCTATACC
ScCanS1     TACCTCCCACCCAACGGGCACCCAGGGGCCCCAGTCACCCACACGGGCCATGGACAGAGTGCTCAGGCCACCTACACC
DaRerHBTFS  TACCTCCCGCCGAACGGTCACCAGAACGCACCC---------------------------------------TACGCT
MoAlbHBTF1  TACCTCCCACCCAACGGGCACCCAGGCACCACG------------------------------CCAGTCAGCTACACG
OrNilTFS9a  TATCTCCCTCCCAACGGCCACCCGGGCTCCACC---------------------AACGCAGCGCCAGTAAGTTACACT
GaAcuTFS9a  TACCTTCCGCCCAACGGGCACCCCGGCTTAGCG---------------------GGCGCCGCC---------------
XiMacTFS9a  TACCTCCCGCCCAACGGCCACCCGGGGCCCGTC---------------------GGCGCCACTCCGGTCAGCTACAGC
TaRubTFS9b  TACCTCCCGCCTAACGGGCATCCCGGCTCTGCT---------------------AACGCCACCCCGGTCGCGTACAGT
TeNigTFS9a  TACCTCCCACCCAACGGGCATCCAGGCTCAGCC---------------------AACGCCACCCCAGTCAGCTATAGC
OrLatSCC1   TACCTCCCCCCCAACGGGCACCCTGGCGCCGCCCCTGGG---------------AGCACCGCCCCCGTCAGCTACAGC
AsMexTFS9a  TACCTTCCACCCAACGGGCATCCGGGCGCTGCC---------------------------------------TACGTG
GaMorS1     TACCTGCCCCCCAACGGCCACCCCGGCGGCGCT---------------------------------------GGGGTG
MoAlbHBTFS  TACCTGCCACCTCACAGCCATGCTGGGGTGACTGGC---------------------ACAGTTCAGACAGGCTATACC
OdBonTFS    TACTTGCCACCGCACAGCCACGCTGGGATGACAGGT---------------------CCAGCCCCAGCTGGCTACACT
PoRetS      TACCTGCCGCCTCACAGCCACGCTGGGGTCGCTGGT---------------------GCAGCCCAGGCTGGCTACACA
XiMacTFS9b  TACCTGCCACCTCACAGCCACGCTGGGGTTGCTGGT---------------------GCAGCCCAGGCTGGCTACACA
EpCoiS2     TACCTGCCACCTCACAGCCATGCTGGGGTGACCGGA---------------------GCGGCCCAGGCCGGCTACACC
TaRubTFS9a  TACCTCCCGCCTCACAGCCACGCCGGGGTGAGCGGC---------------------GCCCCGCAGGCCGGGTACACC
TeNigTFS9b  TACCTCCCTCCTCACAGCCACGCCGGGGTGAGCGGC---------------------GTCCCGCAGGCCGGGTACACC
OrNilTFS9b  TACCTGCCACCACACAGCCATGCTGGGGTGAATGGC---------------------GCAGCGCAGGCTGGCTACAGC
CiMonTFS9   TACCTGCCACCTCACAGCCATGCTGGGGTGAATGGT---------------------GCAGCACAGGTTGGCTATACC
CySemSHB    TACCTGCCACCTCACAGCCATGCTGGATTGCCCAGT---------------------GGAGCCCAGGCGGGCTACACC
OrLatSCC    TACCTGCCCCCTCACAGCCACGCTGGGATGACCGGC---------------------ACAGCCCAGACCAGCTACTCC
GaAcuTFS9b  TACCTGCCACCTCACAGCCACATTGGAGTGACGNGG---------------------AGCAGCTCAGGGCGCTACGCC
```

```
SaSalTFS2    TACCTGCCACCCCACGGGCACCCCGGCATGCCTGGCGTC---------------AACGGCGCCCAGACGGGCTACACG
GaMorS2      TACCTGCCGCCCCACAGCCACGCCCACGCCCAC-----------------------CTGACGGCCGGCGGCTACACG
AsMexTFS9b   TACCTGCCGCCGCACGGTCATCCGGGGCTTCCC-----------------------GCCAACGCTCAAGCCTACTCA
ClGarS       TACTTGCCGACGCAC-----------------------------------------GGGGCTCAGGTCTACTCCTCC
CyCarHTF13   TACCTGCCCCCTCGCGGACACCCGGGGGTCAGC-----------------------GGCGGCATGCAGGCGTATCCC
ClGarS1      TACCTGCCACCTAACGGGCACCCCGGGCACGGA--------------------------------------------
DaRerHBTF1   TACCTGCCTCCGCACGGGGCCCCGGGGCCGGCC-----------------------------GGTGCAGGGTTCTCC

         1093
HoSapSSDR3   GGCAGCTACGGCATCAGC------AGCACCGCGGCCACCCCGGCGAGC---GCGGGCCACGTGTGGATGTCCAAG---
MuMusSCG1    GGCAGTTACGGCATCAGC------AGCACCGCACCCACCCCTGCGACC---GCGGGCCACGTGTGGATGTCGAAG---
CaLupS1      GGCAGCTACGGCATCAGC------AGCACCGCGGCCACCCCGGCGGGC---GCGGGCCACGTGTGGATGTCCAAG---
GaGalSTF2    GGTACCTACGGCATCAGC------AGCTCGGCCAGCTCTCCGGCGGGC---GCCGGGCACGCCTGGATGGCCAAG---
CoJapSSDRY   GGTACCTACGGCATCAGC------AGCTCGGCCAGCTCTCCGGCAGGC---GCAGGGCATGCCTGGATGGCCGAA---
AlMisS1      GGCAGCTACGGGATCAGC------AGCACTGCCGCCACCCCGACCGGG---GCCGGGCACGTGTGGATGTCCAAG---
TrScrHCC     GGCAGCTACGGAATCAGT------AGCACGTCAGCCACTCAAGCTGGA---GCCGGGCCCGTTTGGATGTCCAAG---
LeOliS       GGCAGCTATGGGATCAGC------AGCACGCCTGCCACTCAAGCTGGA---GCCGGGCACGTTTGGATGTCCAAG---
AnPlaSSDRY   GGCAGCTACGGCATCAGC------AGCTCGGCCGGCTCACCGGCCGGT---GCCGGGCACGTCTGGATGTCCAAG---
LaChaTFS9    GGCAGTTATGGCATCAGC------AGTACAGCGGTCACTCAGGCAGGT---GGTAGCCATCCTTGGATCTCCAAG---
XeLaeTFS1    GGCAGTTATGGCATCAGC------AGCACCCCTAGTGCAACCACAGGT---GCTGGCCCTGCCTGGATGTCTAAA---
BuBufTFSP    GGCAGTTATGGCATCAAC------AGCACCCCCAGCGCTACTCCGGGT---GCTGGCCCTGCCTGGATGTCTAAA---
XeSilTFS9    GGCAGTTATGGCATCAAC------AGCACCCCCAGCGCTACTCCGGGT---GCTGGCCCTGCCTGGATGTCTAAA---
BuGarS       GGCAGTTACGGCATCACC------AATACAAACAGTGCTACCACGGGCGCCGCCGGGCACACATGGATGTCCAAA---
RhMarS       GGCAGTTACGGCATCACC------AATACAAATAGTGCCACCACCGGC---GCCGGGCACACATGGATGTCCAAA---
GlRugSAA     GGCAGCTACGGCATCAGC------AGCGCCGCCGGCGGTCCTGCCGGG---GCTGGGCACGCGTGGATGCCCAAG---
PlWalS       AACAGCTACGGGGCTGGC------AACACAGCAGCTCCCCAACCTGGGGCTCCAGCCCATGCCTGGCTGGCCAAA---
ScCanS1      GGCAGCTATGGGATTTGC------AGCACTTCAGTCACCCAG---------GCCGCCCACCCTTGGTTGTCCAAG---
DaRerHBTFS   GGAGGATACGCC------------------------------------------------GCCTGGATGACCAAA---
MoAlbHBTF1   GGCAGCTACAGCATCAGCAGC---AGTGCACCCGTCAGTCCACAGCCAGGAGGTGCCACAGCCTGGATGGCTAAG---
OrNilTFS9a   GGCAGCTACAGCATCAGCAGCGGCGGCGCTCCAGTCAGCCCCCAGTCAGGAGGC------GCCTGGATGGCTAAA---
GaAcuTFS9a   ---------------------------------------------------GCG------GCTTGGCTGGCGAAA---
XiMacTFS9a   GGCAGCTACAGCATAAGCGGC---GGGGCGCCGCTCAGCCCGCAGCCG-----------GCCTGGATGTCTAAA---
TaRubTFS9b   GGCACTTACAGCATCAGTAGC---AGCGGCCCTGTCAGCCCACAGACGGGAGGCGTGGCAGCCTGGCTGACTAAACCC
TeNigTFS9a   GGCACCTACAGCATCAGCAGC---AGTGGCCCCGTCAGCCCACAGGCGGGAGGCGTGGCAGCCTGGCTGACCAAACCC
OrLatSCC1    GGCAACTACAGCATCAGCGGC---GCCCCGCCTCTGAGCCCGCAGGCAGGCGGGGGGCCGGCCTGGATGGCTAAG---
AsMexTFS9a   GGCGGCTACAGCCTGGCCGGTCCCGGCACAGTTGGTCAGGCGGCGGCAGGAGGT------GTCTGGATGAGCAAG---
GaMorS1      GCCGCCTTCTGCCCGTCC------------------------------------------GCCTGGATGGGCAAG---
MoAlbHBTFS   AACAGCTAC---AACATCAGCAGCCCTTCAGTCGTCCAGGCAGCCAATGCTGGAGCCCACACCTGGGTGTCCAAG---
OdBonTFS     AACAGCTAC---GGTATCAACAGCTCCTCAGTCAGCCAGGCTACCAATGTTGGAGCCCACGCCTGGATGTCCAAG---
PoRetS       GGTAGCTAC---GGTATTAGCACCGCCTCAGTCGGTCAGGGAGCCGGTGTGGGAGCTCATGCTTGGATGTCCAAG---
XiMacTFS9b   GGCAGCTAC---GGTATTAGCACCTCCTCAGTCGGTCAGGGAGCCGGTGTGGGAGCTCATGCTTGGATGTCCAAG---
EpCoiS2      AGCAGCTAC---GGCATCAGCAGCTCCTCAGTCAGCCAGGCGGCCAATGTCGGAGCCCACACCTGGATGTCCAAA---
TaRubTFS9a   GGCAGTTAC---GGCATCAGCAGCTCCTCCGTGAGCCAGGCGGCCGGCGTGGGGGCCCAAGCCTGGATGTCCAAG---
TeNigTFS9b   GGCAGTTAC---GGCATCAGCGGCTCCTCCGTTAGCCAGACGGGCAGCGTTGGGACCCAAGCCTGGATGTCCAAG---
OrNilTFS9b   AGCGGCTAC---CCTATCAACAGCTCCTCTGTCGGCCAGCCAGCCAGTGTTGGAGCCCACGCCTGGATGTCCAAG---
CiMonTFS9    AGCAGCTAT---CCCATCAACAGTTCCTCAGTCGGCCAGCCACCCAGTGTTGGAGCCCACGCCTGGATGTCCAAA---
CySemSHB     GGCAGCTAT---GGCATGGGCAGCTCCTCTCTAGGCCAGGCAGCTAATGTTGGGGTCCATGCTTGGATGTCCAAG---
OrLatSCC     AACAACTAC---GTTATCAACAGCTCTGCCGTTGGCCAGACAGCCAATGTTGGAGCCCATGCCTGGATGCCCAAA---
GaAcuTFS9b   AGCGGCTAT---GGCATCGGCGGCTCCTCAGTCGGCCACGCGGCCAATGTCGGGGCCCACGCCTGGATGTCCAAG---
SaSalTFS2    AGCAGCTACCGCGGCATCAGCGCCAGCTCCATTGGCCAG---GTGGGTGCTGGAGGACATGGCTGGATGTCCAAG---
GaMorS2      GGGAGCTAC------------------------GGCCAGTCGGCTAGCCCGGGCGCCCACGCCTGGATGCTGAAG---
AsMexTFS9b   GCCAGCTAC---GGTCTGTCCCTCTCAGCT------------------------------GGTTGGATGTCCAAG---
ClGarS       TCTGGGTAT------------------GGCATGGGCCAGACAGCAGGCACCAGCGGGCATGGCTGGATGGGCAAA---
CyCarHTF13   GCCGGGTAC------------------------------------------GGTGGCACCTGGATGTCCAAA---
ClGarS1      ---------------------------------------TGGATGAGCTCAGCGGCCAGCGGTTGGATGACCAAG---
DaRerHBTF1   AGCGGGTAC------------------------------------------GGCAGCGCAGCCTGGATGCACAAA---

         1171
HoSapSSDR3   ---------------------------------------CAGCAGGCG---------CCGCCGCCACCCCCGCAGCAG
MuMusSCG1    ---------------------------------------CAGCAGGCG---------CCGCCCCCTCCTCCGCAGCAG
CaLupS1      ---------------------------------------CAGCAGGCGCCGCCGCCGCCGCCGCCGCCCCCGCAGCAG
GaGalSTF2    ---------------------------------------CAG---------------CAGCCGCAGCCCCCACAGCCC
CoJapSSDRY   ---------------------------------------CAG---------------CAGCCACAGCCCCCACAACCC
AlMisS1      ---------------------------------------CAGCCGCAGCCGCCGCAGCAGCCGCAGCCGCCGCAGCCG
TrScrHCC     ---------------------------------------CAACCG---CCGCAGCCCCAACAGCAGCCG------CCG
LeOliS       ---------------------------------------CAACAGCACAGCACAGCACGCCGCCGCCCC------AGG
AnPlaSSDRY   ---------------------------------------CAG---------------CAGCCTCAACCCCCACCACAG
LaChaTFS9    ---------------------------------------CAG---------------CAGCAGCAG------------
```

```
XeLaeTFS1    ---------------------------------------CAACAG------------CAACAG---------------
BuBufTFSP    ---------------------------------------CAACAA------------CAGCAGCAGCAGCAACAACCA
XeSilTFS9    ---------------------------------------CAACAA------------CAGCAGCAGCAGCAACAACCA
BuGarS       ---------------------------------------------------------CAGCAGCAGCCGCAGCAGCCG
RhMarS       ---------------------------------------CAGCAG------------CAGCAGCAGCCGCCCCAGCCG
GlRugSAA     ---------------------------------------CAGCCC------------CAACAGCAACCTCAACAGCCC
PlWalS       ---------------------------------------CAG---------------CAACAGCAG------------
ScCanS1      ---------------------------------------CAG------------------------------------
DaRerHBTFS   ------------------------------------------CCCCAAAACGGCAGCCCTCAA---------------
MoAlbHBTF1   ACTCAG---------------------------------AACCCACAGGGA---------CAG---------------
OrNilTFS9a   AGCCCG---------------------------------AACCAGCAGGGACAACAGCAGCAG---------------
GaAcuTFS9a   AGCCAA---------------------------------AACCAGCAGGGA------CAGCAG---------------
XiMacTFS9a   AGCCAG---------------------------------AACCAGCAGGGA---CAGCAGCAG---------------
TaRubTFS9b   AACCAG---------------------------------AACCAGCAGGGA---------------------------
TeNigTFS9a   AACCAG---------------------------------AACCAGCAGGGACAGCAGCAGCAG---------------
OrLatSCC1    GCCCAC---------------------------------AGTCAGCAG------------CAG---------------
AsMexTFS9a   ACCCAGAGTGGGAACCCTCAGGGTGGGCCACAGCCGTCAAATCAGCAGCAGCAACAGCAGCAG---------------
GaMorS1      ------------------------------------------GGCCAGAGCCTGCAGCAGCAG---------------
MoAlbHBTFS   ------------------------------------------CAGCAGCAGCAGCAGCAGCAG---------------
OdBonTFS     ---------------------------------------------------CAGCAGCAACAG---------------
PoRetS       ------------------------------------------------CAGCAGCAACAGCAG---------------
XiMacTFS9b   ------------------------------------------------------CAGCAGCAG---------------
EpCoiS2      ---------------------------------------------------CAGCAGCAGCAG---------------
TaRubTFS9a   ------------------------------------CAGCAGCAGCAGCAGCAGCAGCAGCAG---------------
TeNigTFS9b   ---------------------------------CAGCAGCAGCAGCAGCAGCAGCAGCAGCAG---------------
OrNilTFS9b   ---------------------------------CAGCAGCAGCAGCAGCAGCAGCAGCAACAG---------------
CiMonTFS9    ---------------------------------CAGCAACAGCAGCAACAGCAGCAC---------------------
CySemSHB     ---------------------------CAGCAGCAGCAGCAGCAACAGCAGCAGCAGCAGCAG---------------
OrLatSCC     ------------------------------------------------------------CAG---------------
GaAcuTFS9b   ---------------------------------------------CAGCAGCAGCAGCAGCAG---------------
SaSalTFS2    ------------------------------------------------------CAGCAGCAG---------------
GaMorS2      ---------CAGCATCAGCAGCAGCAGCAGCAGCAGCAGCAGCAGCAGCAGCAGCAGCAGCAG---------------
AsMexTFS9b   ---------CAGCATCAGCAACAACATCAGCAGCAGCAGCCACAGCAGCCGCCACAGTCGCAATCGCAGCCACAGCAG
ClGarS       ---------------------------------------------------CAACAGCCACAG---------------
CyCarHTF13   ------------------------------------------------------------------------------
ClGarS1      ------------------------------------------------------------------------------
DaRerHBTF1   ------------------------------------------------------------------------------

          1249
HoSapSSDR3   CCCCCACAGGCCCCGCCG---GCCCCGCAGGCGCCCCCGCAGCCGCAGGCGGCGCCCCCACAGCAGCCGGCGGCACCC
MuMusSCG1    CCTCCGCAGGCCCCGCAA---GCCCCACAGGCGCCTCCGCAG---CAGCAAGCACCCCCGCAGCAGCCGCAGGCA---
CaLupS1      TCCCCGCAGGCGCCCCCGCAGCCCCCGCAGGCGCCCCCGCAGGCCCCGCAGGCGCCCCCGCAGCCGCAGCCCGCGCCC
GaGalSTF2    CCAGCACAG------CCC------------------------------------------------------------
CoJapSSDRY   CCAGCACAG------CCC------------------------------------------------------------
AlMisS1      CCGCCGCAGGCTCAGCCC------------------------------------------------------------
TrScrHCC     CCGCCCCAGGCCCCGCCC------------------------------------------------------------
LeOliS       CCAGCGCAG---------------------------------------------------------------------
AnPlaSSDRY   CCCCCCCAG---CAGCCC------------------------------------------------------------
LaChaTFS9    ------------------------------------------------------------------------------
XeLaeTFS1    CAGCCTCAA---------------------------------------------------------------------
BuBufTFSP    CAGCCTCCC---------------------------------------------------------------------
XeSilTFS9    CAGCCTCCC---------------------------------------------------------------------
BuGarS       CCCCAGCAG---------------------------------------------------------------------
RhMarS       TCCCAGCAG---------------------------------------------------------------------
GlRugSAA     CAGCAGCAG---------------------------------------------------------------------
PlWalS       ------------------------------------------------------------------------------
ScCanS1      ------------------------------------------------------------------------------
DaRerHBTFS   ------AGC---------------------------------------------------------------------
MoAlbHBTF1   ------CAG---------------------------------------------------------------------
OrNilTFS9a   ------CAG---------------------------------------------------------------------
GaAcuTFS9a   ------CAG---------------------------------------------------------------------
XiMacTFS9a   ------CAG---------------------------------------------------------------------
TaRubTFS9b   ------CAG---------------------------------------------------------------------
TeNigTFS9a   ------CAG---------------------------------------------------------------------
OrLatSCC1    ------CAG---------------------------------------------------------------------
AsMexTFS9a   ------CAA---------------------------------------------------------------------
GaMorS1      ------AGG---------------------------------------------------------------------
MoAlbHBTFS   ------CAG---------------------------------------------------------------------
```

```
OdBonTFS    ------CAG---------------------------------------------------------------------
PoRetS      ------CAG---------------------------------------------------------------------
XiMacTFS9b  ------CAG---------------------------------------------------------------------
EpCoiS2     ------CAG---------------------------------------------------------------------
TaRubTFS9a  ------CAG---------------------------------------------------------------------
TeNigTFS9b  ------CAG---------------------------------------------------------------------
OrNilTFS9b  ------CAG---------------------------------------------------------------------
CiMonTFS9   ------------------------------------------------------------------------------
CySemSHB    ------CAG---------------------------------------------------------------------
OrLatSCC    ------CAG---------------------------------------------------------------------
GaAcuTFS9b  ------CAG---------------------------------------------------------------------
SaSalTFS2   ------CAG---------------------------------------------------------------------
GaMorS2     ------CAG---------------------------------------------------------------------
AsMexTFS9b  CCACCACAG---------------------------------------------------------------------
ClGarS      ------CAA---------------------------------------------------------------------
CyCarHTF13  ------CAG---------------------------------------------------------------------
ClGarS1     ------------------------------------------------------------------------------
DaRerHBTF1  ------------------------------------------------------------------------------

         1327
HoSapSSDR3  CCGCAGCAGCCACAGGCGCACACGCTGACCACGCTG------------------AGCAGCGAGCCGGGCCAGTCCCAG
MuMusSCG1   CCCCAGCAGCAGCAGGCACACACGCTCACCACGCTG------------------AGCAGCGAGCCAGGCCAGTCCCAG
CaLupS1     CCGCAGCCGCAGGCGGCGCACACGCTGACCCCGCTG------------------AGCAGCGAGCCGGGCCAGGCCCAG
GaGalSTF2   ---------CCGGCACAGCACACACTGCCAAGCACT------------------GAGCGTGAGCAGGGTCCGGCACAG
CoJapSSDRY  ---------CCGGCGCAGCACACACTGCCAGCACTG------------------AGCGGTGAGCAGGGCCCGACACAA
AlMisS1     ---------CAAGCGGCGCACACGATGACCCCCCTG------------------AGTGGCGAGCAGGGGCAGTCCCAG
TrScrHCC    ---------CAGCCGCCGCATACCATGACCACCCTG------------------AGCAGCGAACAGGGCCAGTCCCAG
LeOliS      ------------------CATGCGATGACCACCCTG------------------AGCAGCGAACAGGGCCAGTCCCAG
AnPlaSSDRY  ---------CCGGCGCAGCACGGGTTGCCGGCGCTG------------------AGCAGCGAGCAGGGCCAGGCGCAG
LaChaTFS9   ------------------CACTCAATAACCACCCTG------------------AGCAGCGAACAAGGGCAGGCCCAG
XeLaeTFS1   ---------------CAACATTCACTGTCAACCCTA------------------AACAGCGAGCAAAGCCAGTCCCAG
BuBufTFSP   ---------------CAACACTCACTGTCAACCATA------------------AACAGCGAGCAAAGCCAGTCCCAG
XeSilTFS9   ---------------CAACACTCACTGTCAACCATA------------------AACAGCGAGCAAAGCCAGTCCCAG
BuGarS      ---------------CCGCACACATTGTCGACTCTG------------------AGCAGCGAGCAGAGCCAGTCCCAG
RhMarS      ---------------CCGCACGCATTGTCCACTCTG------------------AGCGGCGAGCAGAGCCAGTCCCAG
GlRugSAA    ---------------CAGCACGGTTTACCGACTCTG------------------AGCAACGAGCAGAGCCAAGCCCAA
PlWalS      ------------------CACACGTTGACCACACTG------------------GGCAGCGAGCAGAGCCAGGCGCAG
ScCanS1     ------------------CATTCGATGACGGGCCTC------------------ACCAGCGAGCAGGGACAG---CAG
DaRerHBTFS  ------------------AGCCAGCTGACCCCGCTG---------------------AACCCCGCAGAACCCGACCAG
MoAlbHBTF1  ------------------CAGACTCTGACCACTCTG------GGGAGCAGTGGTGGCTCAGAGGCAGCTTCTGCCCAG
OrNilTFS9a  ------------------CACACCCTGACCACCCTG------GGGAGCAGCGGGGCTTCAGACGCCGCTCAGACCCAG
GaAcuTFS9a  ------------------CATACTCTGACCCCACTG------GGGGGAGGGGGCGCC------------------GAG
XiMacTFS9a  ------------------ATCACCCTCACCACGTTG---GGCGGCGGCGGCGGAGGCTCGGACGCTGCCCAGGCGCAG
TaRubTFS9b  ------------------CACACCCTGACCACTCTG---GTGGGCAGCAGC---------------ACAGAAGCCCAG
TeNigTFS9a  ------------------CACACCCTGACCACTCTG---GTGGGCAGCGGC---------------TCAGAAGCCCAG
OrLatSCC1   ------------------CACAGCCTGACCCCCCTG------GGGACCAGCGGGGGTTCAGAGGCCGCC------CTG
AsMexTFS9a  ------------------CATTCCCTGACCCAGCTG------GGTACTGGCAATGGTGGGGATCAGGGTCAGCAGAGG
GaMorS1     ------------------------------------------------------------------------------
MoAlbHBTFS  ------------------CACTCTCTGACCACCCTG---GGTGGAGGAGGAGAGCAAGGCCAGCAGGGTCAACAG---
OdBonTFS    ------------------CATTCTTTGACCACCCTG---GGTGGAGCAGGAGAGCAAGGCCAGCAGGGTCAGCAG---
PoRetS      ------------------CATACTCTGACCACCCTT---GGTGGAGCAGGAGAGCAAGGTCAACAGGGCCAGCAG---
XiMacTFS9b  ------------------CATACTCTGACCACCCTT---GGTGGAGCAGGAGAGCAAGGTCAACAGGGCCAGCAG---
EpCoiS2     ------------------CACTCTCTGACCACCCTG---GGTGGAGGAGGAGAGCCAGTCCAGCAGGGTCAACAG---
TaRubTFS9a  ------------------CACTCCTTGACTGCCCTG---AGTGGGGGAGGAGAGCAAAGCCAGCAGGGTCAGCAG---
TeNigTFS9b  ------------------CACCCCCTGACCACCCTG---GGTGGGGGAGGAGAGCAAAGCCAGCAGGGTCAGCAG---
OrNilTFS9b  ------------------CTCTCTCTGACCACTCTG---GGTGGAGGAGGAGAGCAAAGCCAGCAGGGTCAACAG---
CiMonTFS9   ---------------------TCTCTGACCACTCTG---GGTGGAGGAGGAGAGCAAGGCCAACAGGGTCAACAG---
CySemSHB    ------------------CACTCTCTGACCACACTG---GGCGGAGGAGGTGAGCAAGGTCAGCAAGGTCAACAA---
OrLatSCC    ------------------CATTCTTTGGCCACCCTC---GGTGGGGGTGGAGATCAAAGCCAACAGGGTCAACAG---
GaAcuTFS9b  ------------------CACTCTTTGACCACCCTG---GGCGGAGGAGGAGAGCAAGGCCAGCAGGGTCAACAG---
SaSalTFS2   ------------------CACTCCATCTCCGCCCTGAGTGGAGGTGGAGGTATTGGGGGAGAGCAAGGCCAGAGCCAG
GaMorS2     ------------------CACTCTCTGACCACGCTG---GGCGGAGACGGCGAGCAG---------------------
AsMexTFS9b  ------------------CACTCCCTGACCACGCTGACCCCGGCAGACCAGAGCCAGTCCCAACAA------------
ClGarS      ------------------CACTCTCTGACCACACTT---GGCACAGCTGGCGAGCAGGGGCAGACCCAACAG------
CyCarHTF13  ------------------CACTCGATGGCCAGC------GGTGGCGAGCAGAGCCAAGGCCAGCAG------------
ClGarS1     ---------------------AGCCCCAGTTCCAGCCCTCAGGTGGGTGCTACCAGCCCTGGTGAGGATCAGAGCCAG
DaRerHBTF1  ------------------------------CCACTC---GCGAGCAGCTCCATGGCTAATGCCGGCGAGCAGCACCAG
```

```
        1405
HoSapSSDR3   ---CGA---ACGCACATCAAGACGGAGCAGCTGAGCCCCAGCCACTACAGC---------------------------
MuMusSCG1    ---CGA---ACGCACATCAAGACGGAGCAGCTGAGCCCCAGCCACTACAGC---------------------------
CaLupS1      ---CGA---ACGCACATCAAGACGGAGCAGCTGAGCCCCAGCCACTACAGC---------------------------
GaGalSTF2    CAGCGG---CCGCACATCAAAACAGAGCAGCTGAGCCCCAGCCATAACAGC---------------------------
CoJapSSDRY   CAGCGG---CCGCACATCAAAACGGAGCAGCTGAGCCCCAGCCACTACAGC---------------------------
AlMisS1      CAGCGG---CCGCACATCAAGACGGAGCAGCTGAGCCCCAGCCATTACAGC---------------------------
TrScrHCC     CAGAGG---ACACACATCAAGACAGAACAACTCAGTCCGAGCCATTACAGC---------------------------
LeOliS       CAGAGG---ACACACATCAAGACAGAGCAGCTCAGTCCCAGCCATTACAGC---------------------------
AnPlaSSDRY   CAGCGG---ACGCACATCAAGACGGAGCAGCTGAGCCCCAGCCACTACAGC---------------------------
LaChaTFS9    CCCAGG---ACACACATCAAGACTGAGCAGCTAAGCCCAAGTCACTACAGC---------------------------
XeLaeTFS1    CAAAGG---ACACACATCAAGACCGAGCAACTGAGTCCAAGTCATTACAGTGAC------------------------
BuBufTFSP    CAAAGG---ACACACATCAAGACTGAACAACTGAGCCCTAGCCATTACAGTGAC------------------------
XeSilTFS9    CAAAGG---ACACACATCAAGACTGAACAACTGAGCCCTAGCCATTACAGTGAC------------------------
BuGarS       CAGAGG---ACACACATCAAGACCGAGCAGCTTAGTCCAAGTCATTACAGCGAC------------------------
RhMarS       CAGAGG---ACACACATCAAGACTGAGCAGCTTAGTCCAAGTCATTACAGCGAC------------------------
GlRugSAA     CAGAGG---ACACACATCAAAACCGAGCAGCTCAGCCCGAGTCACTACAGCGAC------------------------
PlWalS       CAGAGG---ACACACATCAAGACCGAACAACTTAGCCCCACACACTACAGC---------------------------
ScCanS1      CAGAGG---ACACACATCAAGACCGAGCAGTTGAGCCCCAGCCATTACAAT---------------------------
DaRerHBTFS   CCCCGAACGACGCATATTAAAACCGAACAGCTCAGCCCGAGCCATTACAACGAG------------------------
MoAlbHBTF1   CACAGG---ACCCAGATCAAGACAGAGCAACTGAGCCCAAGTCACTACAGCGAG------------------------
OrNilTFS9a   CATAGG---ACCCAGATCAAGACGGAGCAGCTGAGCCCGAGCCACTACAGCGAG------------------------
GaAcuTFS9a   CACCGG---ACCCAGATAAAGACTGAGCAGCTGAGCCCGAGCCACTACACCGAG------------------------
XiMacTFS9a   CACAGG---ACCCAGATCAAGACGGAGCAGCTGAGCCCGAGTCACTACAGCGAG------------------------
TaRubTFS9b   CACAGG---ACCCAGATCAAGACAGAACAGCTGAGCCCGAGCCATTACAACGAC------------------------
TeNigTFS9a   CACAGG---ACCCAGATCAAGACGGAACAGCTGAGCCCGAGCCATTACAACGAT------------------------
OrLatSCC1    CGCAGG---ACCCAGATTAAGACGGAGCAGCTGAGCCCGAGTCACTACAGCGAG------------------------
AsMexTFS9a   ACCCCG---ACACACATCAAGACAGAGCAGCTGAGTCCCAGCCACTACAGTGAG------------------------
GaMorS1      ---------ACCCACATCAAGACGGAGCAGCTGAGCCCGGGTCACTACTCGGAGCAGCTCAGCCCCGGGCACTACTCT
MoAlbHBTFS   ---AGAACCGCCCAGATCAAGACAGAACAGCTAAGTCCCAGCCACTTCAGCGAA------------------------
OdBonTFS     ---AGAACCACCCTGATTAAGACAGAGCAGCTGAGCCCAAGCCACTACAGCGAG------------------------
PoRetS       ---AGAACCACCCAGATCAAGACAGAGCAGCTCAGCCCGAGCCACTACAGTGAC------------------------
XiMacTFS9b   ---AGAACCACCCAGATCAAGACGGAGCAGCTCAGCCCGAGCCACTACAGTGAC------------------------
EpCoiS2      ---AGAACCACCCAGATCAAAACAGAGCAGCTGAGCCCCAGCCACTACAGCGAG------------------------
TaRubTFS9a   ---AGGCCCGCCCAGATCAAGACAGAGCAGCTGAGTCCGAGCCACTACAGCGAG------------------------
TeNigTFS9b   ---AGGCCCGCCCAGATCAAGACGGAGCAGCTGAGTCCGAGCCACTACAGCGAG------------------------
OrNilTFS9b   ---AGAACCACCCAGATTAAGACGGAGCAGCTGAGCCCCAGCCACTACAGCGAG------------------------
CiMonTFS9    ---AGAACCACCCAGATTAAAACAGAGCAGCTGAGCCCCAGCCACTACAGCGAG------------------------
CySemSHB     ---AGAACCACACAGATCAAGACAGAGCAGCTGAGTCCCAGTCACTACAGTGAG------------------------
OrLatSCC     ---AGAACCACTCAGATCAAGACGGAGCAGCTGAGCCCCAGCCACTACAGCGAG------------------------
GaAcuTFS9b   ---AGAACCACCCAGATCAAGACGGAGCAGCTGAGCCCCAGCCACTACAGCGAG------------------------
SaSalTFS2    GGGAGAACCACTCACATCAAGACGGAGCAGCTGAGCCCCAGCCACTACAGTGAG------------------------
GaMorS2      ---AGGACCACCCAGATCAAGACGGAGCAACTGAGCCCCAGCCACTACCGCGAG------------------------
AsMexTFS9b   ---CGCCCGACACAGATCAAGACAGAGCAGCTGAGCCCCAGCCACTACAGCGAC------------------------
ClGarS       ---CGGCCAGCACACATCAAGACAGAGCAGCTCAGCCCAAGCCACTACAGTGAG------------------------
CyCarHTF13   ---CGG---ACGCAGATCAAGACGGAGCGGCTGAGCCCCAGCCACTACAGCGAG------------------------
ClGarS1      CAGAGAACAACGCACATCAAAACAGAGCAGCTGAGTCCTAGCCAT---------------------------------
DaRerHBTF1   CAGAGG---GCGCAGATCAAGACGGAGCAGCTGAGCCCGGGCCACTACAGC---------------------------

        1483
HoSapSSDR3   ---------------------------------GAGCAGCAGCAGCACTCG---CCC---------------------
MuMusSCG1    ---------------------------------GAGCAGCAGCAGCACTCC---CCG---------------------
CaLupS1      ---------------------------------GAGCAGCAGCAGCACTCG---CCG---------------------
GaGalSTF2    ---------------------------------GAGCAGCAGCAGCATCCC---------GAGCAGCAGCAGCAGCAG
CoJapSSDRY   ---------------------------------GAGCAGCAGCAGCACTCC---CCGCAACAACAGCAGCAGCAGCAG
AlMisS1      ---------------------------------GAGCAGCAGCAGCATTCG---CCG---------------------
TrScrHCC     ---------------------------------GAGCAGCAGCAGCACTCC---CCT---------------------
LeOliS       ---------------------------------GAGCAACAGCAGCACTCC---CCA---------------------
AnPlaSSDRY   ---------------------------------GAGCAGCAGCAGCACTCG---CCG---------------------
LaChaTFS9    ---------------------------------GAG---CAGCAGCATTCG---CCC---------------------
XeLaeTFS1    ---------------------------------CAACAGCAACAGCATTCC---CCC---------------------
BuBufTFSP    ---------------------------------CAGCAGCAACAGCACTCC---CCC---------------------
XeSilTFS9    ---------------------------------CAGCAGCAACAGCACTCC---CCC---------------------
BuGarS       ---------------------------------CAGCAGCAGCAACACTCT---CCG---------------------
RhMarS       ---------------------------------CAGCAGCAGCAACACTCC---CCA---------------------
GlRugSAA     ---------------------------------CAGCAGCAACAGCATTCC---CCG---------------------
PlWalS       ---------------------------------GAGCAGCAGCAGCACTCC---CCC---------------------
ScCanS1      ------------------------------------GAGCAGCAGCACTCA---CCC---------------------
```

```
DaRerHBTFS   ---------------------------------CAG------CAGGGCTCT---CCG---------------------
MoAlbHBTF1   ---------------------------------CAG------CAGGGCTCC---CCG---------------------
OrNilTFS9a   ---------------------------------CAG------CAGGGCTCC---CCG---------------------
GaAcuTFS9a   ---------------------------------CAG------CAGGGTTCC---CCG---------------------
XiMacTFS9a   ---------------------------------CAG------CAGAGTTCC---CCG---------------------
TaRubTFS9b   ---------------------------------CAG------CAGAGCTCG---CCG---------------------
TeNigTFS9a   ---------------------------------CAG------CAGGGCTCG---CCG---------------------
OrLatSCC1    ---------------------------------CAG------CAGGGCTCC---CCC---------------------
AsMexTFS9a   ---------------------------------CAG------CAGGGCTCG---CCG---------------------
GaMorS1      GAGCAGCAGCAGCAGCAGCAGCAGCAGCAGCAGCAG------CAGAGCTCC---CCC---------------------
MoAlbHBTFS   ---------------------------------CAA------CAGGGCTCC---CCC---------------------
OdBonTFS     ---------------------------------CAG------CAGGGCTCC---CCA---------------------
PoRetS       ---------------------------------CAA------CAGGGATCC---CCG---------------------
XiMacTFS9b   ---------------------------------CAA------CAGGGATCC---CCG---------------------
EpCoiS2      ---------------------------------CAG------CAGGGCTCC---CCA---------------------
TaRubTFS9a   ---------------------------------CAG------CAGGGCTCC---CCC---------------------
TeNigTFS9b   ---------------------------------CAG------CAGAGCTCC---CCG---------------------
OrNilTFS9b   ---------------------------------CAG------CAGGGCTCC---CCA---------------------
CiMonTFS9    ---------------------------------CAG------CAGGGCTCC---CCA---------------------
CySemSHB     ---------------------------------CAG------CAGGGCTCT---CCT---------------------
OrLatSCC     ---------------------------------CAG------CAAAGCTCC---CCA---------------------
GaAcuTFS9b   ---------------------------------CAG------CAGGGCTCC---CCG---------------------
SaSalTFS2    ---------------------------------CAG------CAGAGCTCCCCTCCC---------------------
GaMorS2      ---------------------------------CAG------CAGGGCTCGCCTCCG---------------------
AsMexTFS9b   ---------------------------------CAAGGC---CAGGGTTCT---CCT---------------------
ClGarS       ---------------------------------CAGAGC---CAGGGCTCA---CCC---------------------
CyCarHTF13   ---------------------------------CAGCAGCGGCAGGGCTCT---CCG---------------------
ClGarS1      ------------------------------------------------------------------------------
DaRerHBTF1   ------------------------------------------CAGCAGCCG---CCG---------------------

          1561
HoSapSSDR3   ---CAACAGATCGCC---TACAGCCCCTTCAACCTCCCACACTACAGCCCCTCC------------------------
MuMusSCG1    ---CAACAGATCTCC---TACAGCCCCTTCAACCTTCCTCACTACAGCCCCTCC------------------------
CaLupS1      ---CAGCAGATCGCC---TACAGCCCCTTCAGCCTCCCGCACTACAGCCCGTCC------------------------
GaGalSTF2    CAACAGCAGCTGGGC---TACGGCTCCTTCAACCTGCAGCACTACGGCTTCTCG------------------------
CoJapSSDRY   CAACAGCAGCTGGGC---TACGGCTCCTTCAACCTGCAGCACTACGGCTCCTCG------------------------
AlMisS1      ---CAGCAGATCAAC---TACAGCTCCTTCAACCTCCAGCACTACAGTTCATCC------------------------
TrScrHCC     ---CAGCAGCTCAAT---TATAGCTCCTTCAACCCCCAGCACTACAGTTCCTCC------------------------
LeOliS       ---CAGCAGCTCAAT---TATAGCTCCTTCAACTTCCAGCACTACAGTTCCTCC------------------------
AnPlaSSDRY   ---CAGCAGCTCAAC---TACAGCTCCTTCAACCTGCAGCACTACGGCTCCTCC------------------------
LaChaTFS9    ---CAGCACATCAAC---TACGCCTCCTTCAACCTTCAACACTACGGCTCCTCT------------------------
XeLaeTFS1    ---CAGCAGCTGAAC---TACAGCTCCTTCAACCTGCAGCATTACAGCTCTTCA------------------------
BuBufTFSP    ---CAGCAGCTGAAC---TACAGCTCCTTCAACCTGCAGCATTACAGCTCTTCC------------------------
XeSilTFS9    ---CAGCAGCTGAAC---TACAGCTCCTTCAACCTGCAGCATTACAGCTCTTCC------------------------
BuGarS       ---CAGCAGCTCAGC---TACAGCTCCTTCAACCTCAACCATTACGGCTCTTCC------------------------
RhMarS       ---CAGCAGCTCACC---TACAGCTCCTTCAACCTCAACCATTACGGCTCTTCC------------------------
GlRugSAA     ---CAGCAGCTCAAC---TACACCTCCTTCAACCTCCAGCATTACGGGTCCACC------------------------
PlWalS       ---CAGCAGCTGAGC---TACAGCCCGTTCAACCTGCAGCACTACAACTCTACC------------------------
ScCanS1      ---CAGCAGATCAAC---TACGGCTCCTTCAATATGCAACACTACAGTTCATCT------------------------
DaRerHBTFS   ---CAGCACATCAGC---TACGGTTCCTTCAACGTTCAGCATCTCCAGCATTACAGC---------------------
MoAlbHBTF1   ---CAGCACGTCGCC---TACAGCCCCTTCAACCTGCAGCACTACAGCTCCCCCTCC---------------------
OrNilTFS9a   ---CAGCACGTCAGCCCCTACAGCCCCTTCAACCTGCAGCACTACAGC---CCCTCC---------------------
GaAcuTFS9a   ---CAACACGTCGCCTACAACAGCCCCTTCAACCTGCAGCACTACAGCCCCCCCTCC---------------------
XiMacTFS9a   ---CAGCACGTCCCC---TACAGCCCCTTCAACATCCAGCACTACAGCCCCCCGTCC---------------------
TaRubTFS9b   ---CAGCACGTCACC---TACAGCCCCTTCAATCTGCAGCACTACAGCCCCCCTTCC---------------------
TeNigTFS9a   ---CAGCACATCACC---TACAGCCCCTTCAATCTGCAGCACTACAGCCCCCCGTCC---------------------
OrLatSCC1    ---CAGAACGCCCCC---TACAGCCCCTTCAACCTGCAGCACTACAGCCCCCCCTCC---------------------
AsMexTFS9a   ---CAACACGTCACC---TACGGTTCCTTCAACCTCCAGCACTACACC------GCC---------------------
GaMorS1      ---CAGCACCTGTCC---TACGGCTCCTTCAACCTGCAGCACTACAGCAGCTCCTCCGGGGGGTCCGTCAACGCCCCC
MoAlbHBTFS   ---CAGCATGTCACC---TATGGGTCCTTCAACCTGCAGCACTACAGCACCTCCTCT---------------------
OdBonTFS     ---CAACATGTCACC---TATGGCTCCTTCAACCTGCAGCACTACAGCGCCTCCTCT---------------------
PoRetS       ---CAGCACATCACC---TATGGCTCATTCAACCTACAGCACTACAGCCCCTCTTCT---------------------
XiMacTFS9b   ---CAGCACATCACC---TATGGCTCATTCAACCTACAGCACTACAGCCCCTCTTCT---------------------
EpCoiS2      ---CAGCATGCCACC---TATGGGTCCTTCAACCTGCAGCACTACAGCACCTCCTCT---------------------
TaRubTFS9a   ---CAGCACGTCACA---TACGGGTCCTTCAACCTGCAACACTACAGCACCTCCTCC---------------------
TeNigTFS9b   ---CAGCACGTCACC---TACGGCTCCTTCAACCTGCAGCACTACAGCGCCGCCTCC---------------------
OrNilTFS9b   ---CAGCATGTCACC---TATGGGTCTTTCAACCTCCAGCACTACAGCACCAATTCT---------------------
CiMonTFS9    ---CAGCATGTCACC---TATGGGTCCTTCAACCTACAGCACTACAGCACCAACTCT---------------------
```

```
CySemSHB    ---CAGCACATCACC---TACGGCTCCTTCAACCTGCAGCACTACAGCACCTCCTCC---------------------
OrLatSCC    ---CAGCACGTCAGC---TACGGGTCCTTCAACCTGCAGCACTACAGCACCTCTTCT---------------------
GaAcuTFS9b  ---CAGCACGTCACC---TATGGGTCCTTCAACCTGCAGCACTACAGCGCCTCCTCC---------------------
SaSalTFS2   ---CAGCATGTCACC---TATGGTTCATTCAACCTGCAGCACTACAGCGCCTCATCC---------------------
GaMorS2     ---CAGCACGTGGCC---TACGGCTCCTTCAACCTGCAGCACTACAGCCCGGCCTCC---------------------
AsMexTFS9b  ---CAACACGTAGCG---TACGGTTCCTTCAACCTGCAGCACTACAGCACCTCGTCC---------------------
ClGarS      ---CAGCATGTGGCA---TATGGTTCCTTCAATCTACAGCATTACACTGCCACCTCC---------------------
CyCarHTF13  ---CAGCATGTGGCC---TACGGCTGCTTCAACCTGCAGCACTACAGCAGCAGCGGC---------------------
ClGarS1     ------------------TATGGCTCCTTCAACCTGCAGCACTACACCACGTCCACT---------------------
DaRerHBTF1  ---CAGCAG---------------------------CAGTTCTACAGCGCCCCC------------------------

         1639
HoSapSSDR3  ---------------------------------------------TACCCG---CCCATCACCCGC------TCA---
MuMusSCG1   ---------------------------------------------TACCCG---CCCATCACCCGC------TCG---
CaLupS1     ---------------------------------------------TACCCG---CCCATCACCCGC------TCG---
GaGalSTF2   ---------------------------------------------TACCCC---CCCATCACCCGC------TCG---
CoJapSSDRY  ---------------------------------------------TACCCC---CCCATCACCCGC------TCG---
AlMisS1     ---------------------------------------------TACCCA---ACCATCACCCGC------TCG---
TrScrHCC    ---------------------------------------------TACCCG---ACCATCACCCGC------TCG---
LeOliS      ---------------------------------------------TACCCG---ACCATCACCCGC------TCG---
AnPlaSSDRY  ---------------------------------------------TACCCC---CCCATCACCCGC------TCC---
LaChaTFS9   ---------------------------------------------TACCCA---ACCATCACCCGT------TCC---
XeLaeTFS1   ---------------------------------------------TACCCA---ACTATCACCCGT------GCA---
BuBufTFSP   ---------------------------------------------TACCCA---ACCATTACCCGT------GCC---
XeSilTFS9   ---------------------------------------------TACCCA---ACCATTACCCGT------GCC---
BuGarS      ---------------------------------------------TACCCG---ACCATCACACGC------TCA---
RhMarS      ---------------------------------------------TACCCG---ACCATCACACGC------TCA---
GlRugSAA    ---------------------------------------------TACCCA---ACCATCACCCGC------TCA---
PlWalS      ---------------------------------------------TACCCT---ACTATTACACGT------GCG---
ScCanS1     ---------------------------------------------TACCCT---TCCATTCCTCGC------ACC---
DaRerHBTFS  ---------------------------------------ACTTCATTTCCC---TCCATCACCAGA------GCG---
MoAlbHBTF1  ---------------------------------------TCC---TACCCA---GCCATCTCCAGG------GCG---
OrNilTFS9a  ---------------------------------------TCTTCCTACCCG---GCCATCTCCAGG------GCACAG
GaAcuTFS9a  ---------------------------------------TCCGCCTACCCGGCCGCCATCTCCAGA------GCG---
XiMacTFS9a  ---------------------------------------ACC---TACCCG---GCCATCTCCAGG------CCG---
TaRubTFS9b  ---------------------------------------CCT---TACCCC---GGCATCTCCAGA------GCC---
TeNigTFS9a  ---------------------------------------CCT---TACCCG---GCCATCTCCAGG------GCC---
OrLatSCC1   ---------------------------------------TCCTCGTACCCG---CCCATCTCCCGA------GCG---
AsMexTFS9a  ---------------------------------------TCCTCCTTCCCC---TCCATCAGCCGG------GCG---
GaMorS1     GCCCTCTCCTCCGCCTCCGTGTCGGCCGCCGCGGCCTCCTCCGTCTACCCG---ACGACGACGCGG------ACG---
MoAlbHBTFS  ---------------------------------------------TACCCC---TCCATCACAAGA------GCA---
OdBonTFS    ---------------------------------------------TACCCC---TCCATCACAAGA------GCA---
PoRetS      ---------------------------------------------TATCCG---TCCATCACAAGA------GCA---
XiMacTFS9b  ---------------------------------------------TATCCG---TCTATCACAAGA------GCA---
EpCoiS2     ---------------------------------------------TACCCC---TCCATCACAAGA------GCA---
TaRubTFS9a  ---------------------------------------------TACCCC---TCCATGACCAGA------GCG---
TeNigTFS9b  ---------------------------------------------TACCCC---TCCATG---AGA------GCG---
OrNilTFS9b  ---------------------------------------------TACCCC---TCCATCACCAGA------TCA---
CiMonTFS9   ---------------------------------------------TACCCC---TCCATTACCAGA------TCA---
CySemSHB    ---------------------------------------------TACCCC---TCCATCACCAGA------GCA---
OrLatSCC    ---------------------------------------------TACCCC---TCCATCACAAGA------GCA---
GaAcuTFS9b  ---------------------------------------------TACCCC---TCGATCACGAGA------GCA---
SaSalTFS2   ---------------------------------------------TACCCC---TCCATTACCCGC------GCC---
GaMorS2     ---------------------------------------------TACCCC---TCCATCACCCGCGGGCCGTCC---
AsMexTFS9b  ---------------------------------------------TACCCG---TCGGTCGCACGC------ACC---
ClGarS      ---------------------------------------TCCACATACCCA---TCCATTGCTCGT------ACT---
CyCarHTF13  ---------------------------------------------GGCAGC---AGTGGCAGCGGC------TCG---
ClGarS1     ---------------------------------TCGTCCTCTGCCTTCCCATCATGCATCAGCCGG------CCT---
DaRerHBTF1  ---------------------------------------------TACAGC------------CGA------GCG---

         1717
HoSapSSDR3  ------CAGTAC---GACTACACC---GAC---CACCAGAACTCCAGC------------------------------
MuMusSCG1   ------CAATAC---GACTACGCT---GAC---CATCAGAACTCCGGC------------------------------
CaLupS1     ------CAGTAC---GACTACACT---GAC---CACCAGAACTCCGGC------------------------------
GaGalSTF2   ------GAGTAC---GATTACACC---GAG---CACCAGAACTCCGGC------------------------------
CoJapSSDRY  ------CAGTAT---GATTACACC---GAG---CACCAGAACTCCGGC------------------------------
AlMisS1     ------CAGTAC---GACTACACA---GAC---CACCAGAGCTCCAAC------------------------------
TrScrHCC    ------CAGTAC---GACTACACA---GAC---CACCAGAGTTCCAAC------------------------------
```

```
LeOliS       ------CAGTAT---GACTACACA---GAC---CACCAGAGTTCCAAC------------------------------
AnPlaSSDRY   ------CAGTAT---GATTACGGC---GAG---CACCAGAACTCCGGT------------------------------
LaChaTFS9    ------CAGTAC---GACTACACA---GAC---CACCAGAGCACCAAC------------------------------
XeLaeTFS1    ------CAGTAT---GACTACACC---GAG---CACCAGGGTTCTAGT------------------------------
BuBufTFSP    ------CAGTAT---GACTACACA---GAG---CACCAAGGCTCCAAC------------------------------
XeSilTFS9    ------CAGTAT---GACTACACA---GAG---CACCAAGGCTCCAAC------------------------------
BuGarS       ------CAGTAC---GACTACACC---GAG---CACCAAGGTTCGAAC------------------------------
RhMarS       ------CAGTAC---GACTACACC---GAG---CACCAAGGTTCGAAC------------------------------
GlRugSAA     ------CAGTAC---GACTACACC---GAG---CACCAAGGCTCAAAC------------------------------
PlWalS       ------CAGTAT---GACTACACC---GAC---CACCAGAGCTCTAAC------------------------------
ScCanS1      ------CAGTAC---GAATATTCA---GAC---CACCAGAGTAGCAAT------------------------------
DaRerHBTFS   ------CAGTAC---GACTATTCC---GACAGCCACCAGGGCGGCGCC---------------------AGC------
MoAlbHBTF1   ---CAGCAGTAC---GACTACTCC---GAC---CATCATGGGGGCAGC---AGC---------ACCACCGCC------
OrNilTFS9a   CAGCAGCAGTAC---GACTACCCC---GAC---CACCAGGGGGGAGGCACTGCA---------ACTGCCTCC------
GaAcuTFS9a   ---CAGCAGTAC---GACTACTCC---GAC---CACCAAGGGGGCGGG------------------GCCGCC------
XiMacTFS9a   ---CAGCAGTACTCCGAATACTCT---GAG---CACCAGGGGGGAGGC---------------------GCC------
TaRubTFS9b   ---CAACAGTAC---GACTTCTCT---GAG---CACCAGGCAGCGAACAACAGC------GGTACGAATGCC------
TeNigTFS9a   ---CAACAGTAC---GACTACTCC---GAG---CACCAGGGAGCAAACAACAGTGGCGGCGGCACAAACACC------
OrLatSCC1    ---CAGCAGTAT---GATTACCCC---GAC---CCCCAGGGAGGGGGC------------------------------
AsMexTFS9a   ------CAGTAC---GACTACGGT---GAC---CAGCAGGGAGCCGCC---------------ACCGCCGCC------
GaMorS1      ------CAGTAC---GACTACTCGGTGGACCCCCAGCAAGGGGGCGGC---------------GCCGGCCCC------
MoAlbHBTFS   ------CAGTAT---GACTACTCA---GAC---CACCAAGGTGGTGCCAAC---------------------------
OdBonTFS     ------CAGTAT---GACTATTCA---GAC---CACCAAAGTAGCGCCAAC---------------------------
PoRetS       ------CAGTGT---GACTATTCA---GAA---CACCAGAGCAGTGCCAAC---------------------------
XiMacTFS9b   ------CAGTAT---GACTATTCA---GAA---CACCAGAGCAGTGCCAAC---------------------------
EpCoiS2      ------CAGTAT---GACTATTCA---GAC---CACCAAGGTGGTGCCAAC---------------------------
TaRubTFS9a   ------CAGTAT---GACTATTCA---GAC---CACCAAGGTGGAGCCAAC---------------------------
TeNigTFS9b   ------CAGTAC---GATTATTCA---GAC---CACCAAGGTGGCGCCAAC---------------------------
OrNilTFS9b   ------CAATAT---GACTATTCA---GAC---CACCAAAGTGGTGCCAAC---------------------------
CiMonTFS9    ------CAGTAT---GACTATTCA---GAC---CACCAAAGTGGTGCCAAC---------------------------
CySemSHB     ------CAGTAT---GACTATTCA---GAC---CACCAAAGTGGTGCCAAC---------------------------
OrLatSCC     ------CAGTAT---GACTATTCA---GAC---CACCAAAATAGTGCCAAC---------------------------
GaAcuTFS9b   ------CAGTAT---GACTATTCA---GAT---CACCAAGGCGGCGCCAAC---------------------------
SaSalTFS2    ------CAGTAT---GACTATTCT---GAT---CACCAGGGCGGCGCCAAC---------------------------
GaMorS2      ------CAGTAC---GACTACTCA---GAA---CACCAGGCGGCCGCAAACTCCTCCAGCGGCACCGCTAACTCCTCC
AsMexTFS9b   ------CAGTAC---GACTACGCC---GAC---CACCACAGC------AAC---------------------------
ClGarS       ------CAGTAT---GATTACAGT---GAC---CACCAGGGCAGTGCTGCC---------------------------
CyCarHTF13   ------TACTAC---AACTACACC---GAG---CACCAGAGCCCCGCCGGC---------------------------
ClGarS1      ------CAGTAC---GACTACACC---GAGCAGCCAGCCGGCTCCGCG------------------------------
DaRerHBTF1   ------CAGTATACAGAGTACAGC---GAGCAGCACAGC---------------------------------------

         1795
HoSapSSDR3   ------------------------------TCCTACTACAGCCAC---------GCGGCAGGCCAGGGC---ACCGGC
MuMusSCG1    ------------------------------TCCTACTACAGTCAC---------GCAGCCGGCCAGGGC---TCAGGG
CaLupS1      ------------------------------TCCTACTACAGCCAC---------GCGGCGGGCCAGGGC---TCCAGC
GaGalSTF2    ------------------------------TCCTACTACAGCCAC---------GCCGCCGGCCAGAGC---GGAAGC
CoJapSSDRY   ------------------------------TCCTACTACAGCCAC---------GCTGCCGGGCAGAGC---GGAGGG
AlMisS1      ------------------------------TCCTACTACAGCCAC---------GCTGCCGGCCAGAGC---ACCAGC
TrScrHCC     ------------------------------TCCTACTACAGCCAT---------GCGGCCAGCCAGAGC---ACCAGT
LeOliS       ------------------------------TCCTACTACAGCCAT---------GCGGCCAGCCAGAGC---ACCAGC
AnPlaSSDRY   ------------------------------TCCTACTACAGCCAC---------GCCGCCGGGCAGAGC---AGCAGC
LaChaTFS9    ------------------------------TCTTACTACAGCCAT---------GCGGCAGGCCAGAGT---TCCAAC
XeLaeTFS1    ------------------------------ACCTATTACAGTCAT---------GCAAGTGGCCAGAAT---TCTGGT
BuBufTFSP    ------------------------------TCTTATTACAGTCAC---------GCAAGCGGTCAGAAT---TCTGGT
XeSilTFS9    ------------------------------TCTTATTACAGTCAC---------GCAAGCGGTCAGAAT---TCTGGT
BuGarS       ------------------------------ACCTACTATAGCCAT---------GCTGCAGGCCAGAGC---TCCAAC
RhMarS       ------------------------------ACCTACTATAGCCAT---------GCTGCAGGCCAGAGC---TCCAAC
GlRugSAA     ------------------------------TCTTATTACAGCCAC---------GCCGCTGGCCAAAGC---TCAAGC
PlWalS       ------------------------------ACCTACTACAGCCAC---------GCTGCTGGCCAGAGC---TCCAAC
ScCanS1      ------------------------------TCCTATTACAGCCAT---------GCTGGGGGGCAAACC---ACAGGC
DaRerHBTFS   ------------------------------TCCTATTACACCCAT------GCTGGA---GGTCAGAGC---TCCGGG
MoAlbHBTF1   ------------------------------TCCTACTACAGTCAT------GCGGGGGCGGGGCAGAGT---CCGGGG
OrNilTFS9a   ------------------------------TCCTACTACAGCCAC------GCGGGGGCGGGGCAGAGC---TCGGGG
GaAcuTFS9a   ------------------------------GGCTACTACAGCCAC------GCGGGGGCGGGGCAAGGC---TCAGGG
XiMacTFS9a   ------------------------------TCCTACTACAGCCACGCGGGGGCAGGGGCGGGTCAGGGC---TCCGGT
TaRubTFS9b   ------------------------------TCCTACTACAGCCAC------GCAGGGGCGGGGCAGGGG---GTCGGG
TeNigTFS9a   ------------------------------TCCTACTACAGCCAC------GCCGGGGCGGGTCAGGCC---TCGGGG
OrLatSCC1    ---------------------------------TTCTACAGCCCT------GCAGGGGCGGGGCAGGGT---TCTGGG
```

```
AsMexTFS9a  ------------------------------TCATACTACAGCCAC---------GCCGCAGGTCAAGGC---TCCGGG
GaMorS1     ------------------------------TCGTACTACGGCCAG------------------------------GGC
MoAlbHBTFS  ------------------------------TCCTACTACAGCCAT---------GCAGCTGGCCAAGGC---TCCGGC
OdBonTFS    ------------------------------TCCTACTACAGCCAT---------GCAGCTGGCCAAGGC---TCCAGC
PoRetS      ------------------------------TCTTACTACAGCCAT---------GCAGCTGGCCAAGGT---TCCAGC
XiMacTFS9b  ------------------------------TCTTACTACAGCCAT---------GCAGCTGGCCAAGGT---TCCAGC
EpCoiS2     ------------------------------TCCTACTACAGCCAC---------GCGGCGGGCCAAAGC---TCCGGC
TaRubTFS9a  ------------------------------TCCTACTACAGCCAC---------GCAGCGGGCCAGGGC---TCTGGG
TeNigTFS9b  ------------------------------TCCTACTACAGCCCC---------ACGACGGGCCAGGGC---TCCGGG
OrNilTFS9b  ------------------------------TCCTACTACAGTCAT---------GCAGGTGGCCAAGGC---TCAAGC
CiMonTFS9   ------------------------------TCCTACTACAGCCAT---------GCAGCTGGCCAAGGC---TCAAAC
CySemSHB    ------------------------------TCCTACTACAGCCAT---------GCAGCCGGTCAGGGC---TCCGGT
OrLatSCC    ------------------------------TCTTACTACAGCCAT---------GCAGCTGGTCAAGGC---TCCAAC
GaAcuTFS9b  ------------------------------TCCTACTACAGCCAG---------AGC------CAGGGC---TCCGGC
SaSalTFS2   ------------------------------TCTTATTATAGCCAT---------GCAGGTGCCCAAGGC---TCAGGG
GaMorS2     TCCGCCGCCGCCGCGACCGCCGCCGCGTCCTCCTACTAC------------------GGCGGGCAGGGCTCCTCCGGC
AsMexTFS9b  ------------------------------TCGTACTACAGCCAC---------GCCGCCGGCCAAGGC---TCCGGC
ClGarS      ------------------------------TCCTACTACAAC---------------------CAAGGT---TCAGGG
CyCarHTF13  ------------------------------TCCTACTACAGCCAG---------------------------------
ClGarS1     ------------------------------TCCTATTACTCGGTG---------------GGCCAGGGC------TCG
DaRerHBTF1  ------------------------------GCCTACTACAGCCCG---------------------------------

         1873
HoSapSSDR3  CTCTACTCCACCTTCACCTACATG------AACCCC---------------GCTCAGCGCCCCATGTACACCCCCATC
MuMusSCG1   CTCTACTCCACCTTCACTTACATG------AACCCC---------------GCGCAGCGCCCCATGTACACCCCCATC
CaLupS1     CTCTACTCCACCTTCACCTACATG------AACCCC---------------GCGCAGAGGCCCATGTACACCCCCATC
GaGalSTF2   CTTTACTCCACCTTCACCTACATG------AACCCC---------------ACGCAGCGCCCCATGTACACCCCAATC
CoJapSSDRY  CTCTACTCCACCTTCACCTACATG------AACCCC---------------ACGCAGCGTCCTATGTACACCCCCATT
AlMisS1     CTCTACTCCACCTTCACCTACATG------AACCCC---------------ACCCAGAGGCCGATGTACACCCCCATT
TrScrHCC    TTCTATTCCACCTTCACGTACATG------AACCCC---------------GCCCAAAGGCCCATGTACACGCCCATT
LeOliS      TTCTATTCCACCTTCACGTACATG------AAC------------------CACCAAAGGCCCATGTACACGCCCATT
AnPlaSSDRY  CTGTACTCCACCTTCACCTACATG------AACCCC---------------ACGCAGCGCCCCATGTACACCCCCATC
LaChaTFS9   CTATACTCGACTTTTACCTATATG------AGCCCC---------------ACCCAGCGTCCAATGTACACCCCCATT
XeLaeTFS1   CTCTACTCCACCTTTAGCTACATG------AATCCA---------------AGCCAACGCCCCTTGTACACCCCTATT
BuBufTFSP   CTCTACTCCAACTTTAGCTACATG------AATCCA---------------AGCCAGCGCCCCATGTACACGCCTATT
XeSilTFS9   CTCTACTCCAACTTTAGCTACATG------AATCCA---------------AGCCAGCGCCCCATGTACACGCCTATT
BuGarS      CTCTACTCTACCTTCAGCTACATG------AACCCA---------------AGCCAGCGTCCCATGTACACTCCTATC
RhMarS      CTCTACTCCACCTTCAGCTACATG------AACCCA---------------AGCCAGCGTCCCATGTACACTCCTATC
GlRugSAA    CTCTACTCAACGTTCAGCTACATG------AACCCC---------------AGTCAACGCCCCATGTACACCCCCATT
PlWalS      CTCTACTCCACTTTCTCCTACATG------AACCCC---------------ACTCAGCGCCCCATGTATACCCCTATT
ScCanS1     CTGTACTCCACCTTTGCCTACACG------AGTCCA---------------GCCCAGCGTCCCATGTACACACCCATT
DaRerHBTFS  CTGTACTCCACCTTCAGCTACATG------AGCTCA---------------AGCCAGAGGCCCATGTACACACCCATT
MoAlbHBTF1  CTATACTCGACTTTCAGCTACATG------AGCAGCCCC------------AGCCAGAGGCCCATGTACACACCCATA
OrNilTFS9a  CTGTACTCAACTTTCAGCTACATG------AGCAGCCCA------------ACCCAGAGGCCCATGTACACGCCCATA
GaAcuTFS9a  CTGTACTCGACTTTCAGCTACATG------AGTAGCCCC------------AGCCAGAGGCCCATGTACACGCCGATA
XiMacTFS9a  CTGTACTCGACTTTCAGCTACATG------GGCAGCCCC------------AACCAGAGGCCCATGTACACGCCCATC
TaRubTFS9b  CTGTACTCAACTTTCAGCTACGTG------AGCAGCCCC------------AGCCAGAGGCCCATGTACACACCCATC
TeNigTFS9a  CTGTACTCAACGTTCAGCTATGTG------AGCAGCCCC------------AGCCAGAGGCCCATGTACACACCCATC
OrLatSCC1   CTGTACTCGACTTTCAGTTACATG------AGCAGCCCC------------AGCCAGAGGCCCATGTACACCCCCATC
AsMexTFS9a  CTCTACTCCACCTTCAGCTACGTG---------AGCCCC------------AGCCAGCGGCCCATGTACACCCCAATC
GaMorS1     ATGTTCTCGGCCTTCGGCTACATGCTCCCCGGCAGCCCCCCCGCCGGCCAGAGCCAGAGGCCCATGTACACGCCCATC
MoAlbHBTFS  CTCCATTCAACCTTCAGCTACATG------AACCCA---------------AACCAGAGGCCAATGTACACCCCAATC
OdBonTFS    CTGTACTCCACTTTCAGTTACATG------AGCCCC---------------AGCCAGAGGCCGATGTACACCCCAATT
PoRetS      CTGTACTCCACCTTCAGCTACATG------AGCCCC---------------AGCCAGAGGCCGATGTACACCCCAATA
XiMacTFS9b  CTGTACTCCACCTTCAGCTACATG------AGCCCC---------------AGCCAGAGGCCGATGTACACCCCAATA
EpCoiS2     CTGTACTCCACCTACAGCTACATG------AGCCCA---------------AGCCAGAGGCCGATGTACACCCCGATC
TaRubTFS9a  CTTTACTCCACCTTCAGCTACATG------AACCCC---------------AGCCAGAGGCCCATGTACACCCCCATC
TeNigTFS9b  CTCTACTCCACCTTCAGCTACATG------AACCCC---------------AGCCAGAGGCCCATGTACACCCCCATT
OrNilTFS9b  TTGTACTCCACCTTTAGCTACATG------AGCCCC---------------AACCAGAGGCCGATGTACACCCCAATT
CiMonTFS9   CTGTATTCCACCTTTAGCTACATG------AGCCCC---------------AGCCAGAGGCCAATGTATACCCCGATT
CySemSHB    CTCTACTCCACCTTCAGCTACATG------AGCTCC---------------AGCCAGAGGCCCATGTACACCCCGATC
OrLatSCC    ATGTACTCCACCTTCAGCTACATG------AGCCCC---------------AGCCAGAGGCCGATGTATACCCCCATC
GaAcuTFS9b  CTGTATTCCACT---TCGGCTACA------TGCCCC---------------AGCCAGAGGCCGATGTACACCCCCATC
SaSalTFS2   CTCTATTCCTTCAGCAGCTATATG------AGCCCC---------------AGCCAGAGGCCCATGTATACCCCCATC
GaMorS2     CTCTACGCCGGCTTCGGCTACATG------AACCCC---------------AGCCAGAGGCCCATGTACACCCCCATC
AsMexTFS9b  CTCTACTCCACCTTCAGCTACATG------AGTCCGGGA------------GCACAGAGGCCCATGTACACCCCGATC
ClGarS      CTCTACTCCACATTTAGCTACATG------AGTTCT---------------ACCCAGAGGCCCATGTACACTCCTGTC
CyCarHTF13  ---TACCCCGCCTTCAGCTAC---------------------------------CAGAGGCCCATGTACACCCCGATC
```

```
ClGarS1     CTTTATTCCACCTTTAACTACATG------AGTTCT---------------AGCCAGAGGCCCATGTACACCCCCATC
DaRerHBTF1  ---TACCCCACATTCAGCTACAGC---------------------------------AGACCCCCGTACACCCCTGCA

         1951
HoSapSSDR3  GCCGACACCTCTGGGGTCCCTTCCATCCCG---CAGACCCACAGCCCC---CAGCACTGGGAA------CAACCCGTC
MuMusSCG1   GCTGACACCTCCGGGGTCCCTTCCATCCCG---CAGACCCACAGCCCG---CAGCACTGGGAA------CAACCAGTC
CaLupS1     GCCGACACCTCCGGGGTCCCCTCCATCCCG---CAGACGCACAGCCCC---CAGCACTGGGAA------CAGCCTGTC
GaGalSTF2   GCAGACACGTCGGGGGTGCCAACTATCCCG---CAGACCCACAGCCCGCAACAGCACTGGGAA------CAGCCGGTC
CoJapSSDRY  GCAGACACGTCTGGGGTGCCCTCCATCCCG---CAGACCCACAGCCCG---CAGCACTGGGAA------CAGCCCGTC
AlMisS1     GCAGACACTTCTGGGGTCCCCTCCATCCCC---CAGACCCACAGCCCA---CAGCACTGGGAA------CAGCCAGTC
TrScrHCC    GCAGACACTACCGGAGTCCCTTCCATTCCC---CAGACCCACAGCCCA---CAGCACTGGGAA------CAGCCGGTC
LeOliS      GCAGACACTACCGGAGTCCCTTCCATTCCC---CAGACCCACAGCCCG---CAGCACTGGGAA------CAGCCTGTC
AnPlaSSDRY  GCAGACACTTCTGGGGTCCCCTCCATCCCC---CAGACCCACAGCCCG---CAGCACTGGGAA------CAGCCCGTC
LaChaTFS9   GCAGACACCGCGGGGGTGCCCTCTATCCCC---CAGACCCATAGCCCA---CAACACTGGGAA------CAGCCAGTC
XeLaeTFS1   GCAGACACAACAGGAGTTCCATCAATCCCC---CAGACACACAGCCCA---CAACACTGGGAG------CAGCCCGTG
BuBufTFSP   GCAGACACGACGGGAGTTCCATCCATCCCC---CAGACACACAGCCCA---CAACACTGGGAG------CAGCCTGTC
XeSilTFS9   GCAGACACGACGGGAGTTCCATCCATCCCC---CAGACACACAGCCCA---CAACACTGGGAG------CAGCCTGTC
BuGarS      GCAGACACGACGGGGGTCCCATCCATTCCC---CAGACACATAGCCCC---CAACATTGGGAG------CAACCAGTC
RhMarS      GCAGACACGACGGGGGTCCCATCCATTCCC---CAGACACATAGCCCC---CAACACTGGGAG------CAACCAGTC
GlRugSAA    GCAGACACGACGGGGGTCCCATCCATCCCC---CAAACCCACAGCCCC---CAACACTGGGAG------CAACCGGTC
PlWalS      GCTGACACAACAGGGGTTCCCTCCATTCCA---CAGACCCACAGCCCC---CAGCACTGGGAG------CAGCCAGTC
ScCanS1     GCTGACACCACAGGAGTCCCTTCCATCCCC---CAAACCCACAGTCCA---CAGCACTGGGAG------CAACCCGTG
DaRerHBTFS  GCTGACTCTACAGGGGTGCCCTCCATCCCTCAATCCAACCACAGTCCG---CAGCATTGGGAC---CAGCAGCCGGTG
MoAlbHBTF1  GCCGACACCACGGGGGTGCCCTCCATCCCCCAGAAC------AGCCCG---CAGCACTGGGAG---CAGGCTCCGGTT
OrNilTFS9a  GCCGACAACACAGGGGTGCCCTCCATCCCGCAGAAC------AGCCCG---CAGCACTGGGAT---CCAGCGCCGGTT
GaAcuTFS9a  GCCGACACCACGGGGGTCCCGTCCATCCCGCAGAGC------AGCCCG---CAGCACTGGGAG---CAGGCTCCGGTC
XiMacTFS9a  GCCGACAACGCCGGGGTGCCGTCCATCCCACAGGGC------AGCCCACAGCAGCACTGGGAG---CAGGCTCCGGTT
TaRubTFS9b  GCCGACAACACGGGTGTGCCCACCATCCCGCAGAGC------AGCCCG---CAGCACTGGGAG---CAGGCGCCAGTT
TeNigTFS9a  GCTGACAACACGGGTGTGCCCACCATCCCGCAGAGC------AGCCCG---CAGCACTGGGAG---CAGGCGCCAGTT
OrLatSCC1   GCCGACAACGCGGGGGTGCCGTCCATCCCCCAGGGC------AGCCCG---CAGCACTGGGAG---CAGGCGCCGGTC
AsMexTFS9a  ACCGATTCCGCCGGGGTGCCTTCCATTCCTTCAGCTGGCCACAGCCCC---CAGCACTGGGAC---CAGCAGCCTGTC
GaMorS1     GCCGACACCACCGGCGTGCCCTCGGTGCCCCAGAGCAGCCACAGCCCC---CAGCACTGGGAGCACCAGACCCCCGTC
MoAlbHBTFS  GCCGACACCACCGGGGTGCCCTCTGTGCCC---CAGACCCACAGTCCA---CAGCACTGGGAA---CAGCAGCCCATT
OdBonTFS    GCTGACAGCGCTGGGGTGCCCTCTGTGCCG---CAGACCCACAGTCCA---CAGCACTGGGAG---CAGCAGCCCATC
PoRetS      GCCGACAGCACCGGGGTGCCCTCTGTGCCG---CAGACCCACAGTCCA---CAGCACTGGGAG---CAGCAGCCTGTG
XiMacTFS9b  GCCGACAGCACTGGGGTGCCCTCTGTGCCG---CAGACCCACAGTCCA---CAGCACTGGGAG---CAGCAGCCGATT
EpCoiS2     GCTGACACCACCGGGGTGCCCTCTGTGCCC---CAGACCCACAGTCCG---CAGCACTGGGAG---CAGCAGCCCATT
TaRubTFS9a  GCCGACAACGCCGGGGTCCCCTCCGTTCCC---CAGACCCACAGTCCG---CAACACTGGGAG---CAGCAGCCCATT
TeNigTFS9b  GCCGACAATGCCGGGGTCTCCTCCGTCCCC---CAGACCCACAGTCTG---CAGCACTGGGAG---CAGCAGCCTATT
OrNilTFS9b  GCTGACACCACCGGGGTGCCCTCTGTGCCG---CAGACCCACAGTCCG---CAGCACTGGGAG------CAGCCCATT
CiMonTFS9   GCTGACACCACCGGGGTGCCCTCTGTGCCG---CAGACCCACAGTCCG---CAGCACTGGGAT------CAGCCCATT
CySemSHB    GCTGACACCACCGGCGTGCCCTCCGTGCCC---CAGACCCACAGCCCT---CAGCACTGGGAT---CAGCAGCCCATT
OrLatSCC    GCTGACAGTACTGGGGTGCCATCTGTGCCA---CAGACCCACAGTCCA---CAGCACTGGGAG---CAGCAGCCCATA
GaAcuTFS9b  GCCGACACCACCGGGGTGCCCTCCGTGCCG---CAGACCCAGAGTCCG---CAGCACTGGGAG---CAGCAGCCCATC
SaSalTFS2   GCCGACCCCACCGGAGTGCCCTCGGTGCCCACCCAGACCCACAGTCCA---CAGCACTGGGAG---CAGCAGCCTGTC
GaMorS2     GCCGACCCCACCGGAGTGCCCAGCGTGCCC---CAGACCCACAGTCCG---CAGCACTGGGAG---CAGCAGCCCGTC
AsMexTFS9b  GCCGACACCACCGGCGTACCGTCCGTGCCT---CAGACCCACAGTCCC---CAACACTGGGAG---CAGCAACCCGTC
ClGarS      ACTGATACCAGTGGTGTTCCGAATGTGCCT---CAGACCCACAGTCCC---CAG---TGGGAG---CAGCAGCCAGTC
CyCarHTF13  GCCGACACG---------------CTGCCG---CAGGCGCACAGTCCG---CAGCACTGGGAG---CAGCAGCCCGTC
ClGarS1     ACCGAGACCCAG---------------------TCAGGCCACAGCCCC---CAGCACTGGGAC---CAGCAGCCGGTC
DaRerHBTF1  GCGGCGGCGGAT---------------------ACTGCACACACA------CACCACTGGGAC---CCGCAGCCCGTC

         2029
HoSapSSDR3  TACACACAGCTCACTCGACCT
MuMusSCG1   TACACACAGCTCACCAGACCC
CaLupS1     TACACACAGCTCACCAGGCCT
GaGalSTF2   TACACGCAGCTCACCCGGCCT
CoJapSSDRY  TACACGCAGCTCACCCGGCCT
AlMisS1     TACACACAGCTCACTAGGCCA
TrScrHCC    TACACACAGCTTACTAGGCCA
LeOliS      TACACACAGCTCACTAGGCCA
AnPlaSSDRY  TACACACAGCTCACCAGACCC
LaChaTFS9   TATACACAACTCACCAGGCCT
XeLaeTFS1   TACACACAGCTCACAAGACCC
BuBufTFSP   TATACACAGCTCACCAGGCCC
XeSilTFS9   TATACACAGCTCACCAGGCCC
BuGarS      TACACACAGCTCACCAGGCCA
RhMarS      TACACACAGCTCACCAGGCCA
```

```
GlRugSAA     TACACACAGCTGACCAGGCCA
PlWalS       TATACACAGCTCACCCGGCCT
ScCanS1      TACACCACCCTGACCCGACCT
DaRerHBTFS   TACACGCAGCTGTCCAGACCA
MoAlbHBTF1   TACACCCAGCTCACCAGACCC
OrNilTFS9a   TACACCCAGCTCACCAGACCC
GaAcuTFS9a   TACACCCAACTCCCCAGGCCC
XiMacTFS9a   TACACCCAGCTAACCCGACCC
TaRubTFS9b   TACACCCAGCTCACCAGACCC
TeNigTFS9a   TACACCCAGCTCACCAGACCC
OrLatSCC1    TACACGCAGCTGACCCGGCCC
AsMexTFS9a   TACACACAGCTGACGAGACCC
GaMorS1      TACACCCAGCTCACCAGGCCC
MoAlbHBTFS   TACACACAGCTGTCCAGGCCA
OdBonTFS     TACACACAACTGTCCAGGCCC
PoRetS       TATACCCAGCTGACCCGGCCC
XiMacTFS9b   TACACACAACTGTCCAGGCCC
EpCoiS2      TACACACAGCTCTCCAGGCCA
TaRubTFS9a   TACACGCAGCTCTCCAGGCCA
TeNigTFS9b   TACACGCAGCTCTCCAGGCCA
OrNilTFS9b   TACACACAACTTTCCAGGCCC
CiMonTFS9    TACACACAACTCTCCAGGCCC
CySemSHB     TACACACAACTGTCCAGACCG
OrLatSCC     TACACCCAGCTGTCCAGACCG
GaAcuTFS9b   TACACACAGCTCTCCAGGCCT
SaSalTFS2    TACACCCAGCTCTCCAGGCCC
GaMorS2      TACACGCAGCTGTCCCGGCCC
AsMexTFS9b   TACACCCAGTTGTCTCGACCT
ClGarS       TATACCCAGCTCTCCCGGCCT
CyCarHTF13   TACACCCAGCTGTCCAGGCCC
ClGarS1      TACACACAGCTCTCCGGACCC
DaRerHBTF1   TACACACAGCTGTCCAGACCC
```
